# Three distinct patterns of mental health response following accidents in mountain sports: a follow-up study of individuals treated at a tertiary trauma center

**DOI:** 10.1007/s00406-024-01807-x

**Published:** 2024-05-10

**Authors:** Hanna Veronika Salvotti, Piotr Tymoszuk, Mathias Ströhle, Peter Paal, Hermann Brugger, Martin Faulhaber, Nicola Kugler, Thomas Beck, Barbara Sperner-Unterweger, Katharina Hüfner

**Affiliations:** 1 Department of Psychiatry, Psychotherapy, Psychosomatics and Medical Psychology, University Hospital for Psychiatry II, Medical University of Innsbruck, Innsbruck, Austria; 2 Present Address: Department of Neurosurgery, University Hospital of Regensburg, Regensburg, Germany; 3 Data Analysis as a Service Tirol, Wörgl, Austria; 4 Department of Anesthesiology and Critical Care Medicine, Bezirkskrankenhaus Kufstein, Kufstein, Austria; 5 Austrian Society of Mountain and High-Altitude Medicine, Mieming, Austria; 6 Department of Anesthesiology and Critial Care Medicine, Paracelsus Medical University, Salzburg, Austria; 7 Austrian Board of Mountain Safety (Österreichisches Kuratorium fur Alpine Sicherheit), Innsbruck, Austria; 8 International Commission for Mountain Emergency Medicine (ICAR MedCom), Kloten, Switzerland; 9 Institute of Mountain Emergency Medicine, Eurac Research, Bolzano/Bozen, Italy; 10 International Society of Mountain Medicine (ISMM), Montreal, Canada; 11 Department of Sport Science, University of Innsbruck, Innsbruck, Austria; 12 Department of Orthopedics and Traumatology, Medical University of Innsbruck, Innsbruck, Austria; 13 Medical Directorate, Innsbruck Regional Hospital, Innsbruck, Austria

**Keywords:** Mountain sport accident, Machine learning, Mental health, Post-traumatic growth, Post-traumatic stress disorder

## Abstract

The restorative effect of physical activity in alpine environments on mental and physical health is well recognized. However, a risk of accidents and post-accident mental health problems is inherent to every sport. We aimed to characterize mental health in individuals following mountain sport accidents requiring professional medical management. Adult victims of mountain sport accidents treated at the hospital of the Medical University of Innsbruck (Austria) between 2018 and 2020 completed a cross-sectional survey at least 6 months following the admission (median 44 months, *n* = 307). Symptoms of post-traumatic stress disorder (PTSD, PCL-5), anxiety, depression, and somatization (PHQ), resilience (RS-13), sense of coherence (SOC-9L), post-traumatic growth (PTGI), and quality of life (EUROHIS-QOL), as well as sociodemographic and clinical information, were obtained from an online survey and extracted from electronic health records. Mental health outcome patterns were investigated by semi-supervised medoid clustering and modeled by machine learning. Symptoms of PTSD were observed in 19% of participants. Three comparably sized subsets of participants were identified: a (1) neutral, (2) post-traumatic growth, and (3) post-traumatic stress cluster. The post-traumatic stress cluster was characterized by high prevalence of symptoms of mental disorders, low resilience, low sense of coherence, and low quality of life as well as by younger age, the highest frequency of pre-existing mental disorders, and persisting physical health consequences of the accident. Individuals in this cluster self-reported a need for psychological or psychiatric support following the accident and more cautious behavior during mountain sports since the accident. Reliability of machine learning-based prediction of the cluster assignment based on 40 variables available during acute medical treatment of accident victims was limited. A subset of individuals show symptoms of mental health disorders including symptoms of PTSD when assessed at least 6 months after mountain sport accident. Since early identification of these vulnerable patients remains challenging, psychoeducational measures for all patients and low-threshold access to mental health support are key for a successful interdisciplinary management of victims of mountain sport accidents.

**Supplementary Information:**

The online version contains supplementary material available at 10.1007/s00406-024-01807-x.

## Introduction

Physical activity in mountains, such as hiking, climbing, alpine skiing, or ski touring, are gaining increasing popularity, a trend that has been accelerated by the COVID-19 pandemic [1]. Outdoor physical activity in general and mountain sports in particular exert beneficial effects on physical and mental health [2–4]. However, mountain sports are also associated with the risk of accidents and injury [5, 6], which could consequently lead to development of post-traumatic stress disorder (PTSD) or other stress-related mental disorders.

Diagnostic symptoms of PTSD include re-experiencing (such as involuntary, intrusive memories, dreams, or flashbacks of the traumatic event, criterion B), avoidance (such as avoiding memories, thoughts or external reminders related to the traumatic event, criterion C), negative alterations in cognition and mood (criterion D), and hyperarousal (such as irritability, problems with sleep or concentration, criterion E) [7], which can occur following self-experiencing or witnessing of a traumatic event such as actual or threatened death, injury, physical, or sexual violence. PTSD may co-occur with symptoms of depression and anxiety and substance use disorders [8]. Although most individuals have experienced at least one traumatic event during their lifetime [9], the prevalence of PTSD in the general European population has been estimated between 0.38 and 6.67% [10]. Yet, up to 26% of acute physical trauma patients have been reported to develop PTSD [11].

So far it has proven difficult to reliably identify early predictors of PTSD development in accident victims. While some recent studies on specific populations have identified risk factors, such as pre-existing psychological distress or mental disorders [12], others have focused on the arousal and stress reaction during acute trauma and peri-traumatic period as candidate predictors [13]. These results require validation and further generalization before they can be implemented into routine care [14, 15]. Overall, combination of multiple demographic, clinical, and mental factors seem to yield more accurate PTSD predictions compared to single variables [16].

Resilience, i.e., the ability to recover from adversities, can protect an individual from developing PTSD and can help explain why most people do not develop PTSD despite the high prevalence of traumatic events in the general population [17]. Generally individuals who perform mountain sports display high resilience values [2]. Post-traumatic growth can occur in individuals struggling to overcome the consequences of a traumatic event and describes a psychological adjustment reaction with strengthened positive perceptions of self, others, one’s life, and meaning of the event [18]. Although both resilience and post-traumatic growth are salutogenic concepts, their exact link is still under investigation [19].

With the increasing popularity of mountain sports, it is key to characterize the mental health consequences of accidents during mountain sports. Prevention of PTSD or other mental health problems following an accident is essential to preserve the positive mental health effects of physical activity in a mountain environment despite the inherent accident risk. Available data on the consequences of accidents during mountain sports on mental health are scarce. Prior studies were performed on mountain guides, rescuers, or avalanche accident survivors, mostly lack clinical data, and focus predominantly on single aspects of mental health such as PTSD [20–25]. The multiple facets of mental health including pathological and salutogenic features following a mountain sport accident have not been characterized. Additionally, risk factors of mental health problems specific for mountain sport accidents in recreational athletes have not been reported so far. Identification predictors available during acute medical management of the accident victims would greatly facilitate management of mountain sport accident by early, targeted psychological and psychiatric interventions.

Here, we aimed to identify multiple facets of mental health in a sample of 307 individuals treated at the hospital of the Medical University of Innsbruck (Austria) after a mountain sport accident. Such patterns were investigated at least 6 months following the accident and defined by symptoms of PTSD, anxiety, depression, and somatization, resilience, sense of coherence, post-traumatic growth, and quality of life in a semi-supervised clustering analysis. Subsequently, by statistical hypothesis testing and machine learning, we searched for early sociodemographic and clinical predictors that characterize these mental health subsets.

## Methods

### Ethics

This study was conducted in accordance with the Declaration of Helsinki and European data policies. All participants gave electronically signed written informed consent to participate. Participants’ data were processed in anonymized form. The study protocol was approved by the ethics committee of the Medical University of Innsbruck (Approval No. 1472/2020).

### Participants

**Fig. 1:**
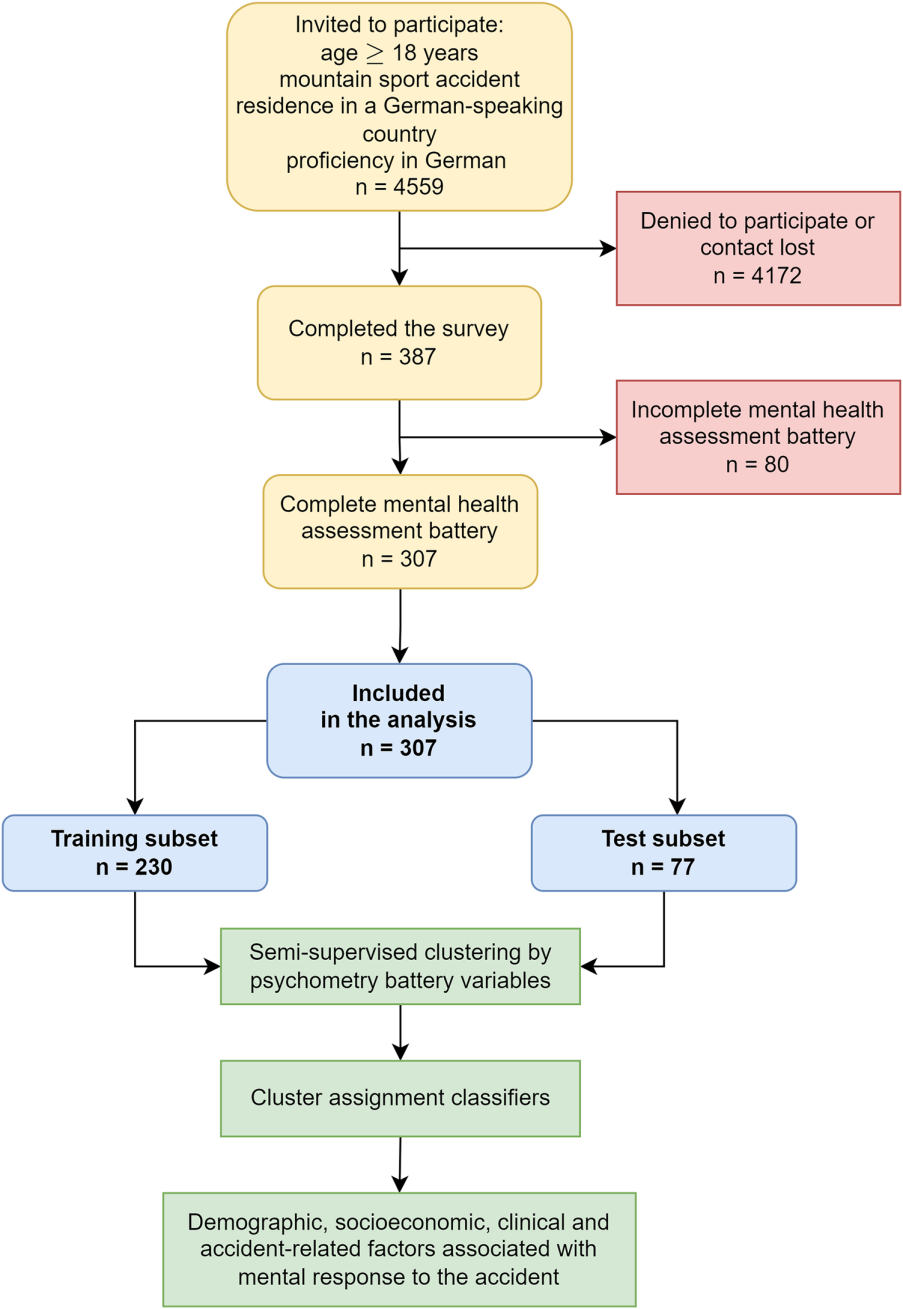
Flow diagram of the analysis inclusion process

Individuals treated following a mountain sport accident at the Department for Orthopedics and Traumatology of the hospital of the Medical University of Innsbruck between January 1, 2018 and December 31, 2020 were screened for participation. Individuals fulfilling the inclusion criteria––hospital admission at least 6 months prior to the start of the study, age ≥ 18 years, residence in a German-speaking regions (Germany, Austria, Switzerland and South Tyrol/Alto Adige in Italy), and proficiency in German (*n* = 4559)––were invited to participate in the online study survey via conventional mail. Out of the invited subjects, 387 completed the survey. Surveys of 307 individuals with the complete psychometry data were analyzed (overall response rate: 6.7%, Fig. 1, Supplementary Tables S1–S3).

### Procedures

Details on study procedures and variables are provided in Supplementary Methods and Supplementary Table S1.

Sociodemographic (e.g., age, sex, education, profession, income, prior mountain sport accidents and prior traumatic events), accident- and recovery-related (e.g., mode of rescue, psychological support, persistent physical health consequences), and psychometric variables were recorded with a cross-sectional online survey.

The psychometric battery consisted of German versions of assessment tools for symptoms of PTSD (PCL-5: PTSD checklist for DSM-5) [26], anxiety (GAD-7: 7-item general anxiety disorder scale) [27], depression (PHQ-9: 9-item patient health questionnaire for depressive symptoms) [28, 29], panic (PHQ-panic module) [28], somatization (PHQ-15: 15-item patient health questionnaire of common somatic symptoms as a substitute for somatization) [30], resilience (RS13: 13-item resilience scale) [31], sense of coherence (SOC-9L: Leipzig 9-item sense of coherence questionnaire) [32], quality of life (EUROHIS-QOL 8: 8-item EUROHIS project quality of life scale) [33], and post-traumatic growth (PTGI: post-traumatic growth inventory) [18]. Items of the PTGI and PTSD instruments, and questions concerning flashbacks were provided with captions indicating that the questions refer to the mountain sport accident of interest (‘The following questions refer to consequences of your mountain sport accident which occurred on…’). The tools displayed good-to-excellent consistency (McDonald’s *ω* > 0.8, Supplementary Tables S2 and S3).

Clinically significant symptoms of anxiety (GAD-7 ≥ 10), depression (PHQ-9 ≥ 10) [34], somatization (PHQ-15 ≥ 10) [30], and resilience classes (low: RS-13 0–65, moderate: 66–72, high: ≥ 73) [31] were defined with published cutoffs.

Separate scores were calculated for domains B, C, D, and E of the PCL-5 tool along with the total score being the sum of all items. Each PCL-5 item was scored as 0: not at all, 1: a little bit, 2: moderate, 3: quite a bit, and 4: extremely. Participants positive for the B domain or C domain PTSD symptoms were identified by at least one item per domain scored with ‘moderate’ or higher. Participants positive for the D or E domain PTSD symptoms were identified by at least two items per domain scored with ‘moderate’ or higher. Significant PTSD symptoms were assumed in participants screened positive for at least one of the B, C, D, or E PCL-5 domains. Manifest PTSD was considered for participants scoring positive for all four PCL-5 domains [26].

Traumatic events prior to the accident were assessed with the DIA-X tool [35]. Flashbacks of the accident of interest during mountain sport activity were surveyed as none, > 1/year, and > 1/month. Smoking was surveyed as a single yes/no question. Problematic alcohol use was investigated with the CAGE tool [36]. Data on the type of the mountain sport activity during the accident, injury diagnosis, severity (AIS: abbreviated injury scale) [37] and body location, hospitalization, surgery, and number of surgical ICD-10 diagnoses were extracted from electronic patient records.

The training and test participant subsets (3:1 size ratio) were obtained by random splitting which minimized differences in in sociodemographic, medical history, and clinical and accident- and injury-related variables assessed by Gower distance (Fig. 1). The training subset size (*n* = 230) was sufficient for clustering analysis as assessed by Hopkins metrics of 0.74 indicative of substantial spontaneous clustering tendency.

### Analysis endpoints

The primary analysis endpoint was identification of subsets of patients differing in symptoms of PTSD, anxiety, depression, somatization, resilience, sense of coherence, post-traumatic growth, and quality of life at least 6 months following a mountain sport accident. This endpoint was pursued by semi-supervised clustering analysis. The secondary analysis endpoint was comparison of sociodemographic and clinical characteristics of these newly defined subsets in a search for predictors of good or poor mental health following a mountain sport accident. The secondary analysis endpoint was addressed by statistical hypothesis testing and machine learning.

### Statistical analysis

Details on statistical analysis are provided in Supplementary Methods.

Statistical analysis was done with R version 4.2.3. Numeric variables are displayed as medians with interquartile ranges. Categorical variables are presented as percentages and counts. Differences in numeric variables were analyzed by Mann–Whitney or Kruskal–Wallis test with *r* or *η*^2^ effect size statistics. Differences in categorical variable distribution were assessed by *χ*^2^ test with Cramer *V* effect size statistic.

The training subset was clustered in respect to normalized median-centered psychometric scores by partition around medoids with cosine distance [38]. This algorithm had a good explanatory performance (ratio of between-cluster sum of squares to total sum of squares), separation between clusters (average silhouette width) [39], and the superior accuracy in tenfold cross-validation [40] as compared with the hierarchical clustering and KMEANS algorithms. The *k* = 3 cluster number choice was based on the bend of within-cluster sum of squares curve and maximal mean silhouette statistic. The training subset observations were assigned to the clusters with an inverse distance-weighted 27-nearest neighbor classifier.

Multi-parameter machine learning classifiers of the mental health cluster assignment were trained with the canonical random forest algorithm [41], regularized neural network with a single hidden layer [42], support vector machine algorithm with radial kernel [43], recursive partitioning [44], multinomial elastic net regression [45], and conditional random forest algorithm [46]. The cluster assignment was predicted for the test subset and the classifiers’ performance at predicting the cluster assignment was assessed by the accuracy and $\kappa$ statistics, Brier score, as well as sensitivity and specificity of predicted assignment to the post-traumatic stress cluster.

## Results

### Characteristic of the study cohort

Surveys with complete psychometric data from 307 individuals were analyzed (Fig. 1). The median time between the trauma center admission after the accident and the survey completion was 1343 days (interquartile range 804–1441 days). As compared with the analyzed participants, patients who did not respond to the study invitation were characterized by a significantly higher frequency of biking accidents and lower rate of alpine ski/snowboard accidents (*V* = 0.096, *p* < 0.001). As compared with the analyzed participants, the non-responders were also characterized by significantly more frequent minor and less frequent severe injuries (*V* = 0.16, *p* = 0.0019), overall lower injury grade (*r* = 0.15, *p* < 0.001), significantly lower rates of hospitalization (*V* = 0.14, *p* < 0.001) and surgery (*V* = 0.12, *p* < 0.001), as well as significantly lower surgery complexity reflected by surgical diagnosis numbers (*V* = 0.12, *p* < 0.001). Effect sizes of those differences were small (Supplementary Table S4). The survey participants excluded from the analysis due to incomplete psychometric data had significantly lower income (*V* = 0.22, *p* < 0.001), less severe injury grade (*r* = 0.11, *p* = 0.031), more frequent injuries of the upper limbs (*V* = 0.13, *p* = 0.019), less frequently required hospitalization (*V* = 0.12, *p* = 0.03) or surgery (*V* = 0.11, *p* = 0.045), and suffered less frequently from persistent physical health consequences of the accident than the analyzed participants (*V* = 0.12, *p* = 0.037). Effect sizes of the differences between the included and excluded participants were small (Supplementary Table S5).

The analyzed participants were predominantly middle aged (median: 51, interquartile range 33–60 years) and 45% of them were females. The vast majority had secondary or tertiary education grade (83%) and were professionally active (68%). Less than 8% of participants worked in a search and rescue or mountain sport profession. Annual household incomes of ≥ 45,000 Euro were reported by 42% of participants. Less than 10% of participants were smokers or at risk of problematic alcohol use defined as CAGE ≥ 2. Pre-existing physical disorders were reported by 15% of participants with cardiovascular, neurological, and metabolic illness being the most frequent. Mental disorders diagnosed by a health care professional prior to the accident affected 5.2% of the cohort, with affective (2.3%) and somatoform disorders (1.6%) as the leading pre-existing mental disorders. Four of ten participants had experienced or witnessed a traumatic event prior to the accident, 10.4% participants had been exposed to two or more traumatic events (Table 1). The age structure was similar to the general Austrian population [47]. Males (*V* = 0.00078, *p* = 0.041), individuals with secondary and tertiary education (*V* = 0.0059, *p* < 0.001), as well as professionally active people and students (*V* = 0.0027, *p* < 0.001) were more frequent in the investigated sample than in the general Austrian population [48, 49]. Active smoking (*V* = 0.0027, *p* < 0.001) and physical or mental disorders were significantly less prevalent in the study cohort (*V* = 0.0025, *p* < 0.001). Effect sizes of these significant differences were small (Supplementary Table S6).

**Table 1:** Demographic and socioeconomic characteristics of the study cohort. Numeric variables are presented as medians with interquartile ranges (IQR). Categorical variables are presented as percentages and counts within the complete observation set

Variable^a^	Statistic^b^
Participants, *n*	307
Hospital visit—survey time, days	1300 [IQR: 800–1400] Range 390–1600
Age at accident, years	51 [IQR: 33–60] Range 18–82
Age at accident, class, years	18–30: 20% (*n* = 61) 31–65: 66% (*n* = 202) > 65: 14% (*n* = 44)
Sex	Female: 45% (*n* = 137) Male: 55% (*n* = 170)
Residence in the Alps	73% (*n* = 225)
Highest education grade	Primary: 16% (*n* = 49) Secondary: 38% (*n* = 115) Tertiary: 45% (*n* = 136)
Employment at the accident	Employed: 68% (*n* = 210) Unemployed: 3.6% (*n* = 11) Student: 10% (*n* = 32) Retired: 18% (*n* = 54)
Mountain sport profession	5.2% (*n* = 16)
Search and rescue profession	7.2% (*n* = 22)
Income/year	No income: 21% (*n* = 63) < 30,000 EUR: 18% (*n* = 56) 30,000–45000 EUR: 19% (*n* = 59) ≥ 45,000 EUR: 42% (*n* = 129)
Smoking	7.8% (*n* = 24)
Problematic alcohol use (CAGE ≥ 2)	9.4% (*n* = 29)
Pre-existing physical illness type	None: 85% (*n* = 260) CVD: 2.9% (*n* = 9) Neurological: 1.3% (*n* = 4) Metabolic: 1.3% (*n* = 4) Pulmonary: 0.65% (*n* = 2) Cancer: 0.65% (*n* = 2) Rheumatoid: 0.33% (*n* = 1) Skin: 0.33% (*n* = 1) Other: 7.8% (*n* = 24)
Number of prior traumatic events/DIA-X	None: 60% (*n* = 183) 1: 30% (*n* = 92) 2: 7.5% (*n* = 23) 3 + : 2.9% (*n* = 9)
Pre-existing diagnosed mental disorder	5.2% (*n* = 16)
Type of pre-existing diagnosed mental disorder	Affective disorder: 2.3% (*n* = 7) Personality disorder: 0.33% (*n* = 1) Post-traumatic stress disorder: 0.65% (*n* = 2) Somatoform disorder: 1.6% (*n* = 5) Anxiety disorder: 0.65% (*n* = 2) Attention-deficit hyperactivity disorder: 0.33% (*n* = 1) Addiction: 0.33% (*n* = 1) Bulimia nervosa: 0.33% (*n* = 1)

^a^CAGE: Cut/Annoyed/Guilty/Eye substance abuse scale; DIA-X: Diagnostic Expert System, traumatic event score

^b^EUR: Euro; CVD: cardiovascular disease

Mountain sport accidents in the past were reported by 38% of participants. Most of the investigated accidents occurred during alpine skiing or snowboarding in secured areas of ski resorts (59%) followed by biking (16%), hiking (5.6%), cross-country skiing (5.6%), and sledding (4%). Mountain sports perceived as particularly risk associated such as alpine skiing/snowboarding in non-secured areas (3%), ice climbing (0.33%), climbing (3%), mountaineering (0.66%), and paragliding (0.33%) comprised a minute fraction of the study cohort (Table 2). As compared with the Austrian statistic of mountain accidents in 2023 (which contains accidents in which professional rescue service or the alpine police was involved in some way [50]), young adults aged ≤ 30 years were significantly fewer in the study cohort (*V* = 0.025, *p* = 0.034). Frequency of alpine skiing/snowboarding, cross-country skiing, biking, and water sport accidents was significantly higher in the analyzed sample as compared with the national registry. Percentages of ski/snowboard accidents in non-secured areas and hiking accidents were in turn significantly lower in the study cohort than in the national registry (global difference: *V* = 0.14, *p* < 0.001). Effect sizes of the differences between the study sample and the national registry were small (Supplementary Fig. S1).

**Table 2:** Characteristics of the sport accident, injury, psychological management, and accident consequences. Numeric variables are presented as medians with interquartile ranges (IQR). Categorical variables are presented as percentages and counts within the complete observation set

Variable	Statistic
Prior mountain sport accidents	38% (*n* = 118) *n* = 307
Mountain sport type^a^	Alpine skiing/snowboarding: 59% (*n* = 180) Ski touring/freeride: 3% (*n* = 9) Cross-country skiing: 5.6% (*n* = 17) Sledding: 4% (*n* = 12) Ice climbing: 0.33% (*n* = 1) Hiking: 5.6% (*n* = 17) Climbing: 3.6% (*n* = 11) Mountaineering: 0.66% (*n* = 2) Biking: 16% (*n* = 48) Air sports: 0.33% (*n* = 1) Water sports: 0.33% (*n* = 1) Other: 1.3% (*n* = 4) *n* = 303
Alone during the accident	32% (*n* = 97) *n* = 307
Responsible for the accident	Self: 77% (*n* = 237) Non-self: 23% (*n* = 70) *n* = 307
Number of injured persons	Only self: 64% (*n* = 195) Self and partner: 3.6% (*n* = 11) 3 + persons: 1.3% (*n* = 4) No information: 32% (*n* = 97) *n* = 307
Rescue mode	Self: 50% (*n* = 155) Companion: 21% (*n* = 63) Rescue team: 29% (*n* = 89) *n* = 307
Injury severity class, AIS^b^	1: 37% (*n* = 108) 2: 35% (*n* = 103) 3 + : 28% (*n* = 83) *n* = 294
Hospitalized	26% (*n* = 80) *n* = 307
Surgical therapy	14% (*n* = 43) *n* = 307
Psychological/psychiatric support post-accident	9.1% (*n* = 28) *n* = 307
Psychological/psychiatric support need post-accident	7.5% (*n* = 23) *n* = 307
Physical health consequences of the accident	37% (*n* = 115) *n* = 307
Returned to same mountain sport post-accident	85% (*n* = 262) *n* = 307
Caution during mountain sport post-accident	No change: 35% (*n* = 106) More cautious: 65% (*n* = 199) Less cautious: 0.65% (*n* = 2) *n* = 307
Flashback frequency during mountain sport	None: 60% (*n* = 185) > 1/year: 22% (*n* = 68) > 1/month: 18% (*n* = 54) *n* = 307

^a^alpine skiing/snowboarding: alpine skiing or snowboarding in secured areas of ski resorts; ski touring/freeride: skiing or snowboarding outside of secured areas of ski resorts; sledding: sledding and bob sledding; ice climbing: ice and mixed climbing; climbing: bouldering, sport, and rock climbing; mountaineering: high mountain and glacier tours; biking: mountain, cross-country, and road cycling; air sport: paragliding

^b^AIS: abbreviated injury scale

One-third of participants were alone during the accident (32%) and, in most cases, were the only person responsible to the accident (77%). Professional rescue service was involved in 29% of the accidents. In 35% of participants the injury severity was moderate (AIS 2) and in 28% severe to critical (AIS ≥ 3). Limb injuries were the most common followed by injuries of the head and face (Supplementary Fig. S2A). Hospitalization and surgery rates were 26% and 14%, respectively. Psychological or psychiatric support after the accident was provided to 9.1% individuals. A subset of participants (7.5%), who had not received psychological or psychiatric support, declared a need for psychological or psychiatric support following the accident. Persisting physical consequences of the accident were reported by 37% of participants and flashbacks of the accident of interest during mountain sport at the time of the questionnaire completion were observed in 40% of the cohort. Although most individuals returned to the same mountain sport following the accident (85%), 65% of all participants described their behavior during mountain sport as more cautious (Table 2).

PTSD symptoms related to the mountain sport accident defined as positive scoring of at least one of the B, C, D, or E domains of the PCL-5 tool were observed in 19% of participants. Concerning particular PCL-5 domains, positive scoring for the domain B was the most common (11%), and frequency of participants screened positive for the domain D was the lowest (5.2%). Solely four patients (1.3%) were screened positive for all four diagnostic criteria suggestive of manifest PTSD (Table 3, Supplementary Fig. S2BC). Clinically relevant symptoms of anxiety (2.9%), depression (7.2%), and somatization (5.9%) were rare in this cohort consisting of 68% highly resilient individuals (Table 3). Mean scoring of quality of life in the cohort was significantly higher than in the comparable general German population (cohort: 4.2, population estimate: 4.08, one-sample Wilcoxon test: *r* = 0.33, *p* < 0.001) [33]. The median total score of accident-related post-traumatic growth was 32 (interquartile range 16.5–48.5), with the relations domain with the highest rating and the spiritual domain with the lowest scores (Table 3, Supplementary Fig. S3). We could not observe any significant association of values of psychometric scores or frequency of mental health problems with the time between trauma center admission and survey completion (not shown).

**Table 3:** Mental health characteristics of the study participants at survey completion

Variable^a^	Statistic
Participants, *n*	307
GAD-7 score	1 [IQR: 0–3], range 0–15
Clinically relevant anxiety symptoms (GAD-7 ≥ 10)	2.9% (*n* = 9)
PHQ-9 score	2 [IQR: 1–5], range 0–16
Clinically relevant depression symptoms (PHQ-9 ≥ 10)	7.2% (*n* = 22)
PHQ-15 score	2 [IQR: 1–4], range 0–23
Clinically relevant somatization symptoms (PHQ-15 ≥ 10)	5.9% (*n* = 18)
EUROHIS-QOL 8 score	4.4 [IQR: 4–4.6], range 2–5
SOC-9L score	19 [IQR: 16–25], range 10–49
RS13 score	78 [IQR: 70–85], range 15–91
RS13 resilience class	Low: 18% (*n* = 56)
	Moderate: 14% (*n* = 42)
	High: 68% (*n* = 209)
PTGI score	32 [IQR: 16–48], range 0–100
PCL-5 score	3 [IQR: 1–7], range 0–44
PTSD symptoms (at least one PCL-5 domain positive)	19% (*n* = 58)

Numeric variables are presented as medians with interquartile ranges (IQR). Categorical variables are presented as percentages and counts within the complete observation set

^a^GAD-7: 7-item general anxiety disorder scale; PHQ-9: 9-item patient health questionnaire for depressive symptoms; PHQ-15: 15-item patient health questionnaire for common somatic symptoms as a substitute for somatization, EUROHIS-QOL 8: 8-item EUROHIS project quality of life scale; SOC-9L: Leipzig 9-item sense of coherence questionnaire; RS13: 13-item resilience scale; PCL-5: PTSD checklist for DSM-5; PTGI: post-traumatic growth inventory; PTSD: post-traumatic stress disorder

As compared with general population estimates [51–54], frequency of prior traumatic events and manifest PTSD in the study sample was low. The frequency of PTSD symptoms and of manifest PTSD measured with the PCL-5 instrument was, however, similar compared to a sample of Swiss mountain rescuers (Supplementary Fig. S4) [21]. Our sample was characterized by significantly lower percentages of clinically relevant symptoms of anxiety (GAD-7 ≥ 10, 2.9%, 95% CI 1.3–4.9%) and depression (PHQ-9 ≥ 10, 7.2%, 95% CI 4.6–10%) as compared with estimates for the general Austrian population in 2022 (anxiety, GAD-7 ≥ 10: 16%, 95% CI 14–18%, depression, PHQ-9 ≥ 10: 28%, 95% CI 26–31%, Supplementary Fig. S5A) [34]. The resilience scores in our study sample (mean: 76, SD: 12) were comparable with resilience levels in the Swiss mountain rescuer sample [21], and higher as compared with the general population estimates (mean: 72, SD: 12, Supplementary Fig. S5B) [55].

For clustering and modeling, the cohort was split into the training (*n* = 230) and test subset (*n* = 77). The sole significant differences between these subsets concerned resilience class distribution and scores of quality of life, their effect size was small (Supplementary Table S7).

### Three clusters of mental health response after mountain sport accidents

To identify patterns of mental health following mountain sport accidents, we subjected the participants to medoid clustering in respect to a broad range of psychometric scores (Supplementary Table S2). Three mental health clusters were identified in the training subset of the study cohort: ‘neutral,’ ‘PTG’ (post-traumatic growth), and ‘PTS’ (post-traumatic stress) and named after their key mental characteristic (Supplementary Fig. S6). Subsequently, the mental health cluster assignment could be validated in the test subset as evident from comparable fractions of explained clustering variance (training: 0.55, test: 0.52), comparable average silhouette statistics (training: 0.3, test: 0.26), similarly low frequency of potentially misclassified observations (fraction of observations with negative silhouette widths, training: 0.043, test: 0.078), and good preservation of the nearest neighborhood (fraction of 5-nearest neighbors in the same cluster, training: 0.89, test: 0.8). Furthermore, classification of the training and test individuals yielded similar distribution of cluster sizes, good visual cluster separation, and high similarity between the corresponding clusters in the training and test cohort subsets (Supplementary Figs. S7–S9).

**Fig. 2:**
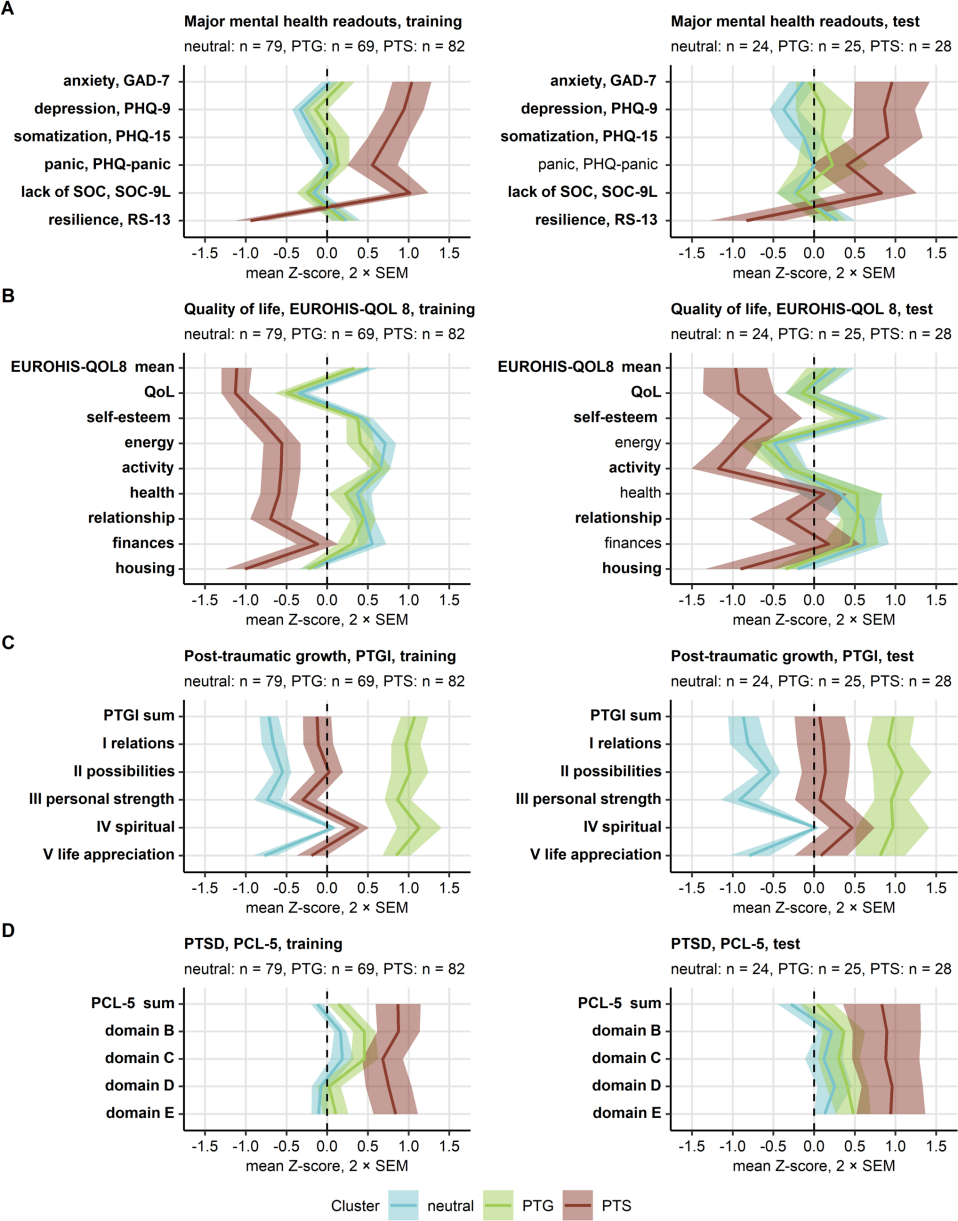
Cluster-defining scores of symptoms of mental disorders, sense of coherence, resilience, quality of life, post-traumatic growth, and post-traumatic stress disorder in the mental health clusters. Study participants in the training subset of the cohort were assigned to the neutral, PTG (post-traumatic growth), and PTS (post-traumatic stress) mental health clusters defined in respect to psychometric scores by the partition around medoids (PAM) algorithm. Study participants in the test subset of the cohort were assigned to the mental health clusters by the inverse distance-weighted 27-nearest neighbor classifier. Differences in the cluster-defining psychometric scores between the clusters were assessed by Kruskal–Wallis test with $\eta^2$ effect size statistic. *P* values were adjusted for multiple testing with the false discovery rate method. Mean normalized scores of major mental health readouts, sense of coherence and resilience (**A**), quality of life (**B**), post-traumatic growth (**C**), and symptoms of post-traumatic stress disorder (PTSD, **D**) in the mental health clusters of the training and test subsets of the study cohort are depicted as solid lines. Tinted regions represent 2 × SEM (standard error of the mean). Numbers of observations in the clusters are displayed in the plot captions. Significant effects are highlighted in bold. GAD-7: 7-item general anxiety disorder scale; PHQ-9: 9-item patient health questionnaire for depression symptoms; PHQ-15: 15-item patient health questionnaire for common somatic symptoms as a substitute for somatization; EUROHIS-QOL 8: 8-item EUROHIS project quality of life scale; SOC-9L: Leipzig 9-item sense of coherence questionnaire; RS13: 13-item resilience scale; PCL-5: PTSD checklist for DSM-5; PTGI: post-traumatic growth inventory; PTSD: post-traumatic stress disorder

The mental health clusters were of approximately equal size but differed significantly in psychometric characteristic. The neutral cluster was characterized by low scores of PCL-5 domains assessing PTSD symptoms as well as low ratings of anxiety, depression, panic, somatization, and post-traumatic growth along with high ratings of sense of coherence, resilience, and quality of life. The PTG cluster demonstrated similarly low scores of major mental health disorders, high resilience, and sense of coherence. Its key characteristics were the highest levels of post-traumatic growth. The remaining PTS cluster displayed the highest scores of PTSD symptoms measured by domains of the PCL-5 instrument, as well as the peak ratings of anxiety, depression, somatization, poor sense of coherence, low resilience, and low quality of life. Post-traumatic growth scores in the PTS cluster were higher than in neutral but lower than in PTG cluster participants (Fig. 2, Supplementary Fig. S9).

**Fig. 3:**
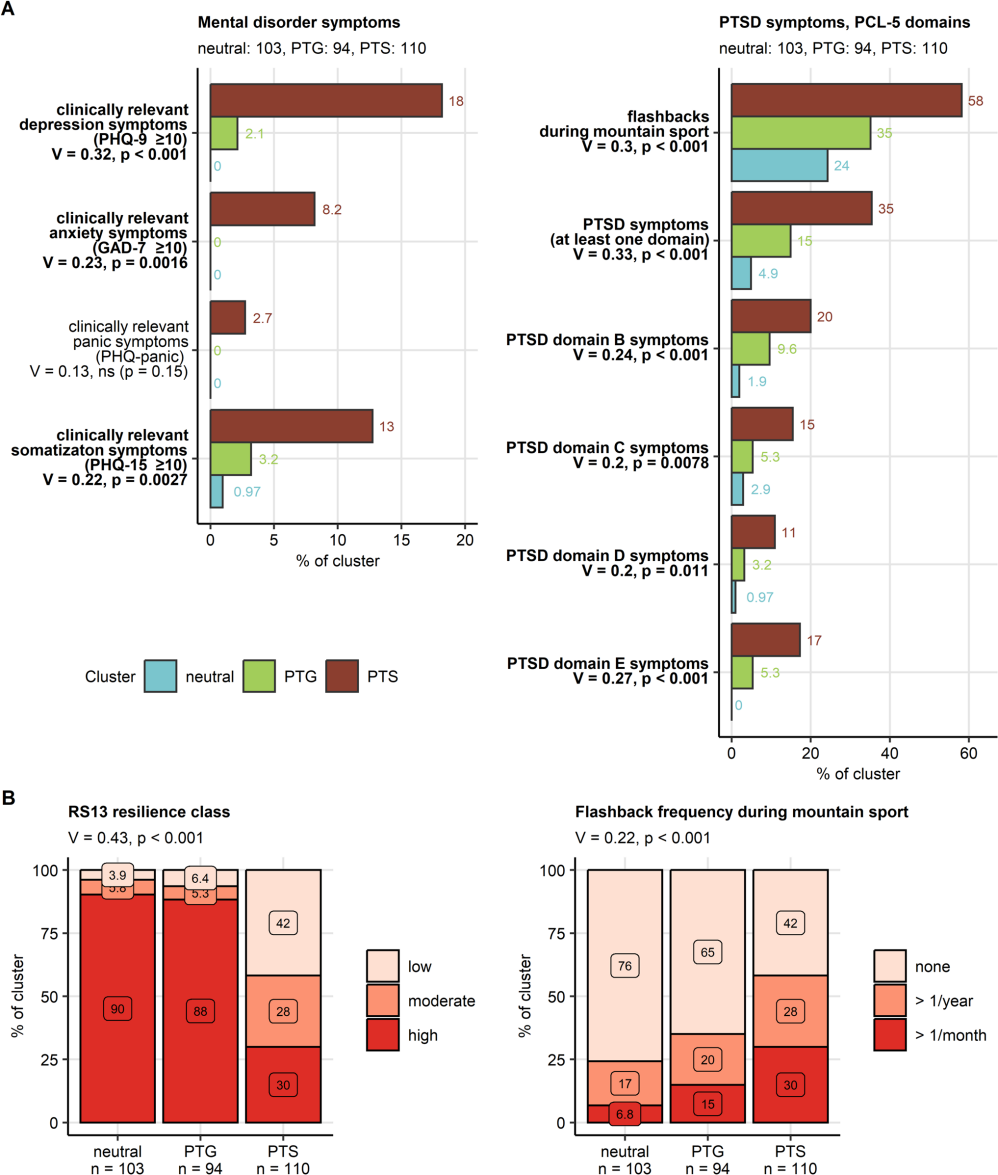
Symptoms of mental disorders and resilience classes in the mental health clusters. Differences in frequency of symptoms of common mental disorders and symptoms of post-traumatic stress disorder (PTSD), flashbacks, and distribution of resilience classes between the mental health clusters (neutral, post-traumatic growth [PTG] and post-traumatic stress [PTS]) were investigated in the entire study cohort by $\chi^2$ test with Cramer’s *V* effect size statistic. *P* values were adjusted for multiple testing with the false discovery rate method. **A** Symptoms of common mental disorders, and symptoms of post-traumatic stress disorder (PTSD). Percentages of affected individuals in the mental health clusters are presented in bar plots. Effect sizes and *P* values are indicated in the *Y* axes. Significant effects are highlighted in bold. Numbers of observations in the clusters are indicated in the plot captions. **B** Resilience classes and frequency of flashbacks during sport activity in the mental health clusters presented in stack plots. Effect sizes and *P* values are displayed in the plot captions. Numbers of observations in the clusters are indicated in the *X* axes. GAD-7: 7-item general anxiety disorder scale; PHQ-9: 9-item patient health questionnaire for depressive symptoms; PHQ-15: 15-item patient health questionnaire for common somatic symptoms as a substitute for somatization; RS13: 13-item resilience scale; PCL-5: PTSD checklist for DSM-5; PTSD: post-traumatic stress disorder

Clinically relevant symptoms of anxiety (PTS: 8.2%, *V* = 0.23, *p* = 0.0016), depression (PTS: 18%, *V* = 0.32, *p* < 0.001), and somatization (PTS: 13%, *V* = 0.22, *p* = 0.0027) were significantly more frequent in the PTS cluster as compared with the PTG or neutral clusters. Furthermore, frequencies of low (PTS: 42%) and moderate resilience classes (PTS: 28%, *V* = 0.43, *p* < 0.001) peaked in the PTS cluster. Symptoms of PTSD, frequency of flashbacks, as well as percentages of individuals screening positive for each of the B, C, D, and E PCL-5 domains were significantly higher in the PTS as compared with the remaining clusters. Analogically, the frequency of participants with symptoms of PTSD defined by at least one positive PCL-5 domain was the highest in the PTS cluster (35%, *V* = 0.33, *p* < 0.001, Fig. 3, Supplementary Table S9).

Effects of missing observations, year of the accident as a readout of the accident—survey time, as well as factors of potential importance for mental health such as annual income, prior mountain sport accidents, injury severity, and hospitalization were investigated by sensitivity analysis (Supplementary Methods) [56]. Those factors had only a minor influence on clustering as assessed by fractions of explained clustering variance (genuine data set: 0.55, sensitivity analysis: 0.52–0.58) or cluster size distribution (Supplementary Fig. S10). In the clustering of the study cohort appended with incomplete observations, the participants with missing psychometric data which had been successfully imputed were preferentially assigned to the PTS cluster (imputed observations in the PTS cluster: *n* = 13, total imputed: *n* = 18, Supplementary Fig. S11).

### Demographic, socioeconomic, and clinical characteristics of the mental health clusters

Among 48 investigated demographic, socioeconomic, clinical, accident-, and recovery-related variables, only 6 features (age, pre-existing physical illness, pre-existing diagnosed mental disorder, need for psychological or psychiatric support after the accident, persistent physical health consequences of the accident, and caution during mountain sport following the accident) were found to differ significantly between the mental health clusters. The effect size of these differences was small (Supplementary Table S10).

**Fig. 4:**
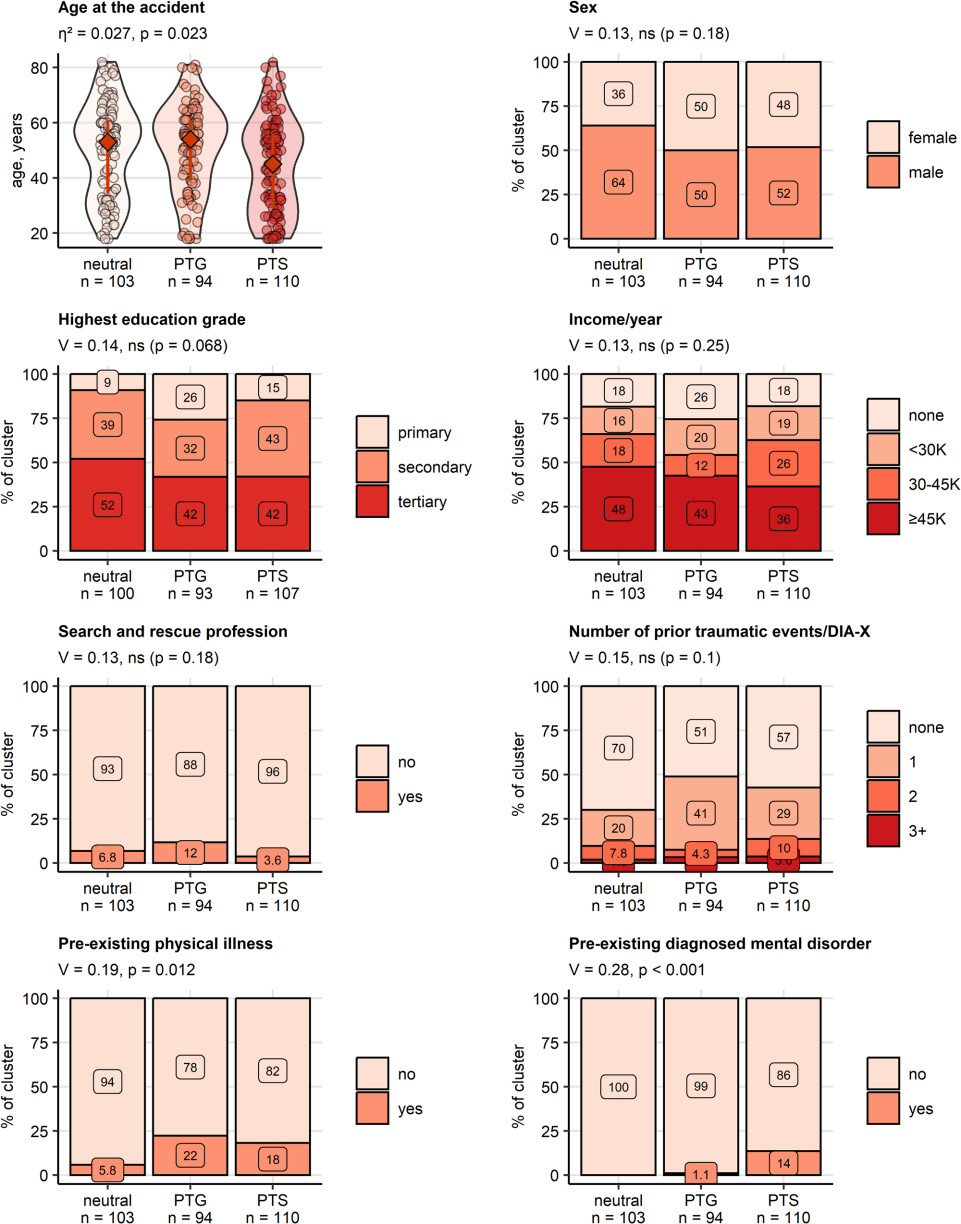
Sociodemographic and medical history characteristics of the mental health clusters. Differences in age at the time of the accident between the mental health clusters (neutral, post-traumatic growth [PTG], post-traumatic stress [PTS]) were assessed in the entire cohort by Kruskal–Wallis test with $\eta^2$ effect size statistic. Differences in gender, education, annual income classes (K: 1000 Euro), percentages of participants with a search and rescue profession, traumatic events in the past (DIA-X: Diagnostic Expert System, traumatic event score), as well as pre-existing physical illness and pre-existing mental disorders diagnosed by a mental health professional between the mental health clusters were investigated in the entire cohort by $\chi^2$ test with Cramer’s *V* effect size statistic. *P* values were adjusted for multiple testing with the false discovery rate method. Age is presented in violin plots with single observations visualized as points and medians with interquartile ranges depicted by red diamonds and whiskers. The remaining variables are presented as percentages of the clusters in stack plots. Effect sizes and *P* values are displayed in the plot captions. Numbers of observations in the clusters are indicated in the *X* axes

**Fig. 5:**
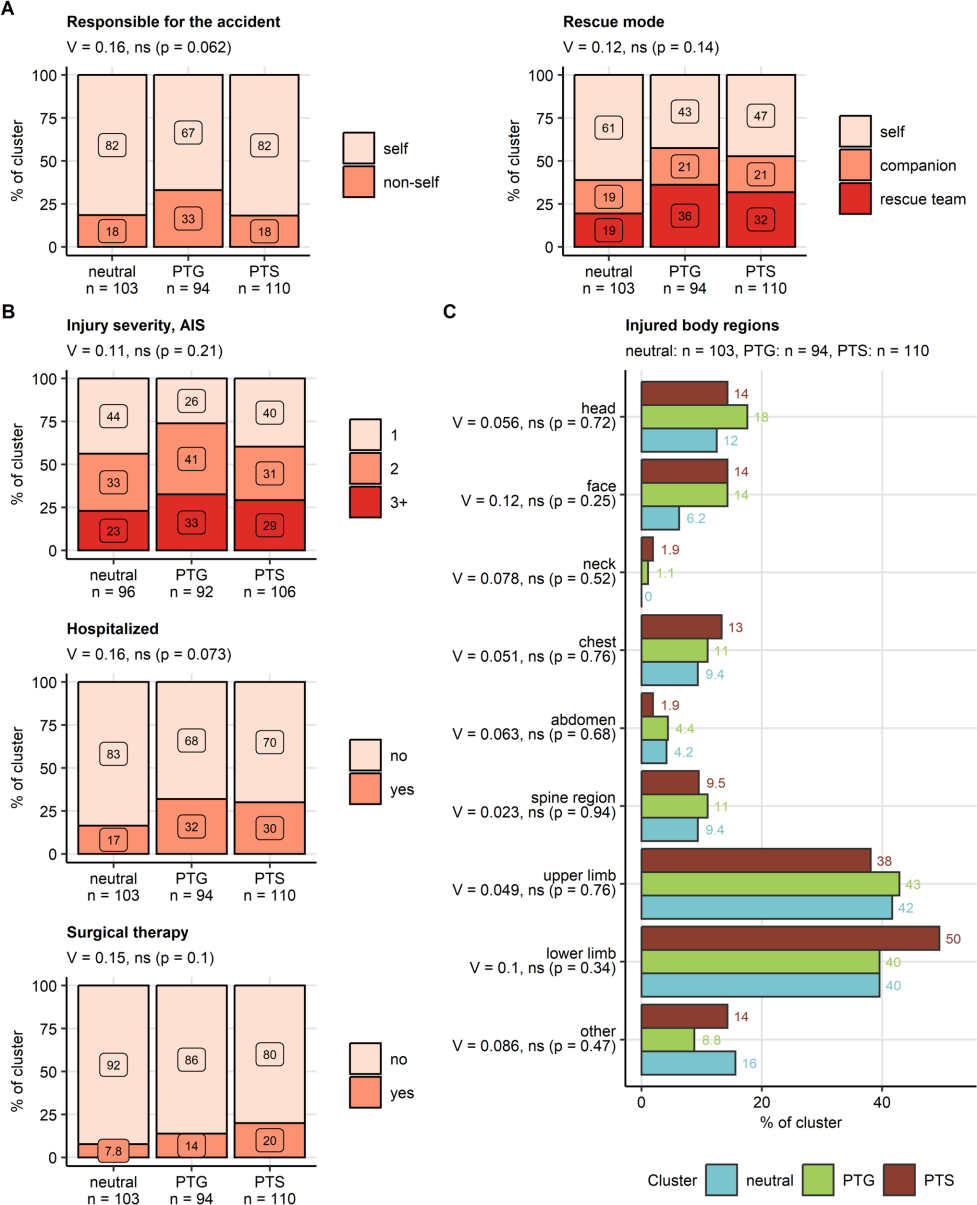
Responsibility for the accident, accident rescue, injury severity, and injured body parts in the mental health clusters. Differences in responsibility for the accident, rescue mode, injury severity, hospitalization and surgery rates, and injured body regions between the mental health clusters (neutral, post-traumatic growth [PTG], post-traumatic stress [PTS]) were investigated in the entire cohort by $\chi^2$ test with Cramer’s *V* effect size statistic. *P* values were adjusted for multiple testing with the false discovery rate method. **A**, **B** Accident responsibility, distribution of the rescue modes and injury severity classes (AIS: abbreviated injury scale), rates of hospitalization and surgery expressed as percentages of the mental health clusters were presented in stack plots. Effect sizes and *P* values are displayed in the plot captions. Numbers of observations in the clusters are indicated in the *X* axes. **C** Percentages of injured body regions were presented in a bar plot. Effect sizes and *P* values are indicated in the *Y* axis. Numbers of observations in the clusters are displayed in the plot captions

In more detail, individuals in the PTS cluster were the youngest of all participants (global difference between the clusters: *η*^2^ = 0.027, *p* = 0.023) and had the highest frequency of pre-existing diagnosed mental disorders (*V* = 0.28, *p* < 0.001). The frequency of pre-existing physical disorders was significantly higher in the PTG and PTS that in the neutral cluster (*V* = 0.19, *p* = 0.012). Gender, income, employment and education, frequency of search and rescue professionals, and prior traumatic events did not differ significantly between the clusters. We could not observe significant differences in frequency of being alone during the accident, or mode of rescue between the clusters. No significant differences in season in which the accident occurred, mountain sport type, and numbers of injured persons between the clusters were detected. Analogically, there were no significant differences in injury severity and location between the clusters (Figs. 4 and 5, Supplementary Figs. S12–S13, Supplementary Table S10).

**Fig. 6:**
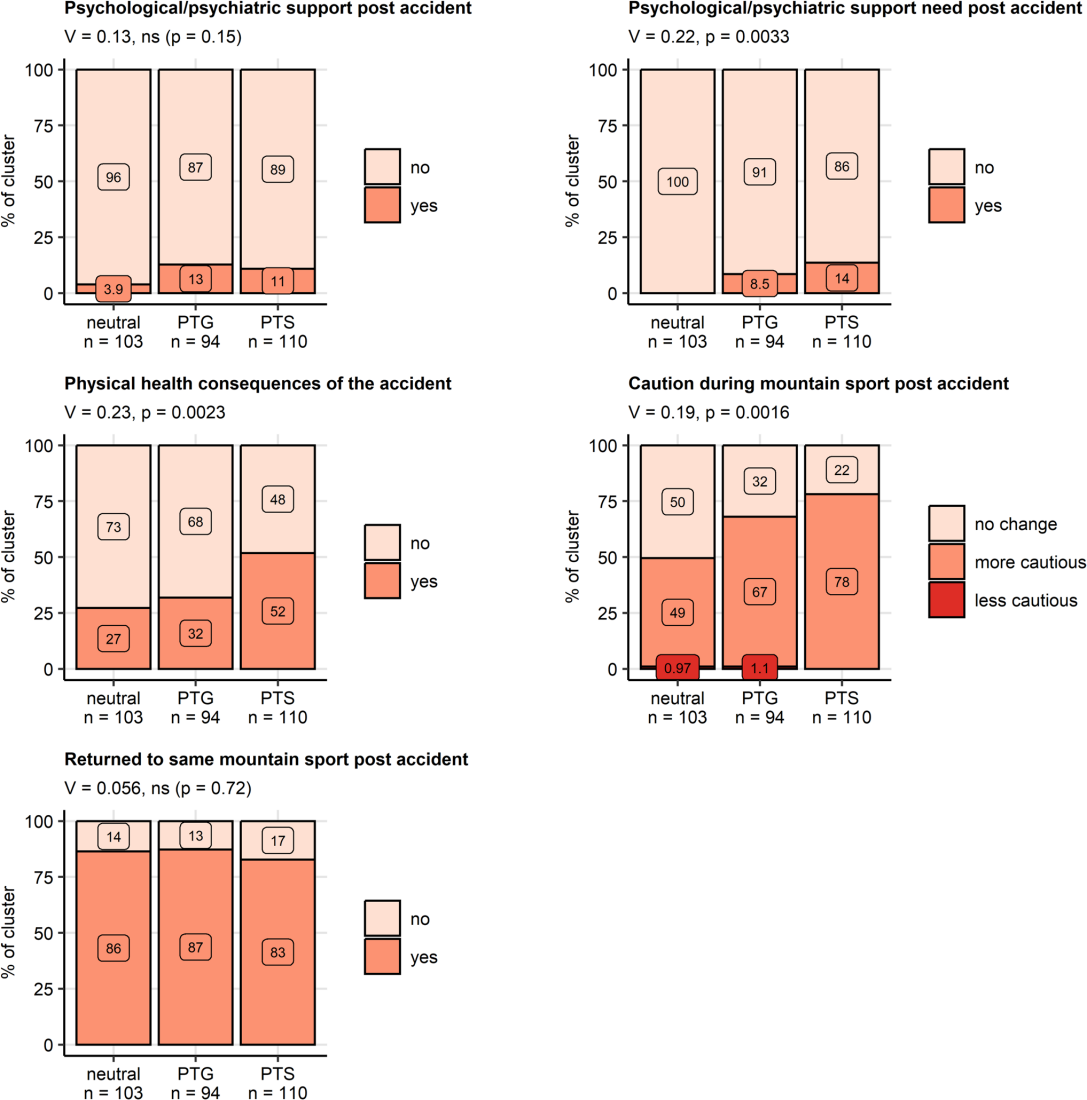
Psychological support and consequences of the accident in the mental health clusters. Differences in rates of received psychological or psychiatric support, self-reported need for psychological or psychiatric support after the accident, persistent physical health consequences of the accident, cautious behavior during mountain sport, and percentages of participants having returned to the same mountain sport between the mental health clusters (neutral, post-traumatic growth [PTG], post-traumatic stress [PTS]) were assessed in the entire cohort by $\chi^2$ test with Cramer *V* effect size statistic. *P* values were adjusted for multiple testing with the false discovery rate method. Percentages within the clusters are presented in stack plots. Effect sizes and *P* values are displayed in the plot captions. Numbers of observations in the clusters are indicated in the *X* axes

There were no significant differences in rates of participants who received psychological or psychiatric support after the accident between the clusters. Among PTG and PTS cluster individuals, 8.5% and 14%, respectively, reported a need for psychological or psychiatric support following the accident as compared with none in the neutral cluster (*V* = 0.22, *p* = 0.0033). The frequency of participants suffering from persistent physical health consequences of the accident was the highest in the PTS cluster (PTS: 52%, PTG: 32%, neutral: 27%, *V* = 0.23, *p* = 0.0023). Analogically, PTS cluster patients reported the highest rates of cautious behavior during mountain sport following the accident (PTS: 78%, PTG: 67%, neutral: 49%, *V* = 0.19, *p* = 0.0016). By contrast, percentages of participants who returned to the same mountain sport after the accident were comparable between the mental health clusters (Fig. 6).

### Prediction of the mental health cluster assignment by demographic, socioeconomic, and accident-related factors

Finally, we intended to model the mental health cluster assignment with demographic, socioeconomic, clinical, and accident-related factors available during acute medical management of the patient (Supplementary Table S11) with seven popular machine learning algorithms: the random forests, neural networks, support vector machines, recursive partitioning, discriminant analysis, elastic net regression, and conditional random forests (Supplementary Table S12). Such models would enable early identification of mountain sport accident victims at risk of mental health problems.

**Fig. 7:**
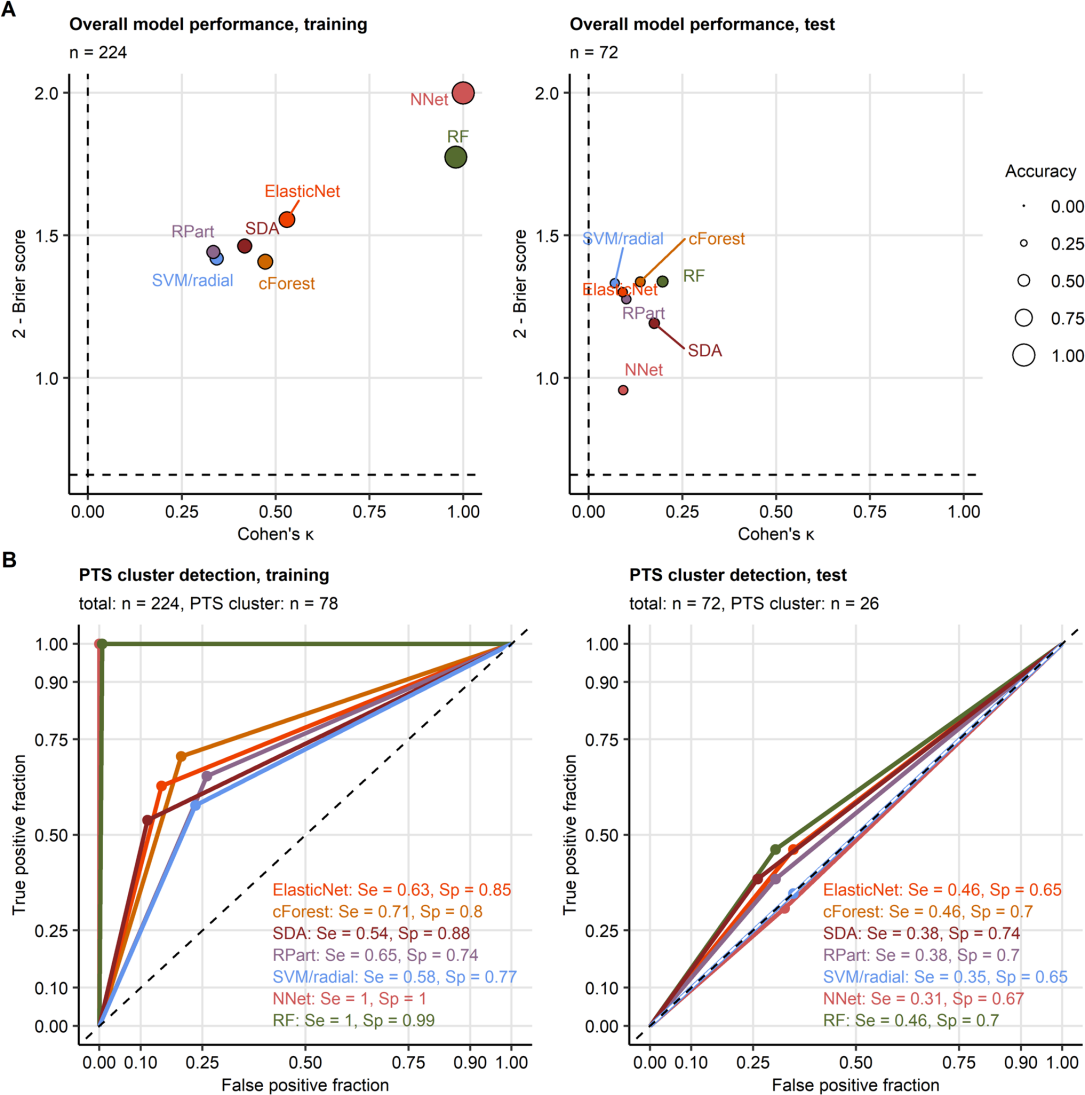
Assignment of accident victims to the mental health clusters based on explanatory factors available during acute medical management of the patient. The mental health cluster assignment was modeled with demographic, medical history, and accident-related explanatory factors available during acute medical management of the patient. Psychometric variables used for cluster definition, symptoms of mental disorders, resilience classes, presence and frequency of flashbacks, and variables concerning recovery and long-term consequences of the accident were excluded from the explanatory factor set. **A** Accuracy of the predicted cluster assignment and predictive performance of the modeling algorithms was assessed by overall cluster assignment accuracy, Cohen’s $\kappa$ inter-rater accuracy metric (high values indicate good accuracy), and Brier score (low values indicate good performance) in the training and test subsets of the study cohort. Performance metrics are presented in scatter plots. Point size codes for the overall cluster assignment accuracy. Point color codes for the modeling algorithm. Numbers of complete observations are displayed in the plot captions. **B** Sensitivity (Se) and specificity (Sp) of detection of participants assigned to the PTS cluster (post-traumatic stress) investigated by receiver-operating characteristic in the training and test subset of the study cohort. Sensitivity and specificity values are indicated in the plots. Line color codes for the modeling algorithm. Numbers of complete observations and observations in the PTS cluster are indicated in the plot captions. RF: random forest; NNet: neural network with a single hidden layer; SVM/radial: support vector machines with radial kernel; RPart: recursive partitioning; SDA: shrinkage discriminant analysis; cForest: conditional random forest; Elastic Net: elastic net multinomial regression

The cluster assignment models employing early predictors demonstrated a moderate-to-excellent prediction performance in the training subset of the study cohort (training accuracy: 56–100%, Cohen’s $\kappa$: 0.33–1, Brier score: 3e−04–0.59). However, their accuracy in cross-validation and in the test subset was poor (test accuracy: 38–46%, Cohen’s $\kappa$: 0.07–0.2, Brier score: 0.66–1). Accordingly, sensitivity of discrimination of the PTS cluster from the pooled neutral and PTG clusters was unsatisfactory in the cross-validation and test data (test, sensitivity: 0.31–0.46, Fig. 7, Supplementary Table S13). Pre-existing mental disorder or physical illness, hospitalization and surgery, injury severity, age, rescue mode, education, annual income, and traumatic events in the past were among the most important explanatory factors employed by these models (Supplementary Fig. S14). Of note, inclusion of follow-up-related variables such as received psychological or psychiatric support, the self-reported need for psychological or psychiatric support, physical health consequences, or caution during mountain sport activity in the machine learning models did not improve their accuracy at prediction of the mental health cluster assignment in cross-validation or the test subset (test accuracy: 36–46%, Cohen’s $\kappa$: 0.055–0.2, Brier score: 0.64–1.1, Supplementary Figs. S15–S16, Supplementary Table S14).

## Discussion

We characterized mental health outcomes in individuals treated at a tertiary trauma center for an accident in mountain sport. We identified three approximately equally sized subsets of participants with distinct mental health characteristic: (1) the neutral mental health cluster, (2) the PTG cluster characterized by a predominantly salutatory reaction to the accident manifested by post-traumatic growth, and (3) the PTS cluster hallmarked by symptoms of PTSD, anxiety, depression, and somatization as well as low resilience, quality of life, and sense of coherence. Individuals in the PTS cluster were characterized by younger age, the highest frequency of pre-existing diagnosed mental disorders, and persisting physical health consequences of the accident compared to individuals in the other two clusters. Even with robust machine learning algorithms we could not establish a reliable model to identify PTS cluster patients at risk of long-term mental health problems with routine clinical data available during acute medical management of the mountain sport accident victim.

This study evaluates a broad palette of mental health readouts including symptoms of anxiety and depression, somatization, resilience, sense of coherence and quality of life, post-traumatic growth, and symptoms of PTSD defined by positive screening of at least one PCL-5 domain in survivors of mountain sport accidents. This population is of special interest because, on one hand, mountain sports are generally considered beneficial for mental health [2, 3] but, on the other hand, bear accident and injury risks [5, 6]. Our study cohort was clearly not a representative sample of the alpine region population; nevertheless, we compared the analyzed sample with existing literature to put our results in a broader context of mental health accident survivors and mountain sports.

Rates of traumatic events measured by DIA-X tool [35] and of manifest PTSD defined as positive screening for all of the B, C, D, and E of the PCL-5 instrument [26] in our cohort were put into perspective by comparison with published estimates for the general population. It is important to note that in our study we specifically asked participants to rate symptoms of PTSD in relation to their mountain sport accident. In such analysis, the frequency of both traumatic events prior to the accidents and accident-related manifest PTSD in the cohort was significantly lower than in most multi-population surveys [51–54, 57]. Despite the substantial burden of mountain sport accidents on the medical system of alpine countries, with more than 13,000 recorded accidents in 2023 in Austria alone (this data set only comprises accidents recorded by the alpine police service [50]), not many studies assessed rates of PTSD and other mental disorders in survivors of mountain sport accidents. Survivors of avalanches reported long-term symptoms suggestive of PTSD at rates between 11 and 16% [23, 24]. Those estimates are comparable with 19% participants of our study screened positive for at least one of the PTSD domains defined by the PCL-5 tool [26]. PTSD frequencies of 11.8–26% have been reported at 3–12 months after acute trauma [11, 13, 15]. However, in our cohort, solely 1.3% of participants demonstrated concomitant PCL-5 domain B, C, D, and E symptoms suggestive of fully manifest PTSD.

Our study did not specifically address professional mountain rescuers or mountain sport professionals, and only 7.2% of the sample reported a search and rescue profession and 5.2% a mountain sport profession. Studies, conducted on mountain guides and mountain rescue personnel, showed that 71–78% of them experienced a traumatic event, while the reported frequency of PTSD was low (0.9–2.7%) [21, 25]. This is comparable to estimates of traumatic events and manifest PTSD in the general population [52–54, 57] and, particularly, lower than in other potentially vulnerable groups such as emergency workers [58]. By contrast, a recent French study found PTSD in up to 22% of mountain rescuers once differentiating between clinical interviews and self-reporting of symptoms for the assessment of PTSD [22]. Resilience has been proposed as a protective trait against negative mental health consequences following trauma [59]. Two-thirds of our study participants were classified as highly resilient [31]. Of note, both individuals, the study cohort and the cohort of mountain rescuer by Mikutta et al. displayed substantially higher levels of resilience as compared with the general population [21, 31, 55], which can explain low prevalence of manifest PTSD in individuals performing mountain sports in a free time or professional setting. It is likely that this high resilience is attributed to the protective effect of physical activity in an alpine environment [2]. The high resilience scores may also explain lower frequencies of clinically relevant symptoms of anxiety and depression in our cohort compared to the general Austrian population at the time of our survey [34]. The high resilience in the study cohort may also pertain to the rating of quality of life which was significantly higher than in the general western European population [33].

To our best knowledge, our study provides the first multi-faceted characteristic of long-term consequences of a mountain sport accidents for mental health. By semi-supervised clustering, we identified mountain sport accident victims with co-existing symptoms of PTSD, depression, anxiety, somatization, as well as low sense of coherence and quality of life. Those individuals assigned to the PTS subset may represent a clinically relevant risk group of post-traumatic mental disorders. The psychometric characteristic of the PTS cluster corresponds to the multi-faceted mental symptoms described for individuals following a traumatic event [8, 60]. Regarding early predictors of mental health problems in mountain accident victims, the PTS cluster was characterized by a significantly younger age as well as high rates of pre-existing physical and mental disorders. Of note, female gender [15], young age at traumatization [13], low income [61], and pre-existing mental disorders [16] have been proposed as risk factors of PTSD. PTSD risk has also been previously associated with injury severity, head injuries, hospitalization length, and pain [62–64]. In our study, we could not establish any link between the mental health clusters and, in particular, the PTS cluster with objective injury severity measured with the AIS scale [37] or injury location. Intriguingly, subjective injury severity, which was not surveyed in our cohort, was shown to be a stronger predictor of PTSD symptoms than objective injury severity [65]. Importantly, the percentage of participants having received psychological or psychiatric support after the accident was generally low in the study cohort (9.1%) and did not differ between the mental health clusters. Hence, it is unlikely that psychological or psychiatric intervention had any impact on classification of the participants.

In our hands, none of seven machine learning procedures using different mathematical principles, random forest, neural network, support vector machines, recursive partitioning, conditional random forest, discriminant analysis, or elastic net regression, was able to reliably predict the mental health cluster assignment based on 40 demographic, socioeconomic, clinical, and accident-related variables available during acute routine medical treatment of the patient. This phenomenon is likely attributed to the lacking strong non-psychometric explanatory variables differentiating between the mental health clusters. Early identification of trauma patients at risk of developing PTSD or further mental disorders has previously been tackled by diverse machine learning algorithms [12, 13, 16, 64]. Interestingly, inclusion of peri-traumatic mental symptoms like PTSD features, flashbacks, hyperarousal, or subjective need for support was shown to be crucial for optimal prediction of long-term mental disorders or PTSD during recovery [12, 13, 15, 16, 64]. Unfortunately, such variables were not available in our cohort investigated at least 6 months after the accident. Our findings are in line with a recent initiative aiming to improve mental health following accidents using peri-traumatic screening tools as well as including mental health support in the aftercare of trauma patients [66].

### Limitations

Our study bears limitations. Our cohort is not a representative sample of the general population. The overall analysis inclusion rate was low and, as indicated by comparison of the excluded and analyzed participants, the results were potentially affected by a selection bias toward individuals with higher social status and more severe injuries. Our comparison of demographic and accident-related features in the study cohort, Austrian population, and the national registry of alpine accidents indicates that the analyzed collective is a sample of the general physically active population. Low response rates are unfortunately a problem of many studies tackling PTSD and mental disorders [67]. The sensitivity analysis showed that individuals with incomplete mental health data were preferentially assigned to the PTS cluster. This suggests mental distress or disorders as one of the reasons for not completing of the study survey. Additionally, this may indicate that symptoms of mental health disorders are under-reported in the study cohort.

We cannot exclude the possibility that results were affected by other events than the accident of interest. This was in part addressed by the design of the survey questions which pointed to the mountain sport accident. As assessed by sensitivity analysis, our clustering structure was hardly affected by the time of between the accident and survey or prior mountain sport accidents. However, to finally clarify the issue of the accident specificity of mental health alterations, follow-up studies with longitudinal design or a control group are required. Furthermore, the study survey did not record potentially important explanatory factors for distinction between the mental health clusters such as need for rehabilitation or ability to work. Due to the cross-sectional design of the study, we were not able to assess peri-traumatic mental health, which has been shown to be crucial for identification of vulnerable patients [12, 13, 15, 16]. This analysis only included individuals who were treated for a mountain sport accident at the local trauma department. Individuals without contact to the medical system or bystanders, who can also be affected by PTSD, were not examined. Finally, as in many studies performed in recent years, a potential impact of the COVID-19 pandemic on results is possible.

## Conclusions and outlook

Our study characterizes symptoms of PTSD and other facets of mental health following mountain sport accidents. We identified symptoms of mental health impairment in roughly one-third of the investigated individuals. This finding is significant because people performing mountain sports are generally regarded as robust in terms of physical and mental health. However, despite high levels of resilience, these individuals are not free of risk of symptoms of PTSD, manifest PTSD, and other mental disorders. Destigmatization of such mental health problems is essential in the mountaineering community to allow for timely support.

In the current analysis, we could not identify robust predictors of impaired mental health at follow-up among variables available during early routine medical treatment. Hence, until standardized robust predictor variables are available and screening protocols assessing mental health outcomes are widely implemented, low-threshold access to mental health support as well as psychoeducational measures seems the most appropriate strategy for successful interdisciplinary management of individuals following accidents in mountain sport.

### Supplementary Information

Below is the link to the electronic supplementary material.

Supplementary file 1 (DOCX 5107 kb)

Supplementary file 2 (XLSX 37 kb)

## Data Availability

Anonymized patient data will be made available upon request to the corresponding author. The study analysis pipeline is available at https://github.com/PiotrTymoszuk/mental_accident.
