# Supplementary material for: Three distinct patterns of mental health response following accidents in mountain sports: a follow-up study of individuals treated at a tertiary trauma center
Source: Eur Arch Psychiatry Clin Neurosci. 2024 May 10;274(6):1289–310. doi: 10.1007/s00406-024-01807-x (PMC11362256; doi:10.1007/s00406-024-01807-x)
Supplement: Supplementary file 1 — (DOCX 5107 kb) [file 406_2024_1807_MOESM1_ESM.docx]

Three distinct patterns of mental health response following accidents in mountain sports: a follow-up study of individuals treated at a tertiary trauma center

Supplementary Material

# Supplementary Methods

## Software

Data analysis and transformation was accomplished with R version 4.2.3. The study data set was imported from an SPSS file with the *foreign* package (1). Tabular data were handled with the *tidyverse* package bundle (2), and the packages *rlang* (3) and [*trafo*](https://github.com/PiotrTymoszuk/trafo). Text variables were handled with *stringi* (4). Imputation of missing values of psychometric variables was done with the package *impute* (5).

For distribution testing (normality, variance, Gini index), the packages [*ExDA*](https://github.com/PiotrTymoszuk/ExDA), *rstatix* (6) and *DescTools* (7) were utilized. Consistency of psychometric tools was assessed with the packages *psych* (8), [*ExDA*](https://github.com/PiotrTymoszuk/ExDA) and [*clustTools*](https://github.com/PiotrTymoszuk/clustTools). Clustering tendency was investigated with *factoextra* (9) and [*clustTools*](https://github.com/PiotrTymoszuk/clustTools). Statistical hypothesis testing and correlation analyses were accomplished with [*ExDA*](https://github.com/PiotrTymoszuk/ExDA) and *rstatix* (6). Bootstrapped estimates of prevalence of mental disorder symptoms and traumatic events with their 95% confidence intervals were computed with the development package [*bootStat*](https://github.com/PiotrTymoszuk/bootStat).

For semi-supervised clustering, diagnostic and performance testing of the clustering analysis, the package [*clustTools*](https://github.com/PiotrTymoszuk/clustTools) with implementation of algorithms and distance measures from *factoextra*, *cluster* and *philentropy* (9–11) was utilized. UMAP (uniform manifold approximation and projection) was done with the *umap* and [*clustTools*](https://github.com/PiotrTymoszuk/clustTools) packages (12,13). Cross-distances between the training and test subsets of the study cohort and cross-distances between clusters in the training and test subsets of the study cohort were computed with *philentropy* (11) and [*clustTools*](https://github.com/PiotrTymoszuk/clustTools).

Multi-variable classifiers of cluster assignment were developed with the following algorithms: random forests (14,15), neural network (16), support vector machines with radial kernel (17,18), recursive partitioning (19,20), conditional random forests (21–23), shrinkage discriminant analysis (24,25), and elastic net multinomial regression (26,27). For tuning, training, prediction and assessment of performance of the classifiers, the packages *caret* (28) and [*caretExtra*](https://github.com/PiotrTymoszuk/caretExtra) were used.

Results were visualized with *ggplot* (bar plots, box plots, heat maps of cross-distances, scatter plots) (29), *plotroc* (30), [*ExDA*](https://github.com/PiotrTymoszuk/ExDA) (violin, stack and ribbon plots) and [*clustTools*](https://github.com/PiotrTymoszuk/clustTools) (cluster quality control plots, heat maps of clustering features, distance heat maps, scatter plots of UMAP layouts) and *ComplexUpset* (visualization of overlap with upset plots) (31). Figures were created with the packages *cowplot* (32) and *patchwork* (33). Tables were generated with *flextable* (34). The manuscript and Supplementary Material were written in the *rmarkdown* environment (35) with the package *bookdown* (36). Figures, tables and R expressions in the markdown documents were managed with the development package [*figur*](https://github.com/PiotrTymoszuk/figur). The markdown documents were rendered with the *knitr* (37) and *bookdown* (36) packages and with [author-info-blocks.lua](https://github.com/pandoc/lua-filters/blob/master/author-info-blocks/author-info-blocks.lua) and [scholarly-metadata.lua](https://github.com/pandoc/lua-filters/blob/master/scholarly-metadata/scholarly-metadata.lua) scripts by Albert Krewinkel and Jörn Krenzer.

## Data import and transformation

The study data set was imported from an SPSS file with raw study data (function read.spss(), package *foreign*). The list of extracted variables with their description is available in **Supplementary Table S1**. A total of 307 participants with the complete set of psychometric battery variables (**Supplementary Table S2**) were included in the analysis (**Figure 1**).

The psychometric battery (**Supplementary Table S2**) consisted of German versions of assessment tools for anxiety (GAD-7: 7-item general anxiety disorder scale) (38), depression (PHQ: patient health questionnaire, PHQ-9 and PHQ-8) (39–41), panic (PHQ-panic module) (39,40), common persistent somatic symptoms as a substitute for somatization (PHQ-15) (42), resilience (RS13: 13-item resilience scale) (43), loss of sense of coherence (SOC-9L: Leipzig 9-item sense of coherence questionnaire) (44), quality of life (EUROHIS-QOL 8: 8-item EUROHIS project quality of life scale) (45), post-traumatic growth (PTGI: post-traumatic growth inventory) (46) and post-traumatic stress disorder (PCL-5: PTSD checklist for DSM-5) (47). In the study survey, items of the PTGI and PTSD instruments, and questions concerning flashbacks were provided with a captions indicating that the questions refer to the mountain sport accident of interest (‘The following questions refer to consequences of your accident’).

Clinically relevant symptoms of anxiety were defined as GAD-7 $\geq$ 10, clinically relevant symptoms of depression were defined as PHQ-9 $\geq$ 10 (48), significant persistent somatic symptoms were defined as PHQ-15 $\geq$ 10 (42). Resilience classes were defined as follows: low: RS-13 0 - 65, moderate: 66 - 72, high: $\geq$ 73 (43). For a comparison of frequency and severity of depressive symptoms in the study cohort with microcensus data for the general Austrian population, mild depressive symptoms were defined as 5 - 9, moderate symptoms as 10 - 14, moderately severe symptoms as 15 - 19, and severe symptoms as $\geq$ 20 points of the PHQ-8 score (41).

Items of the EUROHIS QOL 8 tool were scored as with 1 - 5 Likert scales (1: extreme concerns, no satisfaction at all, 5: no concerns/full satisfaction) with each item representing a single domain of quality of life (quality of life, health, energy, finances, activity, self-esteem, relationship and housing). The total EUROHIS QOL mean score was defined as the arithmetic mean of all items (45).

Separate scores were computed for each domain of the PTGI tool (I: relations, II: possibilities, III: personal strength, IV: spiritual strength, V: life appreciation) with each item scores as 0: none, 1: very little, 2: little, 3: moderate, 4: great, 5: extremely great. In addition, the total PTGI score was calculated as the sum of all items (46).

Separate scores were calculated for domains B, C, D and E of the PCL-5 tool along with the total score being the sum of all items. Each PCL-5 item was scored as 0: not at all, 1: a little bit, 2: moderate, 3: quite a bit and 4: extremely. Participants positive for the B domain or C domain PTSD symptoms were identified by at least one item per domain scored with ‘moderate’ or higher. Participants positive for the D or E domain PTSD symptoms were identified by at least two items per domain scored with ‘moderate’ or higher. Significant PTSD symptoms were assumed in participants screened positive for at least one of the B, C, D or E PCL-5 domains. Manifest PTSD was considered for participants positive for all four PCL-5 domains (47).

Traumatic events prior to the mountain sport accident were assessed with the DIA-X tool (Diagnostic Expert System) (49). Direct personal experience or being a witness of a traumatic event specified by the DIA-X questionnaire or by an additional yes/no item (‘other traumatic events’) was scored as 1. Prior traumatic event was assumed with at least one DIA-X item scored with 1. Pre-existing mental disorder diagnosed by a physician, prior mountain sport accidents, flashbacks of the surveyed accident during mountain sport activity, confusion during mountain sport activity, self-reported need for psychological support following the accident, psychological/psychiatric support/therapy after the accident and presence of persistent physical health consequences related to the accident were surveyed as single yes/no items. Flashbacks frequency during mountain sport activity was assessed in the following categories: none, more than one per year and more than one per month. Smoking was surveyed as a single yes/no question. Alcohol use was investigated with the CAGE tool with $\geq$ 2 points indicative of problematic alcohol consumption (50). Data on the type of the accident date and daytime, accident mountain sport, injury diagnosis, injured body regions, injury severity, hospital treatment, surgery and number of ICD-10 surgical diagnoses were extracted from electronic patient’s records. Injury severity was assessed with the abbreviated injury scale (AIS) (51).

Accident sport types were classified in accordance with the annual accident report of the Austrian Board of Mountain Safety (52) as follows: *alpine skiing/snowboarding* (all types of alpine ski and snowboard sports in secured areas of ski resorts: pistes, secured slopes, lifts and cable cars), *ski touring/freeride* (all types of alpine ski and snowboard sports performed in non-secured areas of ski reports, e.g. ski touring, freeride), *cross-country skiing*, *sledding* (all types of sledding sports such as sledding and bob-sledding), *ice climbing* (ice and mixed climbing in the winter season), *hiking*, *climbing* (sport and rock climbing, bouldering), *mountaineering* (high mountain and glacier tours), *biking* (all types of biking including road, cross-country and mountain biking), *air sport* (only paragliding present in our cohort), *water sport*, and *other* (non-classifiable sport types such as ski jumping). Alpine skiing/snowboarding, ski touring/freeride, cross-country skiing, sledding, and ice climbing were classified as winter sports. Hiking, climbing, mountaineering, biking, air sport, and water sport were classified as summer sports. Additional information on study variables and their stratification schemes are presented in **Supplementary Table S1**.

Information on genders ($\geq$ 18 years, in 2023) (53), age ($\geq$ 18 years, in 2023) (53), education (15 - 64 years old, in 2020) (54) and employment structure ($\geq$ 15 years, in 2021) (55), smoking status ($\geq$ 15 years, in 2019) (56), and self-reported chronic physical or mental illness ($\geq$ 15 years, in 2019) (57) for the Austrian population were extracted from publications of Statistik Austria, the national statistical bureau.

Counts of mountain accident victims in 2023 in Austria stratified by mountain sport type, age class and month of the accident were extracted from the annual report of the Austrian Board of Mountain Safety (52). Of note, we were not able to consistently eliminate deaths (n = 266), accidents with tourists from non-German-speaking countries, non-adult victims (nation-wide, $\geq$ 20 years old: n = 2570) and non-sportive accidents (n = 648) from this published nation-wide data set. Yet these subsets of mountain accident victims constituted a minority of all nation-wide mountain accidents in 2023 (n = 13681).

Lifetime estimates of prevalence of traumatic events and PTSD in the general population were obtained from representative survey studies (58–62). Frequency of traumatic events, manifest PTSD and PTSD symptoms subsumed under the B, C, D, and E domains of the PCL-5 instrument in Swiss mountain rescuers were extracted from the report by Mikutta et al. (63). Data on frequency of clinically relevant anxious (GAD-7 $\geq$ 10) and depressive (PHQ-9 $\geq$ 10) symptoms in Austria in 2022 were obtained from a publication by Humer et al. (48). Mean scores of resilient comping with their standard deviations (SD) obtained with the RS-13 tool were extracted from reports with samples of the German (43) and Tyrolean (64) populations, and from a cohort of Swiss mountain rescuers (63).

## Consistency of psychometric tools and power analysis

Consistency of psychometric tools was assessed by factor analysis and McDonald’s $\omega$ (function omega(), package *psych*) (8,65). The number of latent factors for calculation of $\omega$ was identified by inspection of loadings determined by factor analysis (function reduce_data(), [*clustTools*](https://github.com/PiotrTymoszuk/clustTools)) (66). All psychometric tools used in the study except for the stress PSS-4 scale (67) and the BRCS resilience tool (68) exhibited good-to-excellent consistency with $\omega$ > 0.8 (**Supplementary Table S3**). The poorly performing PSS-4 and BRCS scales were excluded from the further analysis.

To find the optimal size of a training subset of the study cohort, random subsamples of the study data set of varying sizes were investigated (50, 100, 150, 200, 250, 300 observations, 50 random draws per subsample size). For each random subsample, clustering tendency was assessed by Hopkins statistic (function get_clust_tendency(), package [*clustTools*](https://github.com/PiotrTymoszuk/clustTools)) (69). For n = 250 observations, the median Hopkins statistic was 0.73 (interquartile range: 0.73 - 0.74) indicative of good clustering tendency. The Hopkins statistic value for this subset was comparable with the value for the entire subset (0.74). For these reasons, 3/4 of the entire data set or n = 230 was considered as the adequate size of the training subset of the study cohort for a reproducible clustering analysis.

## Training/test subset definition

The study participants were assigned to the training and test subsets with the 3:1 size ratio (**Figure 1**). To this end, 100 random splits of the data sets were generated. The subset assignment scheme was chosen with the possibly smallest differences in sociodemographic, medical history, clinical and accident- and injury-related variables assessed by Gower distance between the training and test subsets (function dist(), package *proxy*) (70).

## Statistical hypothesis testing, effect size and multiple testing correction

Differences in numeric variables were assessed with Mann-Whitney test with r effect size statistic or Kruskal-Wallis test with $\eta^{2}$ effect size statistic for two and more than two analysis groups, respectively. Differences in frequency of categories of qualitative variables between analysis groups were investigated with $\chi^{2}$ test with Cramer’s V effect size statistic. P values were corrected for multiple testing with the false discovery rate method separately for each analysis task (e.g. comparison of clusters) (71). Effects with p < 0.05 following the false discovery rate adjustment were considered significant. Effect size of accuracy of predicted cluster assignment by machine learning classifiers was assessed by Cohen’s $\kappa$ inter-rater reliability statistic (72,73). Intervals of effect sizes were defined as follows (73–75):

- r statistic: small: < 0.3, moderate: 0.3 - 0.5, large: $\geq$ 0.5
- Cramer’s V statistic: small: < 0.3, moderate: 0.3 - 0.5, large: $\geq$ 0.5
- $\eta^{2}$ statistic: small: < 0.06, moderate: 0.06 - 0.14, large: $\geq$ 0.14
- Cohen’s $\kappa$ inter-rater reliability statistic: none: < 0.2, minimal: 0.2 - 0.4, small: 0.4 - 0.6, moderate: 0.6 - 0.8, large: $\geq$ 0.8

## Assessment of selection bias

To assess the possible selection bias, demographic, socioeconomic, clinical, accident- and recovery-related parameters were compared between individuals excluded due to missingness of psychometric data or denying survey participation and participants included in the analysis (**Figure 1**). Categorical variables were compared by $\chi^{2}$ test with Cramer V effect size statistic and numeric variables were compared by Mann-Whitney test with r effect size statistic (function compare_variables(), package [*ExDA*](https://github.com/PiotrTymoszuk/ExDA)). Potential differences between the training and test subset of the study cohort (**Figure 1**) were analyzed in an analogical way. Significant (p < 0.05) differences between the included/excluded participants are presented in **Supplementary Table S4** and **S5**.

## Comparison of socioeconomic, accident and mental health features with population estimates and literature reports

Differences in age, gender, education, professional activity, and frequency of chronic physical and mental disorders between the study cohort and the Austrian population (53,53–56) were investigated by Mann-Whitney U test with r effect size statistic (age as a numeric variable) or $\chi^{2}$ test with Cramer’s V effect size statistic (remaining categorical variables). Global differences in distribution of mountain sport types, age and accident months between the analyzed cohort and the nation-wide collective of Austrian mountain accident victims (52) were compared with $\chi^{2}$ test with Cramer’s V effect size statistic. In the comparison of sport types, nation-wide counts of non-sportive mountain accidents (e.g. during forestry or hunting) were excluded. In the comparison of age distributions, only individuals older than 20 years were included. Post-hoc comparisons of the study cohort and nation-wide Austrian counts were done with Holm-corrected $\chi^{2}$ test. The statistical hypothesis testing was done with the function compare_variables() from the [*ExDA*](https://github.com/PiotrTymoszuk/ExDA) package. The comparison results are presented in **Supplementary Table S6** and **Supplementary Figure S1**.

Frequency of traumatic events, manifest PTSD and PTSD symptoms in the current cohort was compared with original reports providing estimates of frequency of traumatic events and PTSD in the general population (58–62) and in a cohort of Swiss mountain rescuers (63). Frequencies of clinically relevant symptoms of anxiety and depression measured, respectively, with GAD-7 and PHQ-9, were compared with a representative sample of the general Austrian population reported by Humer et al. (48). Frequency and severity of depressive symptoms measured by the PHQ-8 tool in the study cohort (41) were compared with the Austrian 2019 microcensus estimates (57). In those analyses, bootstrap with B = 1000 resamples was used for computation of the bias-corrected and accelerated (BCA) 95% confidence intervals (function bmap(), package [*bootStat*](https://github.com/PiotrTymoszuk/bootStat)). Significant differences were assumed for non-overlapping 95% confidence intervals of the mental symptom frequency estimates.

## Semi-supervised clustering

Observations of the training subset of the study cohort were subjected to clustering in respect to the numeric psychometric scores (**Supplementary Table S2**). The score values were normalized and median-centered (function center_data(), package [*clustTools*](https://github.com/PiotrTymoszuk/clustTools)). For clustering, the PAM (partition around medoids) algorithm (10) with the cosine distance measure between observations (11) was employed. The clustering object was constructed with the function kcluster() from the [*clustTools*](https://github.com/PiotrTymoszuk/clustTools) package. The choice of the clustering algorithm was motivated by its good explanatory performance measured by the fraction of explained clustering variance (ratio of the total between-cluster sum of squares to the total sum of squares), good separability between the clusters measured by mean silhouette width (76), and superior reproducibility in 10-fold cross-validation (77) (cluster assignment in the folds by an inverse distance weighted 27-nearest neighbors classifier) in a comparison with several other clustering algorithms presented in **Supplementary Figure S6A**. The explained variance, mean silhouette width and cross-validated accuracy statistics were computed with the methods summary() and cv() from the [*clustTools*](https://github.com/PiotrTymoszuk/clustTools) package. The number of clusters was chosen based on the bend of the curve of within-cluster sum of squares and the peak of mean silhouette statistic (method plot(), package [*ExDA*](https://github.com/PiotrTymoszuk/ExDA)) (9,76). By this means, three mental health clusters were defined: the neutral, PTG (post-traumatic growth) and PTS (post-traumatic stress) cluster (**Supplementary Figure S6B**).

Assignment of the training subset observations to the mental health clusters was accomplished with an inverse distance weighted 27-nearest neighbor classifier. Comparably good separation of the clusters could be discerned in the training and test subsets by a visual analysis of UMAP layouts and pairwise distance heat maps (**Supplementary Figure S7**). Additionally, the reproducibility of the clustering structure was assessed by mean silhouette width as a measure of general cluster separation (training: 0.3, test: 0.26) (76), fraction of observations with negative silhouette widths as a metric of misclassification rate (training: 0.043, test: 0.078) (76), fraction of explained clustering variance defined as ratio of the total between-cluster sum of squares to the total sum of squares (training: 0.55, test: 0.52), and fraction of 5-nearest neighbors placed in the same cluster as a measure of neighborhood preservation (training: 0.89, test: 0.8) (78). These statistics were computed with the summary() method from the [*clustTools*](https://github.com/PiotrTymoszuk/clustTools) package. The results of the comparison of the numeric statistics are presented in **Supplementary Figure S8A**. Distribution of the cluster frequency was similar in the training and test subset (**Supplementary Figure S8B**). An analysis of cosine cross-distances between the clusters in the training and test subsets revealed far higher similarity of the corresponding mental health clusters (i.e. neutral vs neutral, PTG vs PTG, PTS vs PTS) as compared with similarity of non-analogous clusters (e.g. neutral vs PTG) (distances computed with the function cross_distance(), package [*clustTools*](https://github.com/PiotrTymoszuk/clustTools); **Supplementary Figure S8C**). Finally, quality of semi-supervised clustering was investigated by comparison of normalized levels of the clustering variables between the mental health clusters in the training and test subset by Kruskal-Wallis test with $\eta^{2}$ effect size statistic (function compare_variables(), package [*ExDA*](https://github.com/PiotrTymoszuk/ExDA); **Figure 2**, **Supplementary Figure S9**, **Supplementary Table S8**).

Differences in frequencies of mental disorder symptoms as well as demographic, socioeconomic, clinical, accident- and recovery-related factors between the mental health clusters were assessed in the entire cohort by $\chi^{2}$ test with Cramer V effect size statistic and by Kruskal-Wallis test with $\eta^{2}$ effect size statistic for categorical and numeric variables, respectively (function compare_variables(), package [*ExDA*](https://github.com/PiotrTymoszuk/ExDA); **Supplementary Table S9** and **S10**).

## Assessment of effects of missing observations, annual income, prior mountain sport accidents, injury severity, hospitalization, and accident year on mental health cluster definition

To investigate effects observations excluded from the analysis due to an incomplete psychometric data set on definition of the mental health clusters, the excluded observations with less than 80% of missing psychometric entries were subjected to imputation with the 9-nearest neighbor algorithm implemented by the impute.knn() function from the R package *impute* (5). Subsequently, the mental health clusters were developed for the analysis cohort and for the analysis cohort appended with the imputed observations. Analogically, to explore effects of prior mountain sport accidents, the mental health clusters were developed for the entire cohort, the individuals having experienced a mountain accident in the past, and participants without prior mountain sport accidents. To evaluate effects of annual household income, the mental clusters were developed for the analysis cohort and the analysis cohort without, respectively, participants with no income, yearly income < 30000 Euro, yearly income ranging from 30000 to 45000 Euro and yearly income > 45000 Euro. To assess the impact of injury severity, the mental clusters were developed for the analysis cohort and the analysis cohort depleted of, respectively, individuals with mild (AIS 1), moderate (AIS 2), and severe-to-critical injuries (AIS $\geq$ 3). To investigate the effect of hospitalization, the mental health clusters were developed separately for the analysis cohort, non-hospitalized and hospitalized participants. To assess the impact of accident year, the mental clusters were developed for the analysis cohort without, respectively, accidents in 2018, 2019 and 2022. In each case, the unsupervised PAM/cosine distance clustering algorithm was used, i.e. the procedure employed for definition of the genuine mental health clusters. The effects of extending or depletion of the data set on the quality of the clustering structure was evaluated by comparing fractions of the explained clustering variance between the clustering solutions for the particular data set and for the analysis cohort (79). Additionally, sizes of the neutral, PTG and PTS clusters expressed as percentage of all clustered observations were compared (**Supplementary Figure S10** and **S11**).

## Cluster assignment classifiers

Two types of multi-parameter machine learning classifiers of the mental health cluster assignment were developed in the training subset:

1. models employing candidate early predictors of cluster assignment, i.e. demographic, socioeconomic, medical history and accident-related explanatory factors available during acute medical management of the accident victim
2. models including additionally recovery-related predictors such as persistent physical health consequences, flashbacks or cautious behavior during sport

Of note, psychometric variables used for definition of the mental health clusters as well as symptoms of mental disorders, presence and frequency of flashbacks were excluded from the explanatory variable sets. The explanatory variables are listed in **Supplementary Table S11**.

The models employed the following algorithms: canonical random forests (14,15), regularized neural networks with a single hidden layer (16), support vector machines with radial kernel (17,18), recursive partitioning (19,20), shrinkage discriminant analysis (24,25), conditional random forest (21–23), and elastic net multinomial regression (26,27). The optimal values of the algorithms’ parameters were found by 10-fold cross-validation-based tuning with the maximal value of Cohen’s $\kappa$ (72) as the tuning criterion. The tuning and fitting (‘training’) in the training subset of the study cohort was done with the wrapper function train() provided by the package *caret* (28). For the random forest and conditional random forest algorithms, 1000 random trees each were constructed. Test statistic, p values, number of splits and other parameters of the conditional forest models were controlled with the ForestControl object returned by the convenience wrapper cforest_unbiased() provided by the *party* package (23). The optimal algorithm parameter sets are listed in **Supplementary Table S12**. Predictions of the cluster assignment in the test subset of the study cohort were obtained with the predict() method from the [*caretExtra*](https://github.com/PiotrTymoszuk/caretExtra) package.

Overall accuracy and Cohen’s $\kappa$ statistics (72) were computed with the summary() method from the [*caretExtra*](https://github.com/PiotrTymoszuk/caretExtra) package. Brier scores (80) were computed with the following formula:

$$BS=\frac{1}{N}\times\Sigma_{i=1}^{N}\Sigma_{c=1}^{R}\left( f_{ic}-o_{ic} \right)^{2}$$

where $f_{tc}$ is the output probability of assignment of the i-th observation to the c-th cluster, $o_{ic}$ is the numeric-coded actual assignment of the i-th observation to the c-th cluster, $R$ is the total cluster number and $N$ is the total observation number.

Performance statistics for the training subset, 10-fold cross-validation and test subset of the study cohort are listed in **Supplementary Table S13** and **Supplementary Table S14** for the classifiers employing the early and full predictor set, respectively.

Variable importance statistic specific for the machine learning algorithm were extracted from the *caret* models with the varImp() function from package *caret* (28). They were: permutation importance for the random forest and conditional forest algorithm (14,23), connection weight importance statistic for the neural network (81), sum reduction in classification error attributed to each variable at each split for recursive partitioning (20), and linear model coefficient $\beta$ for elastic net regression (26). For support vector machines and discriminant analysis, the variable importance was computed based on area under the ROC curve for single predictors for discrimination between the cluster pairs (28).

## Data and code availability

Anonymized patient data will be made available upon request to the corresponding author. The study analysis pipeline is available at <https://github.com/PiotrTymoszuk/mental_accident>.

# Supplementary Tables

Supplementary Table S1: Variables used in the analysis pipeline. The table is available as a supplementary Excel file.

Supplementary Table S2: Mental health assessment battery.

| **Section** | **Variable^a^** | **Description^a^** |
| --- | --- | --- |
| PTSD assessment | PCL-5 score | PTSD total score, PCL-5, sum of all items |
|  | PTSD symptoms (at least one PCL-5 domain positive) | At least one PCL-5 domain positive |
|  | PCL-5 domain B score | PTSD rating, PCL-5 domain B |
|  | PTSD domain B symptoms | PTSD symptoms, PCL-5 domain B positive |
|  | PCL-5 domain C score | PTSD rating, PCL-5 domain C |
|  | PTSD domain C symptoms | PTSD symptoms, PCL-5 domain C positive |
|  | PCL-5 domain D score | PTSD rating, PCL-5 domain D |
|  | PTSD domain D symptoms | PTSD symptoms, PCL-5 domain D positive |
|  | PCL-5 domain E score | PTSD rating, PCL-5 domain E |
|  | PTSD domain E symptoms | PTSD symptoms, PCL-5 domain E positive |
| PTG assessment | PTGI score | Post-traumatic growth, PTGI total score, sum of all items |
|  | PTGI I relations score | Post-traumatic growth, PTGI scoring, domain I, relations |
|  | PTGI II possibilities score | Post-traumatic growth, PTGI scoring, domain II, new possibilities |
|  | PTGI III personal strength score | Post-traumatic growth, PTGI scoring, domain III, personal strength |
|  | PTGI IV spiritual score | Post-traumatic growth, PTGI scoring, domain IV, spiritual |
|  | PTGI V life appreciation score | Post-traumatic growth, PTGI scoring, domain V, appreciation of life |
| Mental health, resilience, coherence | RS13 score | Resilience, RS13 score |
|  | RS13 resilience class | Resilience, RS13 class |
|  | SOC-9L score | Lack of sense of coherence, SOC-9L score |
|  | PHQ-9 score | PHQ-9 score, depression |
|  | clinically relevant depression symptoms (PHQ-9 ≥10) | PHQ-9 score ≥ 10 points, clinically relevant depression symptoms |
|  | GAD-7 score | GAD-7 score, anxiety |
|  | clinically relevant anxiety symptoms (GAD-7 ≥10) | GAD-7 score ≥ 10 points, clinically relevant anxiety symptoms |
|  | PHQ-panic score | PHQ panic 4 item score |
|  | clinically relevant panic symptoms (PHQ-panic) | PHQ panic positivity |
|  | PHQ-15 score | PHQ-15 health problems, somatic symptoms |
|  | clinically relevant somatization symptoms (PHQ-15 ≥10) | PHQ-15 score ≥ 10 points, clinically relevant somatization symptoms |
| Quality of life | EUROHIS-QOL 8 mean score | Quality of life, EUROHIS-QOL 8 score, mean of all items |
|  | EUROHIS-QOL 8 QoL score | Quality of life, EUROHIS-QOL 8 score QoL |
|  | EUROHIS-QOL 8 health score | Quality of life, EUROHIS-QOL 8 score health |
|  | EUROHIS-QOL 8 energy score | Quality of life, EUROHIS-QOL 8 score energy |
|  | EUROHIS-QOL 8 finances score | Quality of life, EUROHIS-QOL 8 score financial aspects |
|  | EUROHIS-QOL 8 activity score | Quality of life, EUROHIS-QOL 8 score activity |
|  | EUROHIS-QOL 8 self-esteem score | Quality of life, EUROHIS-QOL 8 score self-esteem |
|  | EUROHIS-QOL 8 relationship score | Quality of life, EUROHIS-QOL 8 score relationship |
|  | EUROHIS-QOL 8 housing score | Quality of life, EUROHIS-QOL 8 score housing |
| ^a^GAD-7: 7-item general anxiety disorder scale; PHQ-9: 9-item patient health questionnaire for depressive symptoms; PHQ-15: 15-item patient health questionnaire for common somatic symptoms as a substitute for somatization; PHQ-panic: patient health questionnaire module for panic disorders; EUROHIS-QOL 8: 8-item EUROHIS project quality of life scale; SOC-9L: Leipzig 9-item sense of coherence questionnaire; RS13: 13-item resilience scale; PCL-5: PTSD checklist for DSM-5; PTGI: post-traumatic growth inventory | | |

Supplementary Table S3: Consistency of the psychometric tools used in the study measured by McDonald's omega.

| **Scale^a^** | **Number of latent factors** | **Total omega** |
| --- | --- | --- |
| PTGI | 4 | 0.97 |
| RS13 | 3 | 0.94 |
| PCL-5 | 4 | 0.92 |
| SOC-9L | 3 | 0.89 |
| GAD-7 | 3 | 0.89 |
| PHQ-panic | 1 | 0.88 |
| EUROHIS-QOL 8 | 4 | 0.88 |
| PHQ-9 | 4 | 0.87 |
| PHQ-15 | 4 | 0.84 |
| ^a^GAD-7: 7-item general anxiety disorder scale; PHQ-9: 9-item patient health questionnaire for depressive symptoms; PHQ-15: 15-item patient health questionnaire for common somatic symptoms as a substitute for somatization; PHQ-panic: patient health questionnaire module for panic disorders; EUROHIS-QOL 8: 8-item EUROHIS project quality of life scale; SOC-9L: Leipzig 9-item sense of coherence questionnaire; RS13: 13-item resilience scale; PCL-5: PTSD checklist for DSM-5; PTGI: post-traumatic growth inventory | | |

Supplementary Table S4: Significant differences between patients who did not respond to the study invitation and the analyzed study participants. Numeric variables are presented as medians with interquartile ranges (IQR). Categorical variables are presented as percentages and counts within the complete observation set.

| **Variable^a^** | **Included** | **No response** | **Significance^b^** | **Effect size^b^** |
| --- | --- | --- | --- | --- |
| mountain sport type | alpine skiing/snowboarding: 59% (n = 180) ski touring/freeride: 3% (n = 9) cross-country skiing: 5.6% (n = 17) sledding: 4% (n = 12) ice climbing: 0.33% (n = 1) hiking: 5.6% (n = 17) climbing: 3.6% (n = 11) mountaineering: 0.66% (n = 2) biking: 16% (n = 48) air sports: 0.33% (n = 1) water sports: 0.33% (n = 1) other: 1.3% (n = 4) n = 303 | alpine skiing/snowboarding: 51% (n = 2089) ski touring/freeride: 2.6% (n = 105) cross-country skiing: 2.1% (n = 87) sledding: 4.8% (n = 198) ice climbing: 0.15% (n = 6) hiking: 5.1% (n = 211) climbing: 2.7% (n = 110) mountaineering: 0.41% (n = 17) biking: 27% (n = 1127) air sports: 0.17% (n = 7) water sports: 0.15% (n = 6) other: 3.4% (n = 139) n = 4102 | p < 0.001 | V = 0.096 |
| injury severity class, AIS | 1: 37% (n = 108) 2: 35% (n = 103) 3+: 28% (n = 83) 0: 0% (n = 0) n = 294 | 1: 46% (n = 124) 2: 35% (n = 93) 3+: 17% (n = 46) 0: 1.5% (n = 4) n = 267 | p = 0.0019 | V = 0.16 |
| injury severity, AIS | 2 [IQR: 1 - 3] range: 1 - 5 n = 294 | 2 [IQR: 1 - 2] range: 0 - 4 n = 267 | p < 0.001 | r = 0.15 |
| hospitalized | 26% (n = 80) n = 307 | 9.4% (n = 393) n = 4172 | p < 0.001 | V = 0.14 |
| surgical therapy | 14% (n = 43) n = 307 | 4.2% (n = 175) n = 4172 | p < 0.001 | V = 0.12 |
| number of surgical ICD-10 diagnoses | none: 86% (n = 264) 1: 8.5% (n = 26) 2+: 5.5% (n = 17) n = 307 | none: 96% (n = 3997) 1: 2.8% (n = 116) 2+: 1.4% (n = 59) n = 4172 | p < 0.001 | V = 0.12 |
| ^a^AIS: abbreviated injury scale; ICD-10: 10th version of the International Statistical Classification of Diseases and Related Health Problems. | | | | |
| ^b^Numeric variables: Mann-Whitney test with r effect size statistic; categorical variables: χ² test with Cramer V effect size statistic. P values corrected for multiple testing with the false discovery rate method. | | | | |

Supplementary Table S5: Significant differences between the study survey responders excluded from analysis due to missingness of psychometric data and the analyzed study participants. Numeric variables are presented as medians with interquartile ranges (IQR). Categorical variables are presented as percentages and counts within the complete observation set.

| **Variable^a^** | **Included** | **Incomplete variables** | **Significance^b^** | **Effect size^b^** |
| --- | --- | --- | --- | --- |
| income/year | none: 21% (n = 63) < 30K EUR: 18% (n = 56) 30K - 45K EUR: 19% (n = 59) ≥ 45K EUR: 42% (n = 129) n = 307 | none: 44% (n = 35) < 30K EUR: 14% (n = 11) 30K - 45K EUR: 16% (n = 13) ≥ 45K EUR: 26% (n = 21) n = 80 | p < 0.001 | V = 0.22 |
| injury severity, AIS | 2 [IQR: 1 - 3] range: 1 - 5 n = 294 | 2 [IQR: 1 - 2] range: 1 - 4 n = 70 | p = 0.031 | r = 0.11 |
| upper limb injury | 41% (n = 120) n = 294 | 57% (n = 40) n = 70 | p = 0.019 | V = 0.13 |
| hospitalized | 26% (n = 80) n = 307 | 14% (n = 11) n = 80 | p = 0.03 | V = 0.12 |
| surgical therapy | 14% (n = 43) n = 307 | 5% (n = 4) n = 80 | p = 0.045 | V = 0.11 |
| persisting physical health consequences of the accident | 37% (n = 115) n = 307 | 22% (n = 12) n = 55 | p = 0.037 | V = 0.12 |
| ^a^AIS: abbreviated injury scale; K: 1000 Euro; EUR: Euro. | | | | |
| ^b^Numeric variables: Mann-Whitney test with r effect size statistic; categorical variables: χ² test with Cramer V effect size statistic. P values corrected for multiple testing with the false discovery rate method. | | | | |

Supplementary Table S6: Comparison of sociodemographic features of the study cohort and estimates for the general Austrian population. Numeric variables are presented as medians with interquartile ranges (IQR). Categorical variables are presented as percentages and counts within the complete observation set.

| **Variable** | **Cohort** | **Austrian population** | **Significance^b^** | **Effect size^b^** |
| --- | --- | --- | --- | --- |
| age, years | 51 [IQR: 33 - 60] range: 18 - 82 n = 307 | 48 [IQR: 34 - 62] range: 18 - 82 n = 7196298 | ns (p = 0.99) | r = 4.9e-06 |
| age, class, years | 18-30: 20% (n = 61) 31-65: 66% (n = 202) >65: 14% (n = 44) n = 307 | 18-30: 20% (n = 1404528) 31-65: 62% (n = 4452200) >65: 19% (n = 1339570) n = 7196298 | ns (p = 0.15) | V = 0.00073 |
| sex | female: 45% (n = 137) male: 55% (n = 170) n = 307 | female: 51% (n = 3643212) male: 49% (n = 3553086) n = 7196298 | p = 0.041 | V = 0.00078 |
| highest education grade | primary/apprenticeship: 16% (n = 49) secondary: 38% (n = 115) tertiary: 45% (n = 136) n = 300 | primary/apprenticeship: 50% (n = 2511913) secondary: 30% (n = 1472534) tertiary: 20% (n = 995515) n = 4979962 | p < 0.001 | V = 0.0059 |
| employment | employed: 68% (n = 210) unemployed: 3.6% (n = 11) student: 10% (n = 32) retired: 18% (n = 54) n = 307 | employed: 58% (n = 4438154) unemployed: 12% (n = 921539) student: 4.4% (n = 336979) retired: 26% (n = 1980715) n = 7677387 | p < 0.001 | V = 0.0027 |
| smoking | 7.8% (n = 24) n = 307 | 26% (n = 1943745) n = 7417876 | p < 0.001 | V = 0.0027 |
| physical or mental illness^a^ | 20% (n = 60) n = 307 | 38% (n = 2841553) n = 7417876 | p < 0.001 | V = 0.0025 |
| ^a^In the study survey: participants reporting a chronic pre-existing physical illness or a mental disorder diagnosed by a medical professional. | | | | |
| ^b^Numeric variables: Mann-Whitney test with r effect size statistic, categorical variables: χ² test with Cramer V effect size statistic. | | | | |

Supplementary Table S7: Significant differences between the training and test subset of the study cohort. Numeric variables are presented as medians with interquartile ranges (IQR). Categorical variables are presented as percentages and counts within the complete observation set.

| **Variable^a^** | **Training** | **Test** | **Significance^b^** | **Effect size^b^** |
| --- | --- | --- | --- | --- |
| RS13 resilience class | low: 23% (n = 53) moderate: 13% (n = 29) high: 64% (n = 148) n = 230 | low: 3.9% (n = 3) moderate: 17% (n = 13) high: 79% (n = 61) n = 77 | p = 0.035 | V = 0.22 |
| EUROHIS-QOL 8 mean score | 4.2 [IQR: 3.9 - 4.6] range: 2 - 5 n = 230 | 4.5 [IQR: 4.2 - 4.6] range: 2.8 - 5 n = 77 | p = 0.049 | r = 0.18 |
| EUROHIS-QOL 8 QoL score | 5 [IQR: 4 - 5] range: 3 - 5 n = 230 | 5 [IQR: 5 - 5] range: 3 - 5 n = 77 | p = 0.0011 | r = 0.25 |
| ^a^RS13: 13-item resilience scale; EUROHIS-QOL 8: 8-item EUROHIS project quality of life scale. | | | | |
| ^b^Numeric variables: Mann-Whitney test with r effect size statistic; categorical variables: χ² test with Cramer V effect size statistic. P values corrected for multiple testing with the false discovery rate method. | | | | |

Supplementary Table S8: Differences in psychometric clustering factors between the mental health clusters. Numeric variables are presented as medians with interquartile ranges (IQR). Statistical significance was determined by false discovery rate-corrected Kruskal-Wallis test with eta-square effect size statistic. The table is available in a supplementary Excel file.

Supplementary Table S9: Frequency of symptoms of mental disorders in the mental health clusters in the entire cohort. Categorical variables are presented as percentages and counts within the clusters.

| **Variable^a^** | **Neutral cluster** | **PTG cluster** | **PTS cluster** | **Significance^b^** | **Effect size^b^** |
| --- | --- | --- | --- | --- | --- |
| Participants, n | 103 | 94 | 110 |  |  |
| PTSD symptoms (at least one PCL-5 domain positive) | 4.9% (n = 5) | 15% (n = 14) | 35% (n = 39) | p < 0.001 | V = 0.33 |
| PTSD domain B symptoms | 1.9% (n = 2) | 9.6% (n = 9) | 20% (n = 22) | p < 0.001 | V = 0.24 |
| PTSD domain C symptoms | 2.9% (n = 3) | 5.3% (n = 5) | 15% (n = 17) | p = 0.0078 | V = 0.2 |
| PTSD domain D symptoms | 0.97% (n = 1) | 3.2% (n = 3) | 11% (n = 12) | p = 0.011 | V = 0.2 |
| PTSD domain E symptoms | 0% (n = 0) | 5.3% (n = 5) | 17% (n = 19) | p < 0.001 | V = 0.27 |
| flashbacks during mountain sport | 24% (n = 25) | 35% (n = 33) | 58% (n = 64) | p < 0.001 | V = 0.3 |
| flashback frequency during mountain sport | none: 76% (n = 78) > 1/year: 17% (n = 18) > 1/month: 6.8% (n = 7) | none: 65% (n = 61) > 1/year: 20% (n = 19) > 1/month: 15% (n = 14) | none: 42% (n = 46) > 1/year: 28% (n = 31) > 1/month: 30% (n = 33) | p < 0.001 | V = 0.22 |
| RS13 resilience class | low: 3.9% (n = 4) moderate: 5.8% (n = 6) high: 90% (n = 93) | low: 6.4% (n = 6) moderate: 5.3% (n = 5) high: 88% (n = 83) | low: 42% (n = 46) moderate: 28% (n = 31) high: 30% (n = 33) | p < 0.001 | V = 0.43 |
| clinically relevant depression symptoms (PHQ-9 ≥10) | 0% (n = 0) | 2.1% (n = 2) | 18% (n = 20) | p < 0.001 | V = 0.32 |
| clinically relevant anxiety symptoms (GAD-7 ≥10) | 0% (n = 0) | 0% (n = 0) | 8.2% (n = 9) | p = 0.0016 | V = 0.23 |
| clinically relevant panic symptoms (PHQ-panic positive) | 0% (n = 0) | 0% (n = 0) | 2.7% (n = 3) | ns (p = 0.15) | V = 0.13 |
| clinically relevant somatization symptoms (PHQ-15 ≥10) | 0.97% (n = 1) | 3.2% (n = 3) | 13% (n = 14) | p = 0.0027 | V = 0.22 |
| ^a^GAD-7: 7-item general anxiety disorder scale; PHQ-9: 9-item patient health questionnaire for depressive symptoms; PHQ-15: 15-item patient health questionnaire for common somatic symptoms as a substitute for somatization; PHQ-panic: patient health questionnaire module for panic disorders; RS13: 13-item resilience scale; PCL-5: PTSD checklist for DSM-5; PTSD: post-traumatic stress disorder. | | | | | |
| ^b^χ² test with Cramer V effect size statistic. P values were corrected for multiple testing with the false discovery rate method. | | | | | |

Supplementary Table S10: Differences in demographic, socioeconomic, clinical, accident- and recovery-related factors, and between the mental clusters in the entire cohort. Significant effects are presented, the full table is available as a supplementary Excel file. Numeric variables are presented as medians with interquartile ranges (IQR). Categorical variables are presented as percentages and counts within the clusters.

| **Variable^a^** | **Neutral cluster** | **PTG cluster** | **PTS cluster** | **Significance^b^** | **Effect size^b^** |
| --- | --- | --- | --- | --- | --- |
| age at accident, years | 53 [IQR: 34 - 61] range: 18 - 82 | 54 [IQR: 39 - 61] range: 18 - 81 | 45 [IQR: 29 - 56] range: 18 - 82 | p = 0.023 | η² = 0.027 |
| pre-existing physical illness | 5.8% (n = 6) | 22% (n = 21) | 18% (n = 20) | p = 0.012 | V = 0.19 |
| pre-existing diagnosed mental disorder | 0% (n = 0) | 1.1% (n = 1) | 14% (n = 15) | p < 0.001 | V = 0.28 |
| psychological/psychiatric support need post accident | 0% (n = 0) | 8.5% (n = 8) | 14% (n = 15) | p = 0.0033 | V = 0.22 |
| physical health consequences of the accident | 27% (n = 28) | 32% (n = 30) | 52% (n = 57) | p = 0.0023 | V = 0.23 |
| caution during mountain sport post accident | no change: 50% (n = 52) more cautious: 49% (n = 50) less cautious: 0.97% (n = 1) | no change: 32% (n = 30) more cautious: 67% (n = 63) less cautious: 1.1% (n = 1) | no change: 22% (n = 24) more cautious: 78% (n = 86) less cautious: 0% (n = 0) | p = 0.0016 | V = 0.19 |
| ^a^psychological support need: subjective need for psychological or psychiatric support following the accident; physical health consequences: physical health consequences related to the accident. | | | | | |
| ^b^Numeric variables: Kruskal-Wallis test with η² effect size statistic. Categorical variables: χ² test with Cramer V effect size statistic. P values were corrected for multiple testing with the false discovery rate method. | | | | | |

Supplementary Table S11: Sets of explanatory factors used for modeling of the mental health cluster assignment.

| **Classifier type^a^** | **Explanatory variables** |
| --- | --- |
| early predictor model | age at accident, age at accident, class, accident season, accident daytime, sex, highest education grade, employment at accident, mountain sport profession, search and rescue profession, healthcare profession, income/year, residence in the Alps, smoking, pre-existing physical illness, pre-existing physical illness type, pre-existing diagnosed mental disorder, number of prior traumatic events/DIA-X, problematic alcohol use (CAGE ≥2), prior mountain sport accidents, mountain sport type, alone during the accident, responsible for the accident, number of injured persons, rescue mode, professional rescue mode, injury severity class, injury severity, head injury, face injury, neck injury, chest injury, abdominal injury, spine region injury, upper limb injury, lower limb injury, other injury, number of injured body parts, hospitalization, surgical therapy, number of surgical ICD-10 diagnoses, psychological/psychiatric support post accident, psychological/psychiatric support need post accident, physical health consequences of the accident, returned to same mountain sport post accident, caution during mountain sport post accident, confusion during mountain sport |
| full set predictor model | age at accident, age at accident, class, accident season, accident daytime, sex, highest education grade, employment at accident, mountain sport profession, search and rescue profession, healthcare profession, income/year, residence in the Alps, smoking, pre-existing physical illness, pre-existing physical illness type, pre-existing diagnosed mental disorder, number of prior traumatic events/DIA-X, problematic alcohol use (CAGE ≥2), prior mountain sport accidents, mountain sport type, alone during the accident, responsible for the accident, number of injured persons, rescue mode, professional rescue mode, injury severity class, injury severity, head injury, face injury, neck injury, chest injury, abdominal injury, spine region injury, upper limb injury, lower limb injury, other injury, number of injured body parts, hospitalization, surgical therapy, number of surgical ICD-10 diagnoses |
| ^a^early predictors: variables available during acute medical management of the patient; full predictor set: variables available during acute medical management of the patient and during follow-up. | |

Supplementary Table S12: The optimal combinations of machine learning algorithm parameters found in 10-fold cross-validation of the training subset of the study cohort.

| **Classifier type^a^** | **Algorithm^b^** | **Tuning parameters** |
| --- | --- | --- |
| full | RF | mtry = 46, splitrule = extratrees, min.node.size = 1 |
|  | NNet | size = 9, decay = 0.01 |
|  | SVM/radial | sigma = 0.07, C = 2.5 |
|  | RPart | cp = 0.045 |
|  | SDA | diagonal = FALSE, lambda = 0.1 |
|  | cForest | mtry = 11 |
|  | ElasticNet | alpha = 0.5, lambda = 0.0149 |
| early | RF | mtry = 9, splitrule = extratrees, min.node.size = 5 |
|  | NNet | size = 8, decay = 0.01 |
|  | SVM/radial | sigma = 0.01, C = 0.3 |
|  | RPart | cp = 0.025 |
|  | SDA | diagonal = FALSE, lambda = 0.99 |
|  | cForest | mtry = 6 |
|  | ElasticNet | alpha = 0.5, lambda = 0.0291 |
| ^a^early predictors: variables available during acute medical management of the patient; full predictor set: variables available during acute medical management of the patient and during follow-up. | | |
| ^b^RF: random forest; NNet: neural network with a single hidden layer; SVM/radial: support vector machines with radial kernel; RPart: recursive partitioning; SDA: shrinkage discriminant analysis; cForest: conditional random forest; Elastic Net: elastic net multinomial regression. | | |

Supplementary Table S13: Performance statistics of machine learning classifiers at predicting the mental health cluster assignment. Models employing early predictors available during acute medical management of the patient.

| **Algorithm^a^** | **Data subset^b^** | **Accuracy** | **Cohen's κ** | **Brier score** | **Sensitivity, PTS cluster** | **Specificity, PTS cluster** |
| --- | --- | --- | --- | --- | --- | --- |
| RF | training | 0.99 | 0.980 | 0.2300 | 1.00 | 0.99 |
|  | 10-fold CV | 0.42 | 0.120 | 0.6700 | 0.42 | 0.71 |
|  | test | 0.46 | 0.200 | 0.6600 | 0.46 | 0.70 |
| NNet | training | 1.00 | 1.000 | 0.0003 | 1.00 | 1.00 |
|  | 10-fold CV | 0.45 | 0.180 | 0.9400 | 0.47 | 0.69 |
|  | test | 0.39 | 0.092 | 1.0000 | 0.31 | 0.67 |
| SVM/radial | training | 0.56 | 0.340 | 0.5800 | 0.58 | 0.77 |
|  | 10-fold CV | 0.42 | 0.150 | 0.6600 | 0.38 | 0.77 |
|  | test | 0.38 | 0.070 | 0.6700 | 0.35 | 0.65 |
| RPart | training | 0.56 | 0.330 | 0.5600 | 0.65 | 0.74 |
|  | 10-fold CV | 0.40 | 0.094 | 0.7000 | 0.44 | 0.75 |
|  | test | 0.39 | 0.100 | 0.7200 | 0.38 | 0.70 |
| SDA | training | 0.61 | 0.420 | 0.5400 | 0.54 | 0.88 |
|  | 10-fold CV | 0.42 | 0.130 | 0.8600 | 0.36 | 0.73 |
|  | test | 0.44 | 0.170 | 0.8100 | 0.38 | 0.74 |
| cForest | training | 0.65 | 0.470 | 0.5900 | 0.71 | 0.80 |
|  | 10-fold CV | 0.40 | 0.094 | 0.6600 | 0.47 | 0.64 |
|  | test | 0.42 | 0.140 | 0.6600 | 0.46 | 0.70 |
| ElasticNet | training | 0.69 | 0.530 | 0.4400 | 0.63 | 0.85 |
|  | 10-fold CV | 0.46 | 0.190 | 0.7300 | 0.41 | 0.69 |
|  | test | 0.39 | 0.091 | 0.7000 | 0.46 | 0.65 |
| ^a^RF: random forest; NNet: neural network with a single hidden layer; SVM/radial: support vector machines with radial kernel; RPart: recursive partitioning; SDA: shrinkage discriminant analysis; cForest: conditional random forest; Elastic Net: elastic net multinomial regression. | | | | | | |
| ^b^CV: cross-validation | | | | | | |

Supplementary Table S14: Performance statistics of machine learning classifiers at predicting the mental health cluster assignment. Models employing the full predictor set available during acute medical management of the patient and follow-up.

| **Algorithm^a^** | **Data subset^b^** | **Accuracy** | **Cohen's κ** | **Brier score** | **Sensitivity, PTS cluster** | **Specificity, PTS cluster** |
| --- | --- | --- | --- | --- | --- | --- |
| RF | training | 1.00 | 1.000 | 0.08800 | 1.00 | 1.00 |
|  | 10-fold CV | 0.46 | 0.190 | 0.64000 | 0.47 | 0.73 |
|  | test | 0.46 | 0.200 | 0.64000 | 0.46 | 0.76 |
| NNet | training | 1.00 | 1.000 | 0.00021 | 1.00 | 1.00 |
|  | 10-fold CV | 0.47 | 0.200 | 0.92000 | 0.42 | 0.68 |
|  | test | 0.36 | 0.055 | 1.10000 | 0.27 | 0.70 |
| SVM/radial | training | 0.96 | 0.950 | 0.34000 | 0.97 | 0.99 |
|  | 10-fold CV | 0.46 | 0.180 | 0.65000 | 0.56 | 0.64 |
|  | test | 0.44 | 0.170 | 0.66000 | 0.46 | 0.67 |
| RPart | training | 0.48 | 0.210 | 0.61000 | 0.65 | 0.68 |
|  | 10-fold CV | 0.43 | 0.130 | 0.65000 | 0.49 | 0.72 |
|  | test | 0.36 | 0.071 | 0.66000 | 0.42 | 0.78 |
| SDA | training | 0.81 | 0.710 | 0.30000 | 0.78 | 0.90 |
|  | 10-fold CV | 0.47 | 0.210 | 0.82000 | 0.46 | 0.71 |
|  | test | 0.44 | 0.180 | 0.84000 | 0.35 | 0.80 |
| cForest | training | 0.67 | 0.510 | 0.56000 | 0.77 | 0.81 |
|  | 10-fold CV | 0.45 | 0.160 | 0.64000 | 0.51 | 0.72 |
|  | test | 0.39 | 0.100 | 0.66000 | 0.42 | 0.74 |
| ElasticNet | training | 0.82 | 0.720 | 0.32000 | 0.79 | 0.91 |
|  | 10-fold CV | 0.49 | 0.230 | 0.74000 | 0.49 | 0.70 |
|  | test | 0.42 | 0.140 | 0.81000 | 0.31 | 0.78 |
| ^a^RF: random forest; NNet: neural network with a single hidden layer; SVM/radial: support vector machines with radial kernel; RPart: recursive partitioning; SDA: shrinkage discriminant analysis; cForest: conditional random forest; Elastic Net: elastic net multinomial regression. | | | | | | |
| ^b^CV: cross-validation | | | | | | |

# Supplementary Figures


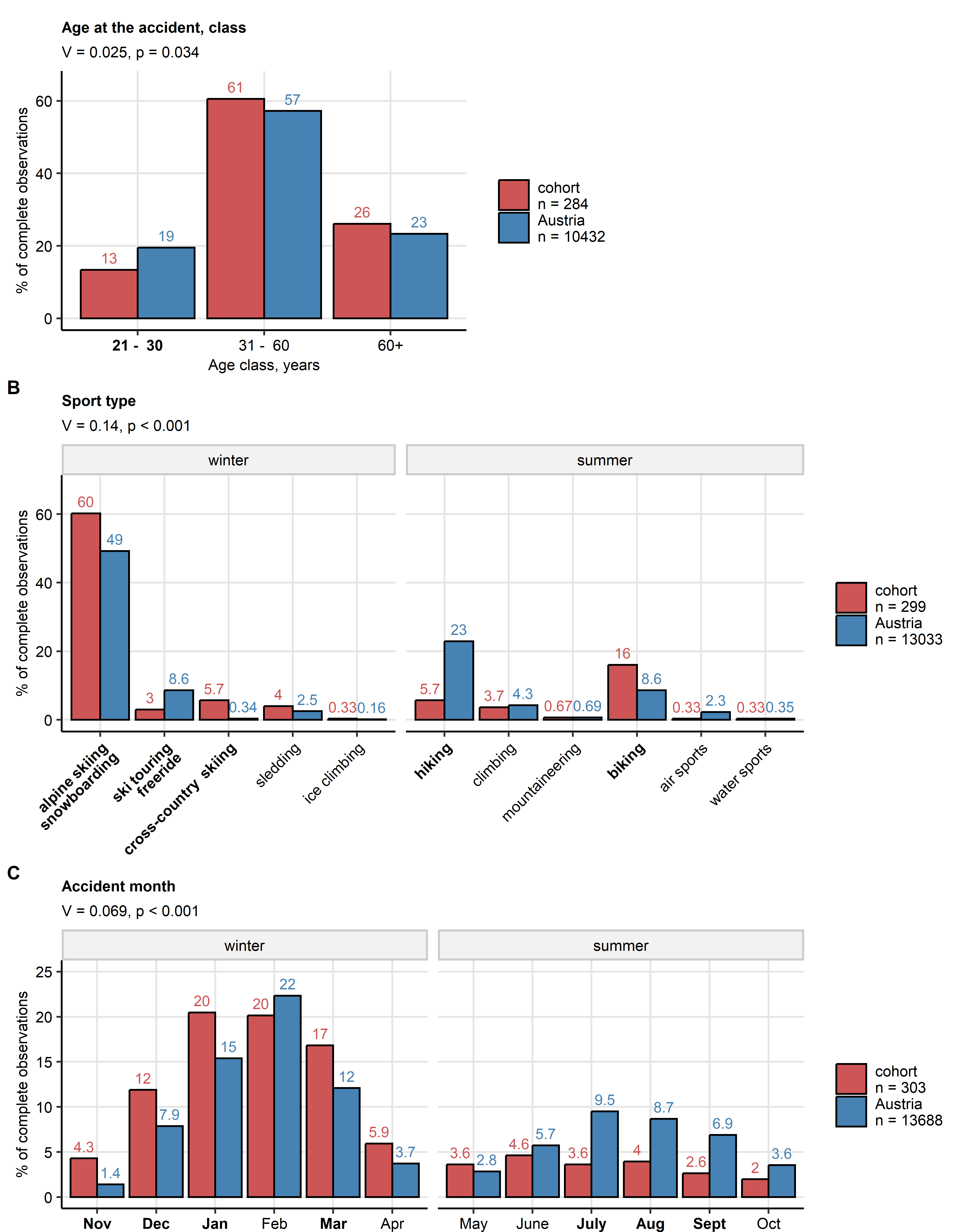


**Supplementary Figure S1. Age, mountain sport type, and accident month in the study cohort and Austrian national registry of mountain accidents.**

*Age class distribution (A), mountain sport types (B), and accident months (C) were compared between the study cohort and victims of mountain sport accidents in Austria in 2023. Frequencies for the nation-wide data set of mountain accidents(recorded by the alpine police) were extracted from the annual report of the Austrian Board of Mountain Safety. In the comparison of the age class distribution, only individuals older than 20 years were included. In the comparison of sport types, non-sportive mountain accidents (e.g. during hunting or forestry) were excluded from the national statistic. Non-classifiable sport types (category ‘other’) in the study cohort were excluded from the analysis presented in (C). Statistical significance of the global differences was determined by* $\chi^{2}$ *test with Cramer’s V effect size statistic. Pairwise differences for particular sport types, months and age classes were assessed by* $\chi^{2}$ *test corrected for multiple testing with the Holm method. Percentages of categories within the complete observation sets are presented as bar plots. Bars are labeled with the corresponding percent values. Total observation numbers are displayed in the plot legends. Global effect sizes and p values are presented in the plot captions. Significant results of post-hoc tests are highlighted with bold X axis labels.*

*alpine skiing: alpine skiing and snowboarding in secured areas of ski resorts; ski touring/freeride: alpine skiing and snowboarding performed outside of secured areas of ski resorts; sledding: sledding and bob sledding; ice climbing: ice and mixed climbing; climbing: bouldering, sport and rock climbing; mountaineering: high mountain and glacier tours; biking: road, cross-country and mountain biking.*


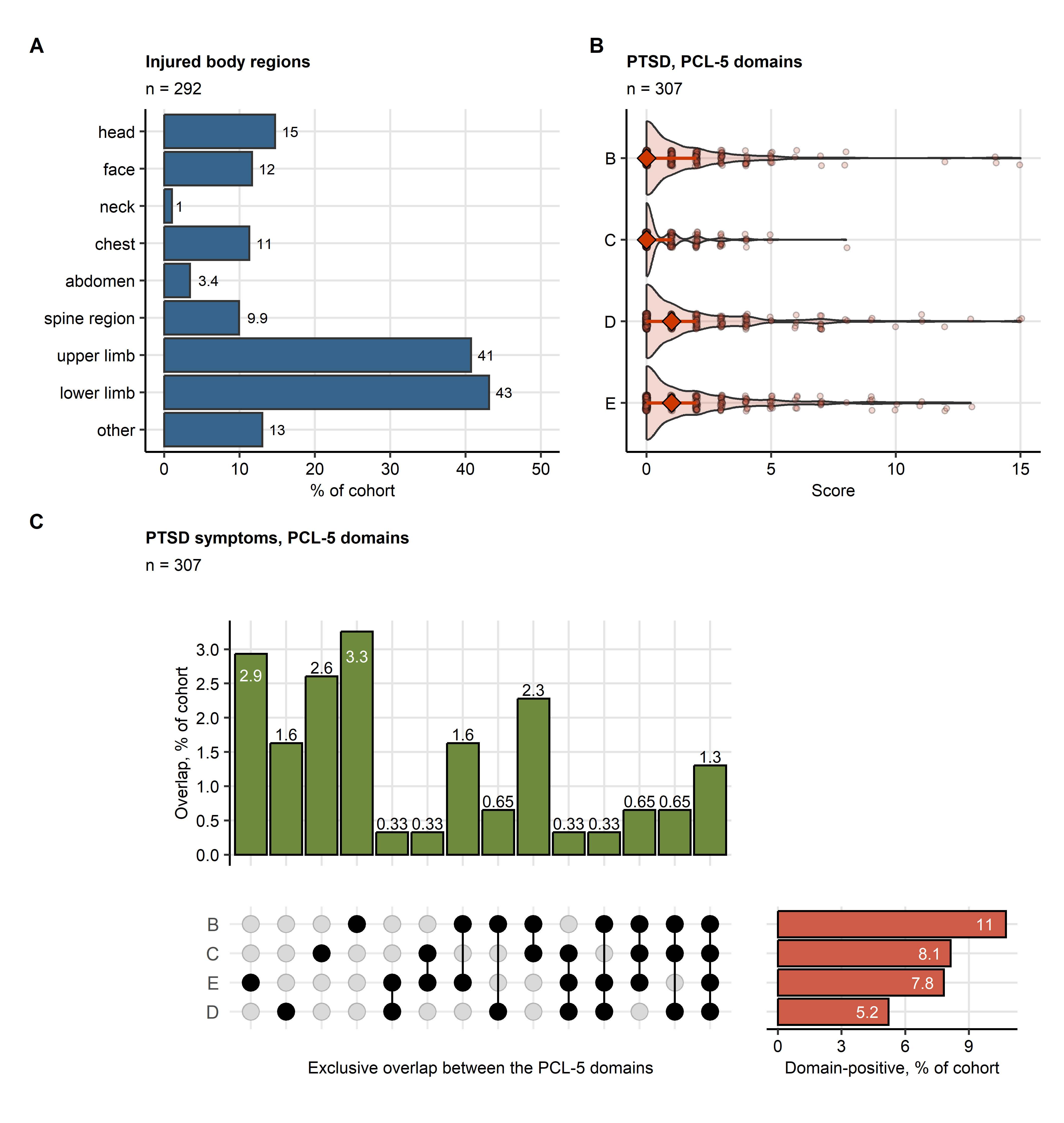


**Supplementary Figure S2. Injured body regions and symptoms of PTSD in the study cohort.**

*(A) Distribution of injured body regions presented in a bar plot. The number of complete observations is indicated in the plot caption.*

*(B) Scores of particular domains of the PCL-5 tool evaluating symptoms of post-traumatic stress disorder (PTSD) presented in violin plots. Single observations are visualized as points. Medians and interquartile ranges are represented by red diamonds and whiskers. The number of complete observations is indicated in the plot caption.*

*(C) Frequency and overlap of clinically relevant PTSD symptoms captured by positivity of particular domains of the PCL-5 scale presented in an upset plot. Percentages of participants screened positive for the domain B, C, D or E PTSD symptoms are displayed in as red bars. Percentages of participants with single and overlapping domain B, C, D or E PTSD symptoms are presented as green bars. The number of complete observations is indicated in the plot caption.*


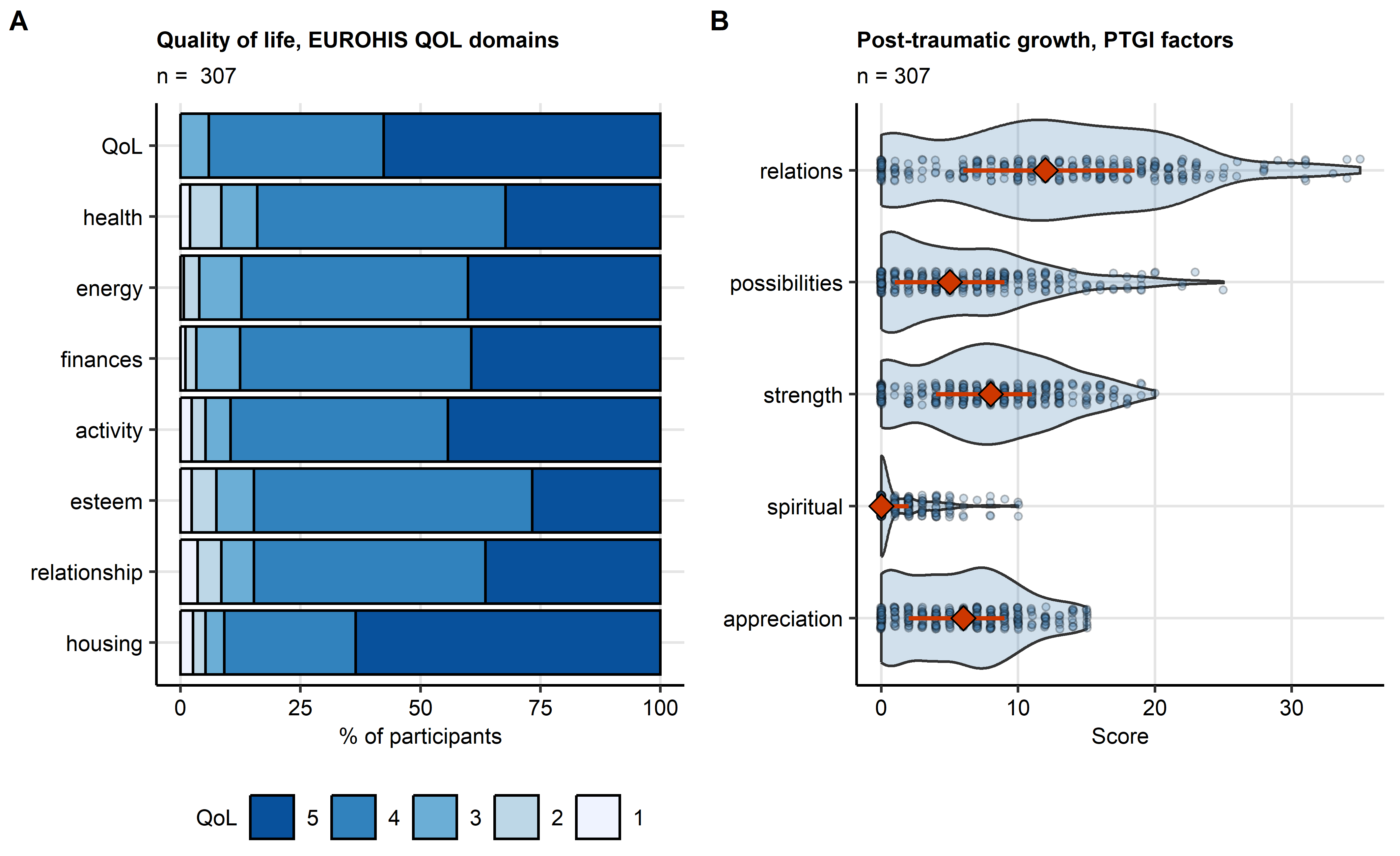


**Supplementary Figure S3. Scores of quality of life and post-traumatic growth in the study cohort.**

*(A) Scores of the domains of the EUROHIS project 8-item quality of life scale (EUROHIS-QOL 8) presented in a bar plot. The number of complete observations is displayed in the plot caption.*

*(B) Scores of the domains of the post-traumatic growth inventory (PTGI) scale presented in a violin plots. Single observations are visualized as points. Red diamonds with whiskers represent medians with interquartile ranges. The number of complete observations is indicated in the plot caption.*


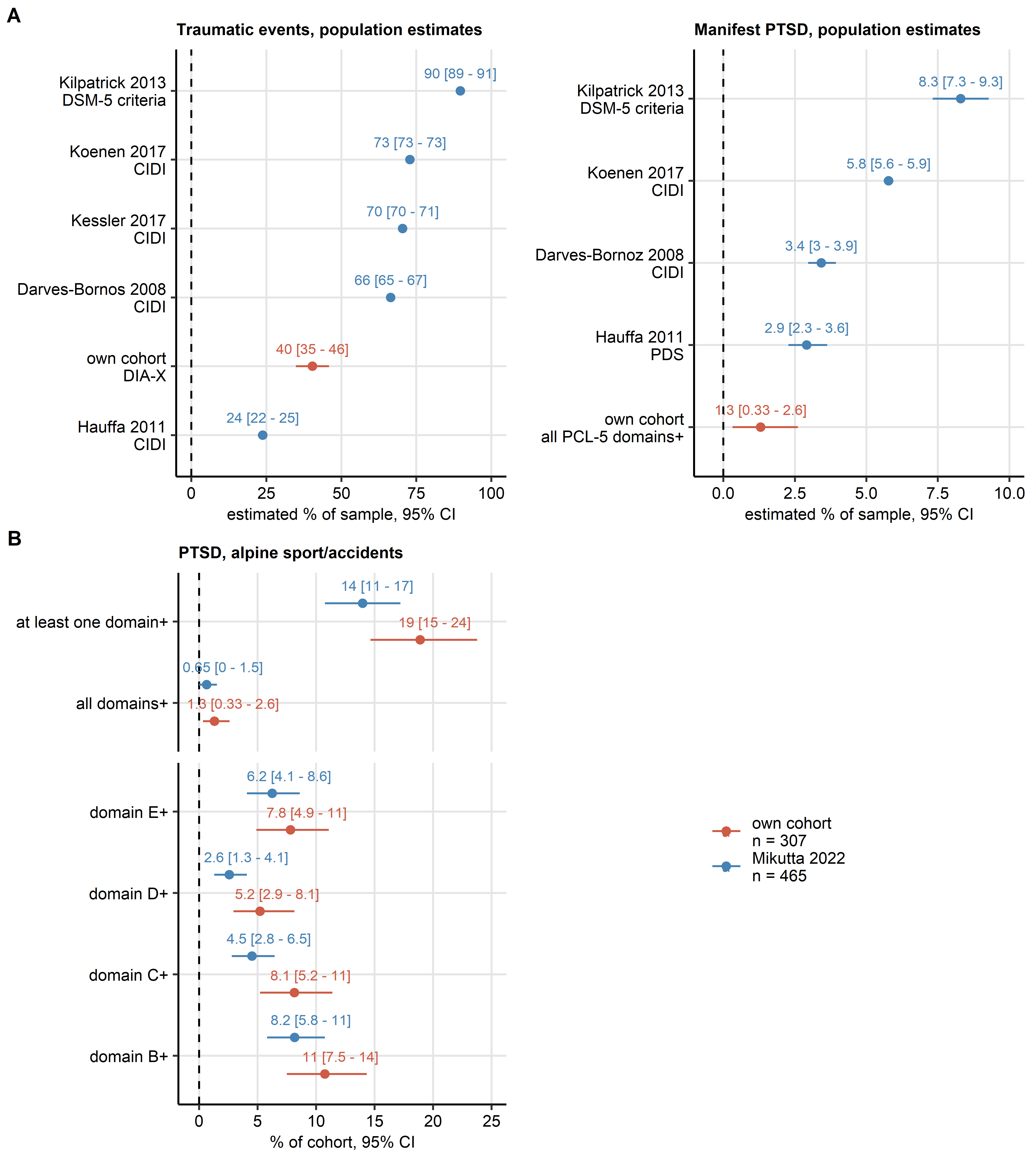


**Supplementary Figure S4. Comparison of frequency of traumatic events, manifest PTSD, and symptoms of PTSD in the study cohort and literature.**

*Frequency estimates of traumatic events, manifest PTSD and symptoms of PTSD with bias-corrected and accelerated (BCA) 95% confidence computed by bootstrap are depicted in Forest plots.*

*(A) Comparison of frequencies of traumatic events and manifest PTSD in the study cohort with estimates of population prevalence of lifetime traumatic events and manifest PTSD in published surveys. Tools used for identification of traumatic events and PTSD symptoms are indicated in the Y axis (CIDI: Composite International diagnostic interview, DSM-5: Diagnostic and Statistical Manual of Mental Disorders, 5^th^ edition, PDS: post-traumatic stress diagnostic scale). Note that in the present study we specifically asked to rate PTSD symptoms related to the mountain accident of interest.*

*(B) Comparison of manifest PTSD and symptoms of PTSD symptoms in the study cohort and the cohort of Swiss mountain rescuers reported by Mikutta and colleagues. PTSD symptoms were assessed by the PCL-5 tool and are subsumed under the B, C, B, and E domains. Manifest PTSD is defined as positivity for all domains of the PCL-5 instrument. Note that in the present study we specifically asked to rate PTSD symptoms related to the mountain accident of interest.*


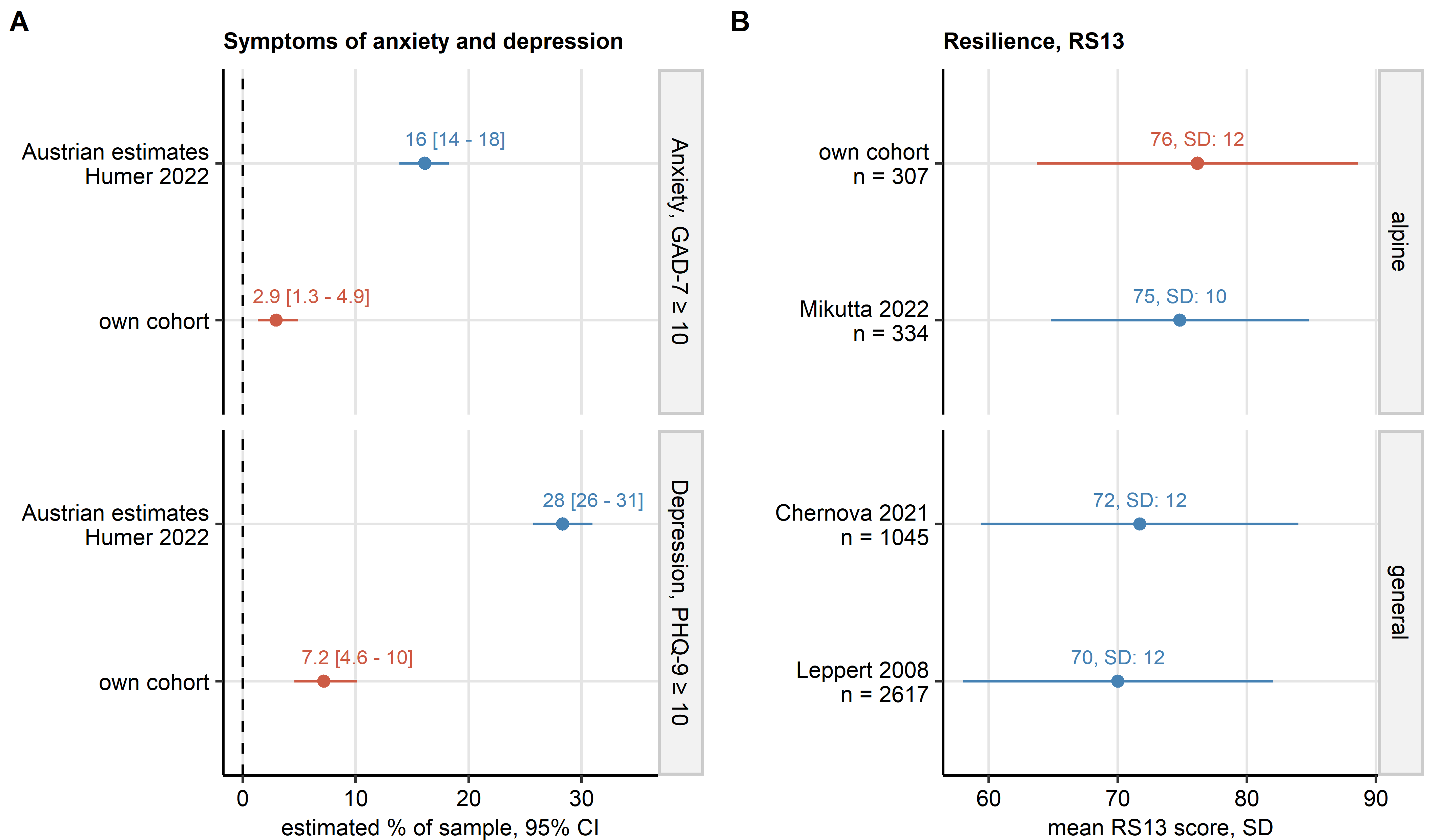


**Supplementary Figure S5. Comparison of frequency of clinically relevant symptoms of anxiety and depression, and of resilience scoring in the study cohort and literature.**

*Frequency estimates of mental disorder symptoms with bias-corrected and accelerated (BCA) 95% confidence computed by bootstrap are depicted in Forest plots. Mean resilience scores obtained with the RS13 tool with standard deviations (SD) are shown in a Forest plot.*

*(A) Comparison of frequency of clinically relevant symptoms of anxiety and depression measured by the GAD-7 and PHQ-9 instruments in the study cohort with symptoms reported in a representative sample of Austrian population in 2022 by Humer and colleagues.*

*(C) Comparison of scores of resilience in the study cohort, the cohort of Swiss mountain rescuers published by Mikutta et al., and samples of the Tyrolean (Chernova et al.) and German population (Leppert et al.) investigated with the RS13 instrument.*


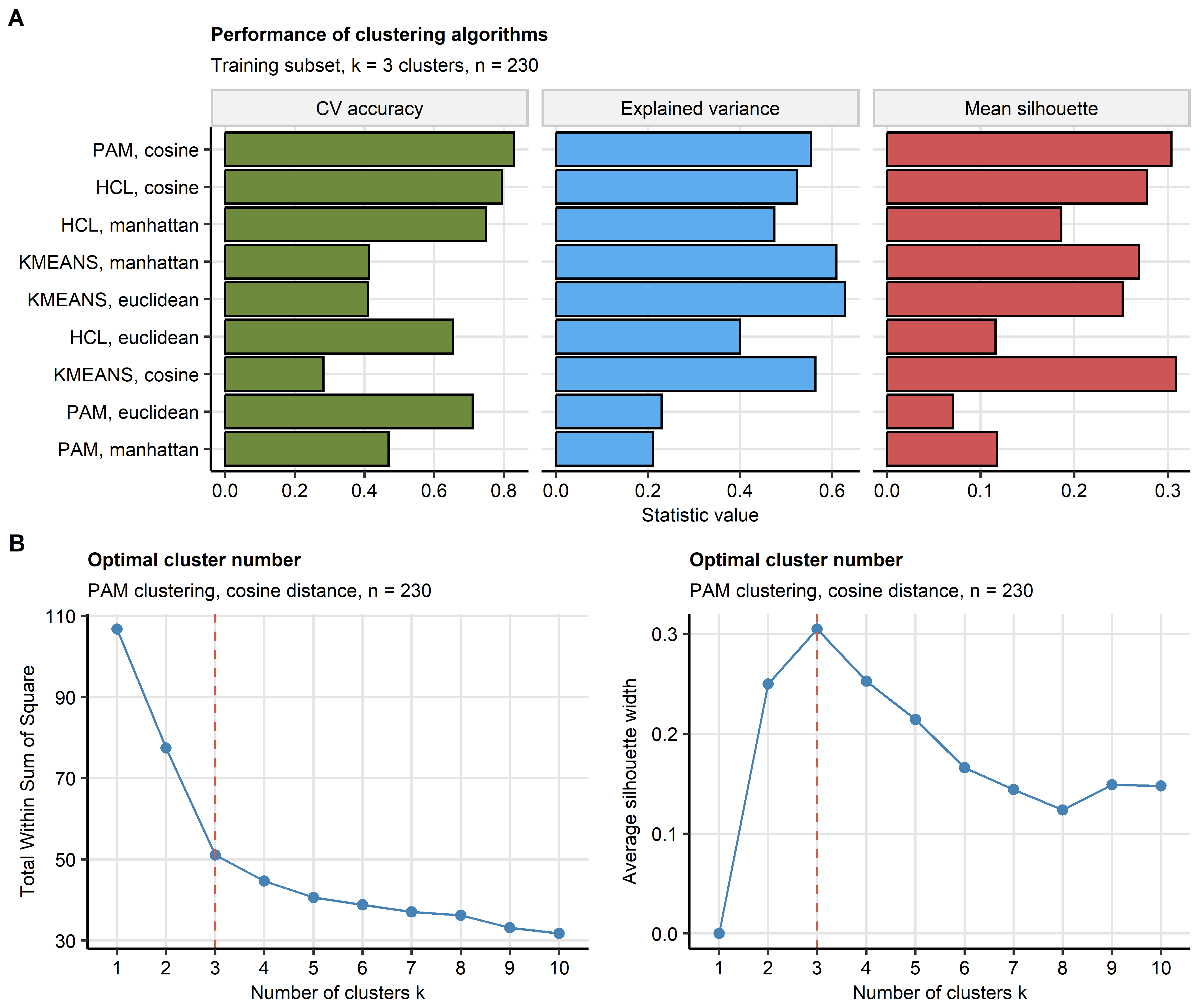


**Supplementary Figure S6. Definition of the mental health clusters in the training subset of the study cohort.**

*The mental health clusters were defined in respect to psychometric scoring in the training subset of the study cohort by PAM (partition around medoids) with cosine distance between the observations.*

*(A) Comparison of explanatory performance, cluster separation and reproducibility of various clustering algorithms in the training subset. The explanatory performance was measured as a fraction of explained clustering variance (ratio of between-cluster sum of squares to total sum of squares). The cluster separation was assessed with average silhouette width. The reproducibility was assessed by the rate of correct cluster assignment in 10-fold cross-validation (CV) with cluster assignment in the folds by an inverse distance weighted 27-nearest neighbors classifier. Note the superior stability of the PAM/cosine distance algorithm.*

*(B) Determination of the cluster number by the bend of the within-cluster sum of squares curve and the peak mean silhouette statistic.*


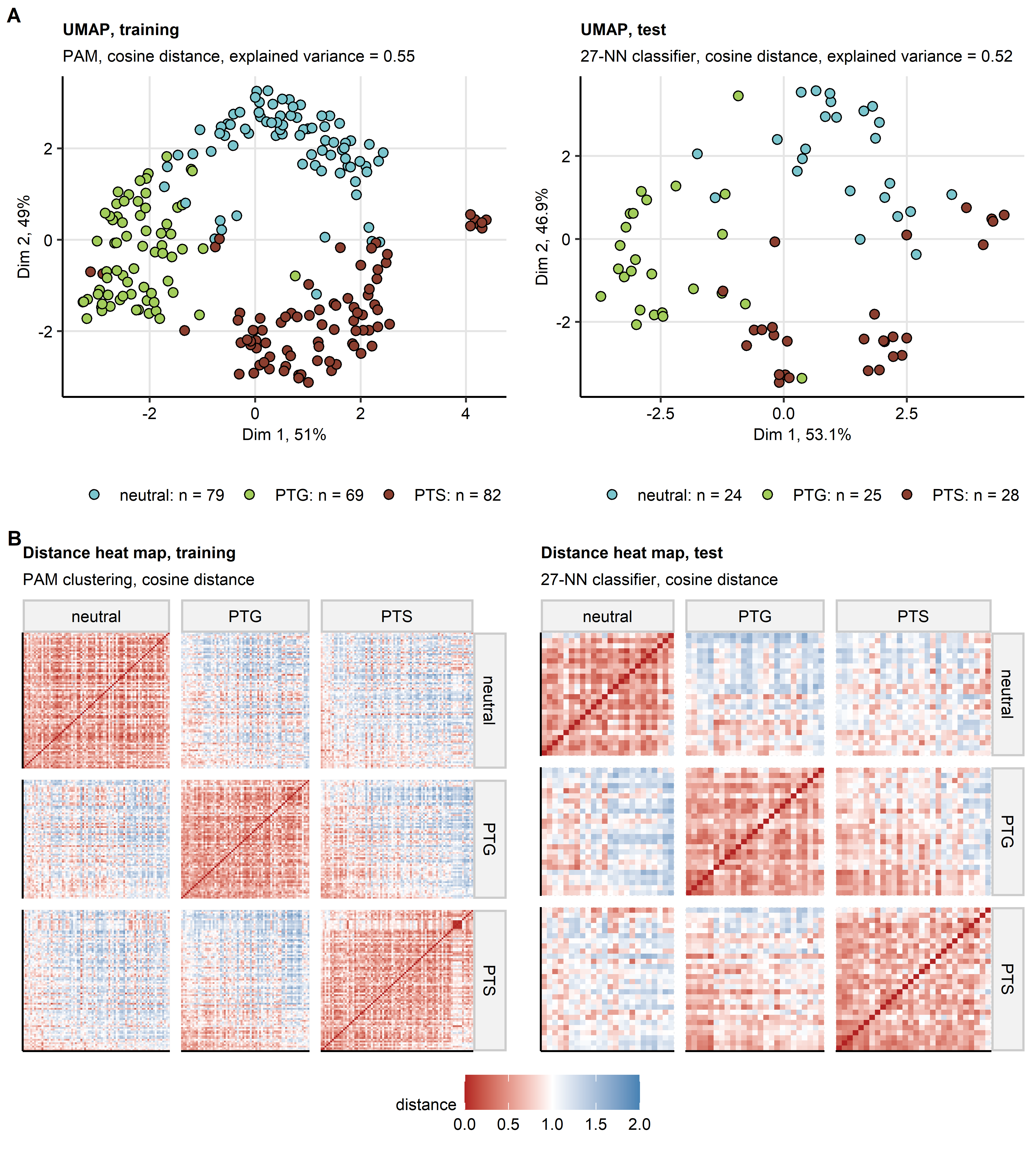


**Supplementary Figure S7. Semi-supervised clustering.**

*The mental health clusters were defined in respect to psychometric scoring in the training subset of the study cohort by PAM (partition around medoids) with cosine distance between the observations. Assignment of the test subset observations to the mental health clusters was done with the inverse distance weighted 27-nearest neighbors classifier. By this procedure, three mental health clusters were identified: neutral, PTG (post-traumatic growth) and PTS (post-traumatic stress). Numbers of observations in the mental health clusters are shown in the plot legend in (A).*

*(A) Observations in the training and test subsets of the study cohorts were subjected to two-dimensional UMAP (uniform manifold approximation and projection) in respect to the psychometric scores. UMAP layouts are shown in scatter plots. Points represent single samples. Point color codes for the cluster assignment.*

*(B) Pairwise cosine distances between observations in the mental health clusters of the training and test subsets presented in heat maps.*


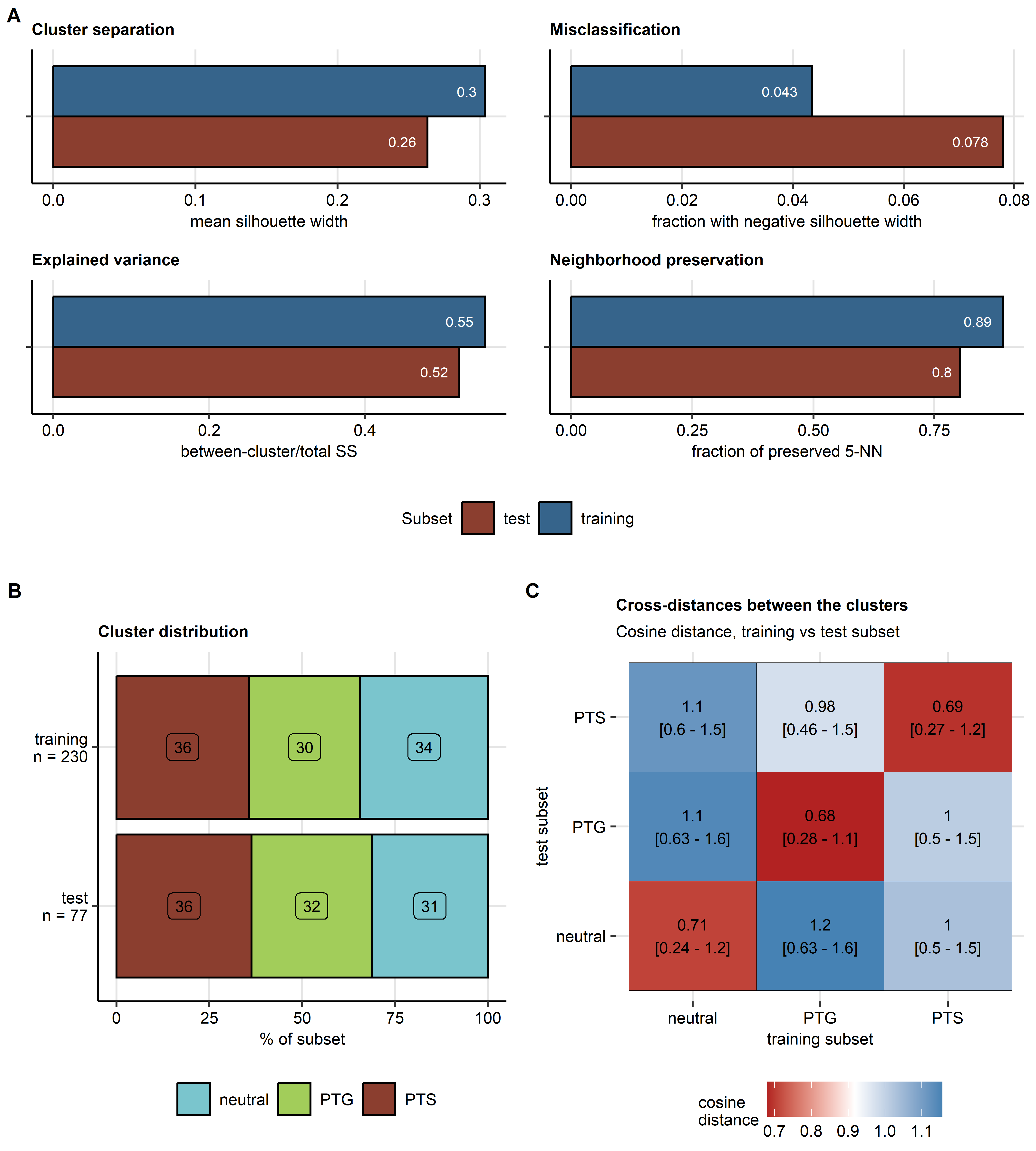


**Supplementary Figure S8. Distribution of the mental health clusters and cosine distances between the mental health clusters in the training and test subset of the study cohort.**

*Study cohort observations were assigned to the neutral, PTG (post-traumatic growth) and PTS (post-traumatic stress) mental health clusters by semi-supervised clustering as presented in Supplementary Figure S7.*

*(A) Comparison of numeric statistics of quality of clustering structures between the training and test subset of the study cohort. Mean silhouette width served as a measure of cluster separation. Fractions of observations with negative values of the silhouette width statistics were used to gauge misclassification rates. Explained clustering variance was defined as ratio of the total between-cluster sum of squares to the total sum of squares. Neighborhood preservation was measured by a fraction of the 5-nearest neighbors placed in the same cluster. SS: sum of squares, NN: nearest neighbors.*

*(B) Percentages of observations in the training and test subsets of the study cohort in the mental health clusters. Numbers of complete observations are indicated in the Y axis.*

*(C) Cosine cross-distances between the mental health clusters in the training and test subsets of the study data set visualized as a heat map. Average cross-distances with 95% percentile range are indicated in the tiles.*


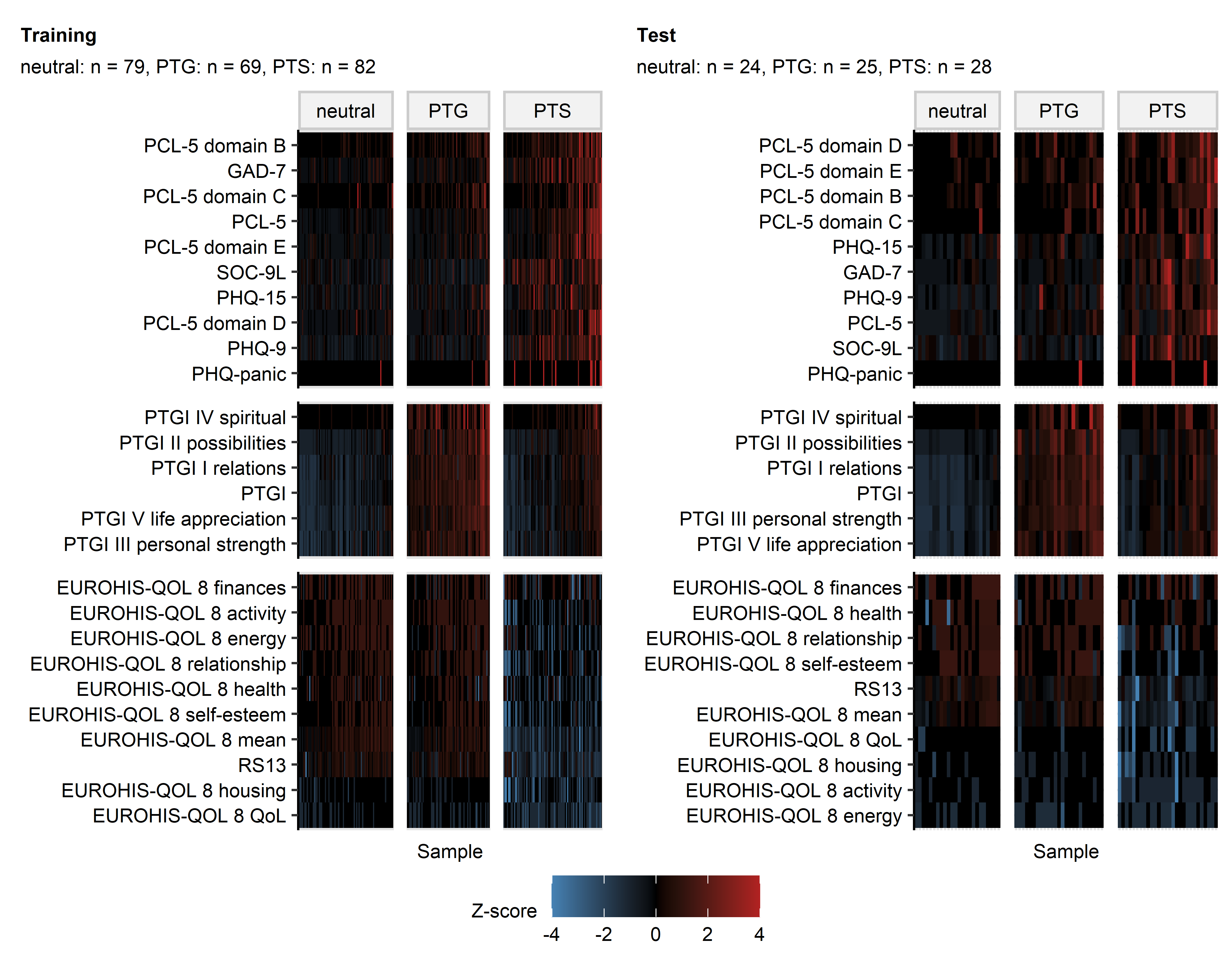


**Supplementary Figure S9. Levels of psychometric scores used for the cluster definition in the mental health clusters.**

*Levels of psychometric scores used in clustering in the mental health clusters. Normalized, median-centered score values (Z scores) are presented as heat maps. Numbers of observations in the clusters are displayed in the plot captions. GAD-7: 7-item general anxiety disorder scale; PHQ: patient health questionnaire; EUROHIS-QOL 8: 8-item EUROHIS project quality of life scale; SOC-9L: Leipzig 9-item sense of coherence questionnaire; RS13: 13-item resilience scale; PCL-5: PTSD checklist for DSM-5; PTGI: post-traumatic growth inventory; PTSD: post-traumatic stress disorder.*


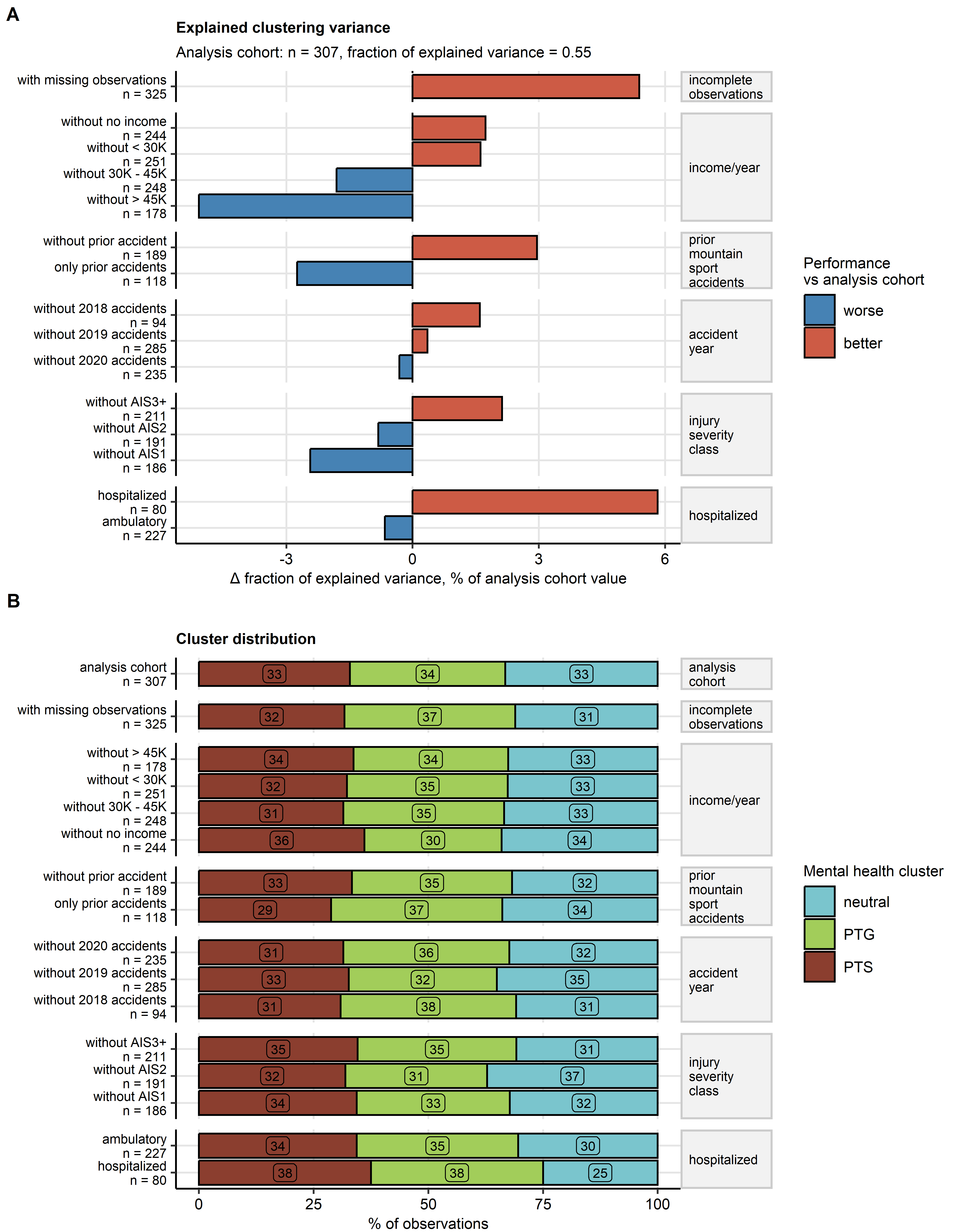


**Supplementary Figure S10. Effects of incomplete psychometry data, annual income, prior mountain sport accidents, injury severity, and hospitalization on development of the mental health clusters.**

*The mental health clusters were developed by PAM/cosine distance clustering for the analyzed study cohort, for the analyzed study cohort appended with participants with missing and imputed psychometric variables, participants with and without prior mountain sport accidents, hospitalized and non-hospitalized participants, the analysis cohort without each of no, low, intermediate and high annual income, the analysis cohort without each of 2018, 2019 and 2020 accidents, as well as for the analysis cohort with exclusion of participants with minor (abbreviated injury scale [AIS] 1), moderate (AIS2) and severe-to-critical injuries (AIS* $\geq$ *3). Fractions of explained clustering variance (ratio of total between-cluster sum of squares to total sum of squares) were compared between the mental health cluster assignment of the entire cohort and the mental clusters of collectives described above.*

*(A) Percent differences in fractions of explained variance between the mental health clusters of the analysis cohort and the mental health clusters in the analysis cohort appended with or depleted of the subsets indicated in the Y axis. Total numbers of observations are displayed in the Y axis.*

*(B) Distribution of the mental health clusters sizes expressed as percentages of all observations presented in a stack plot. Total observation numbers are displayed in the Y axis.*


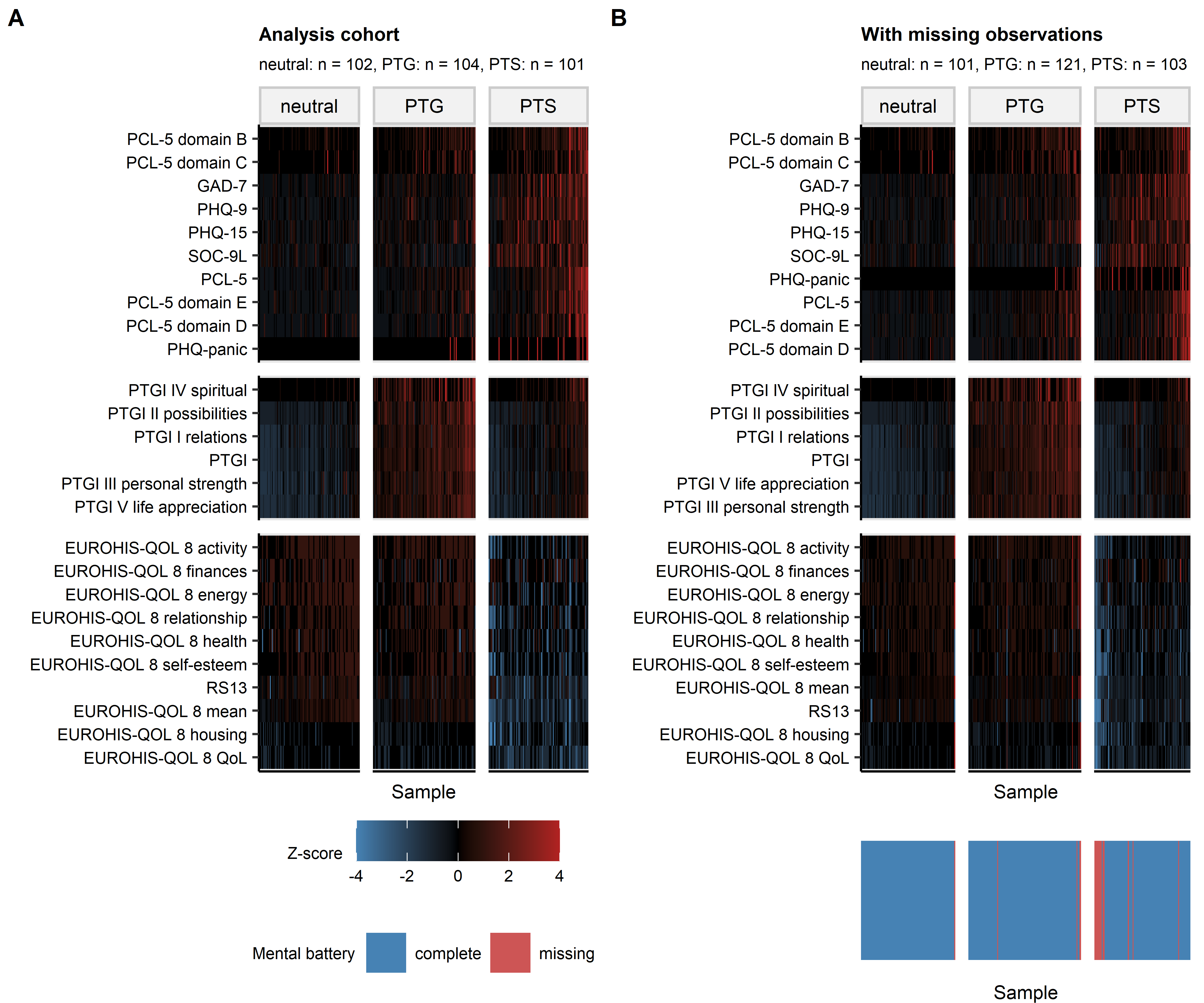


**Supplementary Figure S11. Mental health cluster assignment of participants excluded from the analysis due incomplete psychometry data.**

*Incomplete psychometry data were imputed for participants with < 80% missing answers in the mental health battery by a 9-nearest neighbors regressor. The mental health clusters were developed for separately the analysis cohort (A) and the analysis cohort appended with the imputed psychometry data (B). Normalized levels of the clustering variables (Z-scores) in the mental health clusters are visualized as heat maps. Assignment of the imputed observations to the mental health clusters is depicted in the color bar below the heat map in (B). Numbers of observations in the clusters are displayed in the plot captions.*


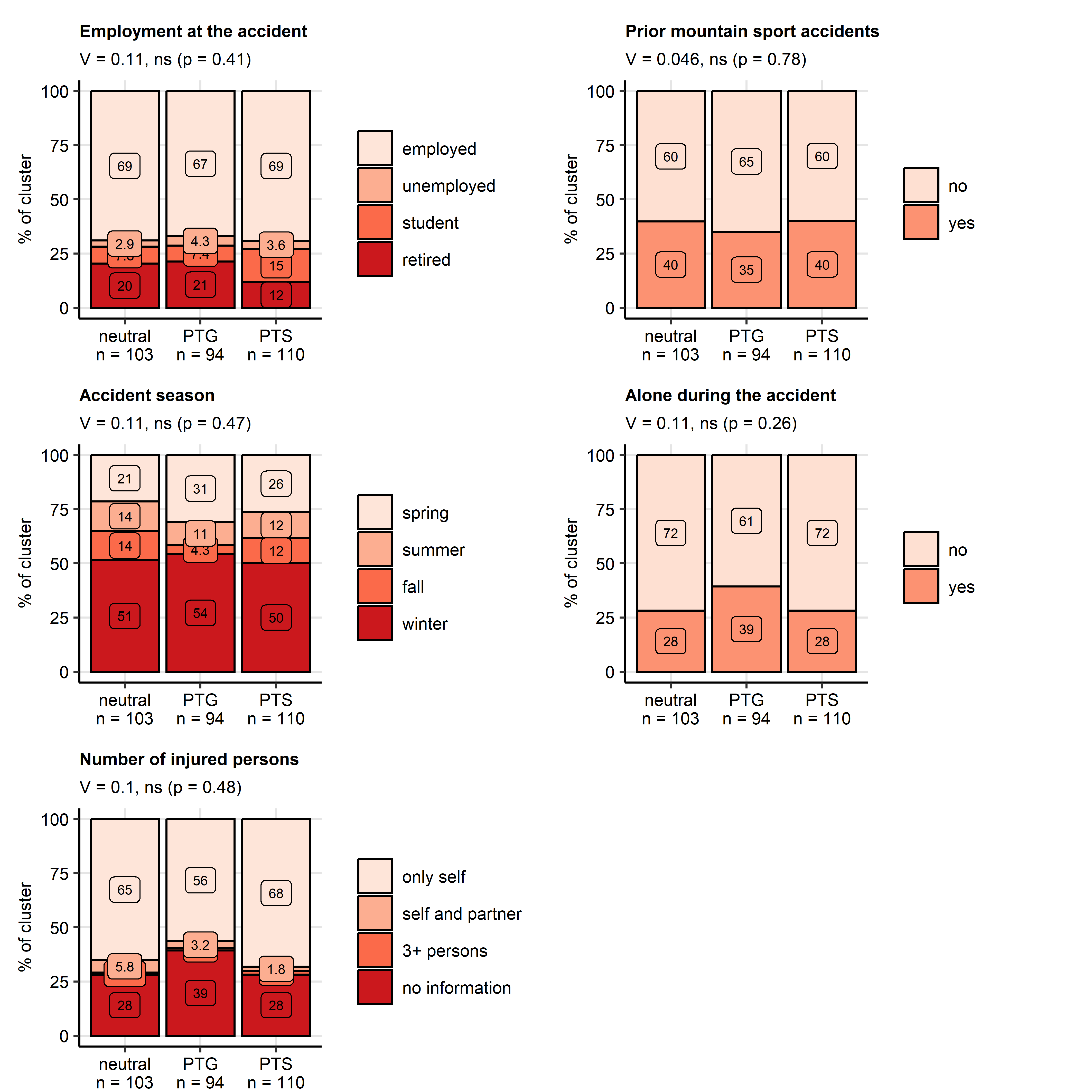


**Supplementary Figure S12. Employment status, prior mountain sport accidents, and accident details in the mental health clusters.**

*Distribution of employment status, frequency of mountain sport accidents in the past, distribution of the accidents across different seasons, frequency of being alone during the accident and number of injured persons in the mental health clusters. Statistical significance was determined by* $\chi^{2}$ *test with Cramer V effect size statistic. P values were corrected for multiple testing with the false discovery rate method. Percentages of variable’s categories in the entire study cohort are presented in stack plots. Effect sizes and p-values are displayed in the plot captions. Numbers of observations in the clusters are presented in the X axes.*


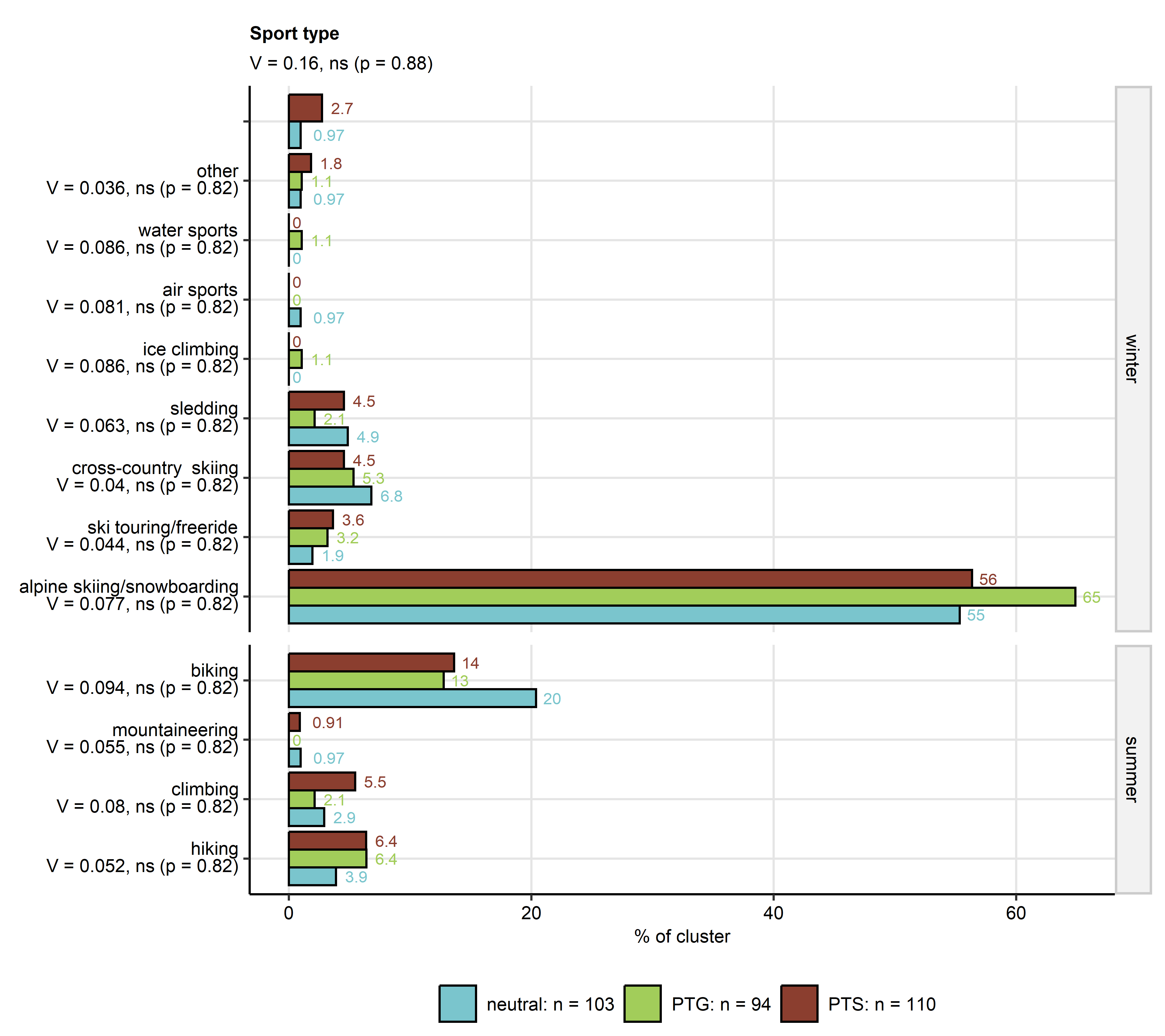


**Supplementary Figure S13. Mountain sport types in the clusters.**

*The global difference in distribution of accident sport types between the mental health clusters was compared by* $\chi^{2}$ *test with Cramer’s V effect size statistic. Post-hoc testing for differences in frequency of particular sport types between the clusters was done with Holm-corrected* $\chi^{2}$ *test with Cramer’s V effect size statistic. Percentages of sport types within the clusters are presented in bar plots. Effect size statistic and p value for the global difference are indicated in the plot caption. Results of post-hoc tests are displayed in the Y axis. Numbers of observations in the clusters are shown in the plot legend.*


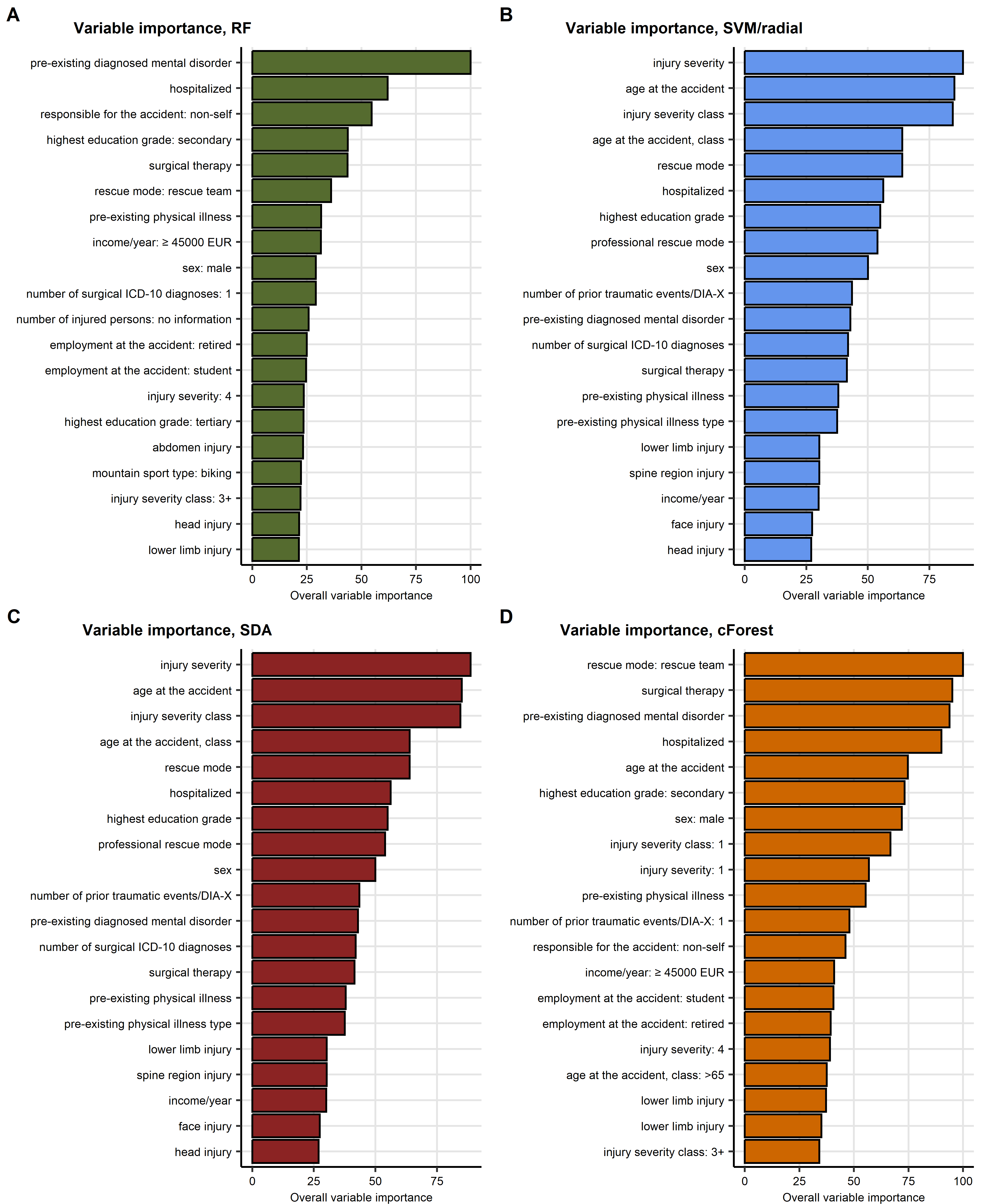


**Supplementary Figure S14. Variable importance metrics for the random forest, support vector machine, discriminant analysis, and conditional random forest algorithms. Predictors available during acute medical management of the patient.**

*Assignment to the mental health clusters was modeled with demographic, medical history and accident-related explanatory factors available during acute medical management of the patient. Psychometric variables used for cluster definition, mental disorder symptoms, resilience classes, presence and frequency of flashbacks and variables concerning recovery and long term consequences of the accident were excluded from the explanatory factor set. Variable importance metrics of machine learning algorithms with the best performance at predicting the mental health cluster assignment in the test subset of the study cohorts were computed. Importance metrics for the top 20 most important variables are presented as bar plots.* *(A) Random forest, (B) support vector machines with radial kernel, (C) shrinkage discriminant analysis, (D) conditional random forest.*


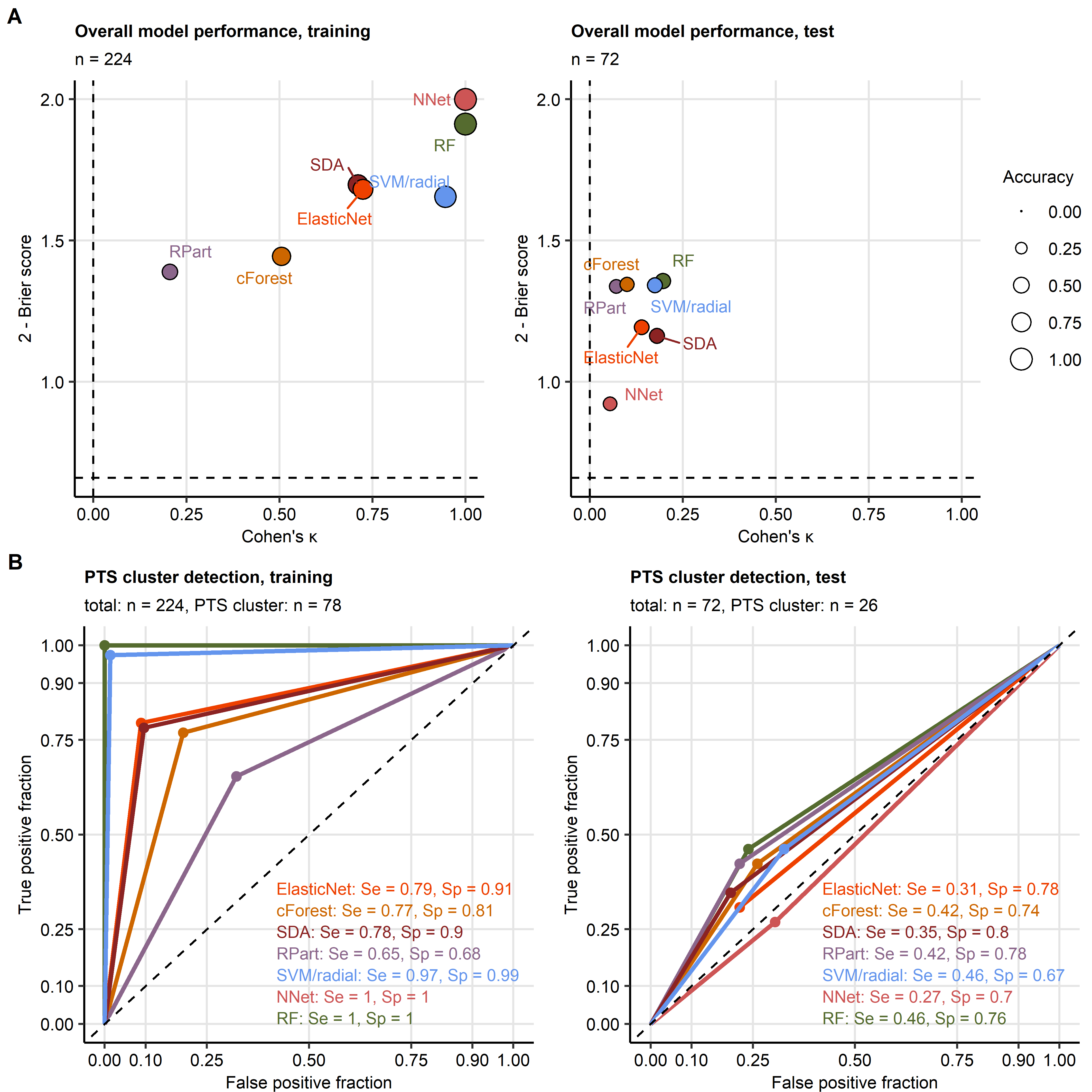


**Supplementary Figure S15. Assignment of accident victims to the mental health clusters based on explanatory factors available during acute medical management of the patient and long-term follow-up.**

*Assignment to the mental health clusters was modeled with demographic, medical history, accident- and recovery-related explanatory factors available during acute medical management of the patient and follow-up. Psychometric variables used for cluster definition, symptoms of mental disorders, resilience classes as well as presence and frequency of flashbacks were excluded from the explanatory factor set.*

*(A) Accuracy of the predicted Cluster assignment and predictive performance of the modeling algorithms was assessed by overall accuracy, Cohen’s* $\kappa$ *inter-rater accuracy metric (high values indicate good accuracy) and Brier score (low values indicate good performance) in the training and test subsets of the study cohort. Performance metrics are presented in scatter plots. Point size codes for the overall cluster assignment accuracy. Point color codes for the modeling algorithm. Numbers of complete observations are displayed in the plot captions.*

*(B) Sensitivity (Se) and specificity (Sp) of detection of participants assigned to the PTS cluster (post-traumatic stress) investigated by receiver-operating characteristic in the training and test subset of the study cohort. Sensitivity and specificity values are indicated in the plots. Line color codes for the modeling algorithm. Numbers of complete observations and observations in the PTS cluster are indicated in the plot captions.*

*RF: random forest; NNet: neural network with a single hidden layer; SVM/radial: support vector machines with radial kernel; RPart: recursive partitioning; SDA: shrinkage discriminant analysis; cForest: conditional random forest; Elastic Net: elastic net multinomial regression.*


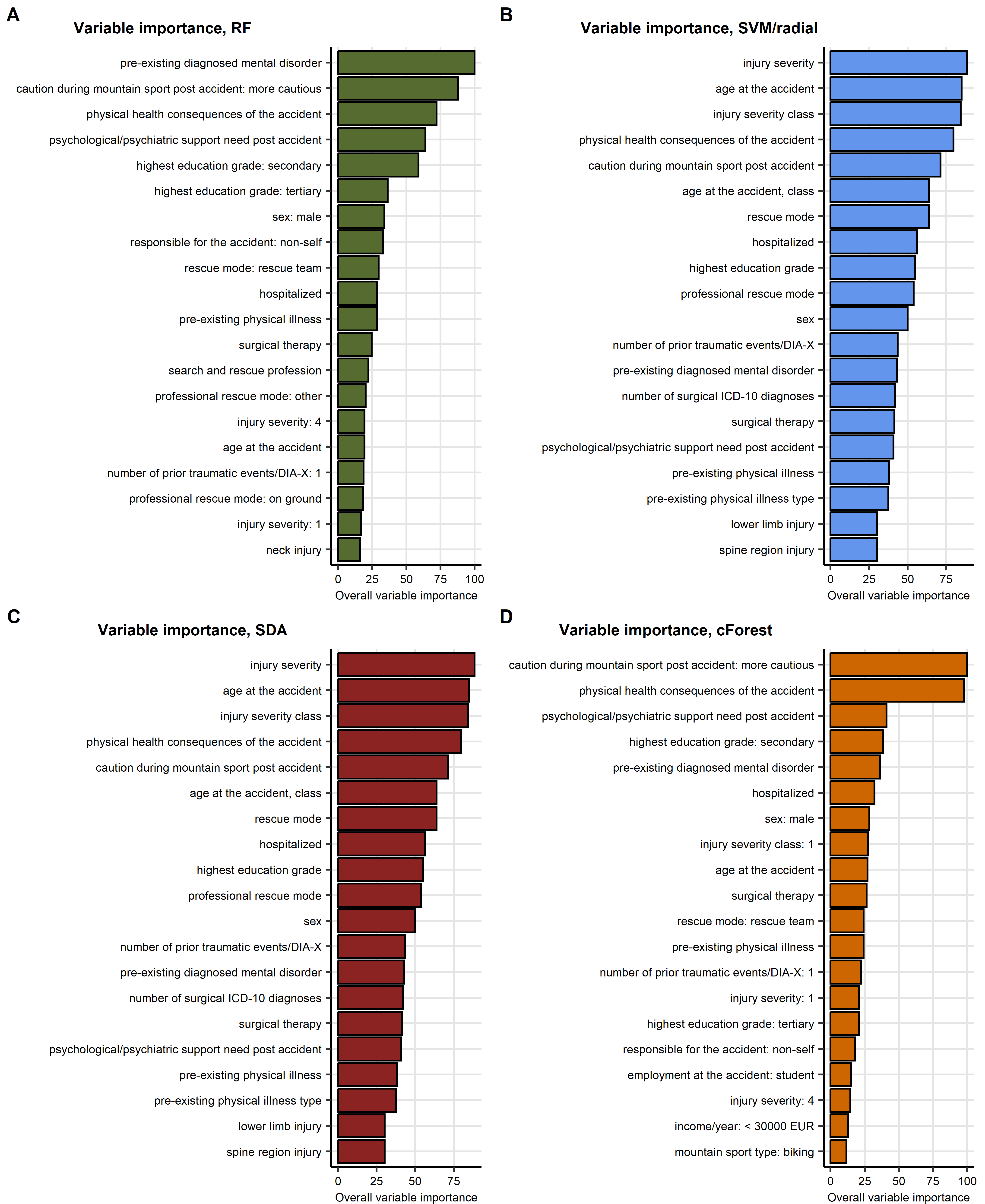


**Supplementary Figure S16. Variable importance metrics for the random forest, support vector machine, discriminant analysis, and conditional random forest algorithms. Predictors available during acute medical management of the patient and follow-up.**

*Assignment to the mental health clusters was modeled with demographic, medical history, accident- and recovery-related explanatory factors available during acute medical management of the patient and follow-up. Psychometric variables used for cluster definition, symptoms of mental disorders, resilience classes as well as presence and frequency of flashbacks were excluded from the explanatory factor set. Variable importance metrics of machine learning algorithms with the best performance at predicting the mental health cluster assignment in the test subset of the study cohorts were computed. Importance metrics for the top 20 most important variables are presented as bar plots.* *(A) Random forest, (B) support vector machines with radial kernel, (C) shrinkage discriminant analysis, (D) conditional random forest.*

# References

1. R Core Team, Bivand R, Carey VJ, DebRoy S, Eglen S, Guha R, Herbrandt S, Lewin-Koh N, Myatt M, Nelson M, et al. foreign: Read Data Stored by ’Minitab’, ’S’, ’SAS’, ’SPSS’, ’Stata’, ’Systat’, ’Weka’, ’dBase’, ... (2022) <https://cran.r-project.org/web/packages/foreign/index.html>

2. Wickham H, Averick M, Bryan J, Chang W, McGowan L, François R, Grolemund G, Hayes A, Henry L, Hester J, et al. Welcome to the Tidyverse. *Journal of Open Source Software* (2019) 4:1686. doi: [10.21105/joss.01686](https://doi.org/10.21105/joss.01686)

3. Henry L, Wickham Hadley. rlang: Functions for Base Types and Core R and ’Tidyverse’ Features. (2022) <https://cran.r-project.org/web/packages/rlang/index.html>

4. Gagolewski M, Tartanus B. Package ’stringi’. (2021) [https://cran.r-project.org/web/packages/stringi/index.html http://cran.ism.ac.jp/web/packages/stringi/stringi.pdf](https://cran.r-project.org/web/packages/stringi/index.html%20http://cran.ism.ac.jp/web/packages/stringi/stringi.pdf)

5. Hastie T, Tibshirani R, Narasimhan B, Chu G. impute: Imputation for microarray data. (2023) <https://bioconductor.org/packages/release/bioc/html/impute.html>

6. Kassambara A. rstatix: Pipe-Friendly Framework for Basic Statistical Tests. (2021) <https://cran.r-project.org/package=rstatix>

7. Signorell A. DescTools: Tools for Descriptive Statistics. (2022) <https://cran.r-project.org/package=DescTools>

8. Revelle W. Package ’psych’ - Procedures for Psychological, Psychometric and Personality Research. *R Package* (2015)1–358. [https://cran.r-project.org/web/packages/psych/index.html http://personality-project.org/r/psych-manual.pdf](https://cran.r-project.org/web/packages/psych/index.html%20http://personality-project.org/r/psych-manual.pdf)

9. Kassambara A, Mundt F. factoextra: Extract and Visualize the Results of Multivariate Data Analyses. (2020) <https://cran.r-project.org/web/packages/factoextra/index.html>

10. Schubert E, Rousseeuw PJ. Faster k-Medoids Clustering: Improving the PAM, CLARA, and CLARANS Algorithms. *Lecture notes in computer science (including subseries lecture notes in artificial intelligence and lecture notes in bioinformatics)*. Springer (2019). p. 171–187 doi: [10.1007/978-3-030-32047-8_16](https://doi.org/10.1007/978-3-030-32047-8_16)

11. Drost H-G. Philentropy: Information Theory and Distance Quantification with R. *Journal of Open Source Software* (2018) 3:765. doi: [10.21105/joss.00765](https://doi.org/10.21105/joss.00765)

12. Konopka T. umap: Uniform Manifold Approximation and Projection. (2022) <https://cran.r-project.org/web/packages/umap/index.html>

13. McInnes L, Healy J, Melville J. UMAP: Uniform Manifold Approximation and Projection for Dimension Reduction. (2018) [https://arxiv.org/abs/1802.03426v3 http://arxiv.org/abs/1802.03426](https://arxiv.org/abs/1802.03426v3%20http://arxiv.org/abs/1802.03426)

14. Breiman L. Random forests. *Machine Learning* (2001) 45:5–32. doi: [10.1023/A:1010933404324](https://doi.org/10.1023/A:1010933404324)

15. Wright MN, Ziegler A. ranger: A Fast Implementation of Random Forests for High Dimensional Data in C++ and R. *Journal of Statistical Software* (2017) 77:1–17. doi: [10.18637/JSS.V077.I01](https://doi.org/10.18637/JSS.V077.I01)

16. Ripley BD. *Pattern recognition and neural networks*. Cambridge University Press (2014). doi: [10.1017/CBO9780511812651](https://doi.org/10.1017/CBO9780511812651)

17. Weston J, Watkins C. Multi-Class Support Vector Machines. (1998)

18. Karatzoglou A, Hornik K, Smola A, Zeileis A. kernlab - An S4 Package for Kernel Methods in R. *Journal of Statistical Software* (2004) 11:1–20. doi: [10.18637/JSS.V011.I09](https://doi.org/10.18637/JSS.V011.I09)

19. Therneau TM, Atkinson B, Ripley BD. rpart: Recursive Partitioning and Regression Trees. (2022) <https://cran.r-project.org/web/packages/rpart/index.html>

20. Breiman L, Friedman JH, Olshen RA, Stone CJ. Classification and regression trees. *Classification and Regression Trees* (2017)1–358. doi: [10.1201/9781315139470/CLASSIFICATION-REGRESSION-TREES-LEO-BREIMAN](https://doi.org/10.1201/9781315139470/CLASSIFICATION-REGRESSION-TREES-LEO-BREIMAN)

21. Hothorn T, Hornik K, Zeileis A. Unbiased recursive partitioning: A conditional inference framework. *Journal of Computational and Graphical Statistics* (2006) 15:651–674. doi: [10.1198/106186006X133933](https://doi.org/10.1198/106186006X133933)

22. Strobl C, Boulesteix AL, Zeileis A, Hothorn T. Bias in random forest variable importance measures: Illustrations, sources and a solution. *BMC Bioinformatics* (2007) 8:1–21. doi: [10.1186/1471-2105-8-25/FIGURES/11](https://doi.org/10.1186/1471-2105-8-25/FIGURES/11)

23. Hothorn T, Hornik K, Strobl C, Zeileis A. party: A Laboratory for Recursive Partytioning. (2022) <https://cran.r-project.org/web/packages/party/index.html>

24. Ahdesmäki M, Strimmer K. Feature selection in omics prediction problems using cat scores and false nondiscovery rate control. *https://doiorg/101214/09-AOAS277* (2010) 4:503–519. doi: [10.1214/09-AOAS277](https://doi.org/10.1214/09-AOAS277)

25. Ahdesmaki M, Zuber V, Gibb S, Strimmer K. sda: Shrinkage Discriminant Analysis and CAT Score Variable Selection. (2022) <https://cran.r-project.org/web/packages/sda/index.html>

26. Zou H, Hastie T. Regularization and variable selection via the elastic net. *Journal of the Royal Statistical Society Series B: Statistical Methodology* (2005) 67:301–320. doi: [10.1111/j.1467-9868.2005.00503.x](https://doi.org/10.1111/j.1467-9868.2005.00503.x)

27. Friedman J, Hastie T, Tibshirani R. Regularization paths for generalized linear models via coordinate descent. *Journal of Statistical Software* (2010) 33:1–22. doi: [10.18637/jss.v033.i01](https://doi.org/10.18637/jss.v033.i01)

28. Kuhn M. Building predictive models in R using the caret package. *Journal of Statistical Software* (2008) 28:1–26. doi: [10.18637/jss.v028.i05](https://doi.org/10.18637/jss.v028.i05)

29. Wickham Hadley. *ggplot2: Elegant Graphics for Data Analysis*. 1st ed. New York: Springer-Verlag (2016). [https://ggplot2.tidyverse.org](https://ggplot2.tidyverse.org/)

30. Sachs MC. Plotroc: A tool for plotting ROC curves. *Journal of Statistical Software* (2017) 79:1–19. doi: [10.18637/jss.v079.c02](https://doi.org/10.18637/jss.v079.c02)

31. Krassowski M. ComplexUpset: Create Complex UpSet Plots Using ’ggplot2’ Components. (2021) <https://cran.r-project.org/web/packages/ComplexUpset/index.html>

32. Wilke CO. *Fundamentals of Data Visualization: A Primer on Making Informative and Compelling Figures*. 1st ed. Sebastopol: O’Reilly Media (2019).

33. Pedersen TL. patchwork: The Composer of Plots. (2023) <https://cran.r-project.org/web/packages/patchwork/index.html>

34. Gohel D. flextable: Functions for Tabular Reporting. (2022) <https://cran.r-project.org/web/packages/flextable/index.html>

35. Allaire J, Xie Y, McPherson J, Luraschi J, Ushey K, Atkins A, Wickham H, Cheng J. rmarkdown: Dynamic Documents for R. (2022) <https://cran.r-project.org/web/packages/rmarkdown/index.html>

36. Xie Y. *Bookdown: Authoring books and technical documents with R Markdown*. (2016). doi: [10.1201/9781315204963](https://doi.org/10.1201/9781315204963)

37. Xie Y. knitr: A General-Purpose Package for Dynamic Report Generation in R. (2022) <https://cran.r-project.org/web/packages/knitr/index.html>

38. Spitzer RL, Kroenke K, Williams JBW, Löwe B. A Brief Measure for Assessing Generalized Anxiety Disorder: The GAD-7. *Archives of Internal Medicine* (2006) 166:1092–1097. doi: [10.1001/ARCHINTE.166.10.1092](https://doi.org/10.1001/ARCHINTE.166.10.1092)

39. Löwe B, Spitzer RL, Zipfel S, Herzog W. Auflage Manual 17.07. (2002).

40. Gräfe K, Zipfel S, Herzog W, Löwe B. Screening psychischer störungen mit dem "Gesundheitsfragebogen für Patienten (PHQ-D)". Ergebnisse der Deutschen validierungsstudie. *Diagnostica* (2004) 50:171–181. doi: [10.1026/0012-1924.50.4.171](https://doi.org/10.1026/0012-1924.50.4.171)

41. Kroenke K, Spitzer RL. The PHQ-9: A new depression diagnostic and severity measure. *Psychiatric Annals* (2002) 32:509–515. doi: [10.3928/0048-5713-20020901-06](https://doi.org/10.3928/0048-5713-20020901-06)

42. Kroenke K, Spitzer RL, Williams JBW. The PHQ-15: validity of a new measure for evaluating the severity of somatic symptoms. *Psychosomatic medicine* (2002) 64:258–266. doi: [10.1097/00006842-200203000-00008](https://doi.org/10.1097/00006842-200203000-00008)

43. Leppert K, Koch B, Brähler E, Und BS-KD, 2008 U. Die Resilienzskala (RS)–Überprüfung der Langform RS-25 und einer Kurzform RS-13. *Klinische Diagnostik und Evaluation* (2008) 1:226–243. <https://www.academia.edu/download/44388154/A_406.pdf>

44. Schumacher J, Wilz G, Gunzelmann T, Brähler E. Die sense of coherence scale von antonovsky: Teststatische überprüfung in einer repräsentativen bevölkerungsstichprobe und konstruktion einer kurzskala. *PPmP Psychotherapie Psychosomatik Medizinische Psychologie* (2000) 50:472–482. doi: [10.1055/s-2000-9207](https://doi.org/10.1055/s-2000-9207)

45. Schmidt S, Mühlan H, Power M. The EUROHIS-QOL 8-item index: psychometric results of a cross-cultural field study. *European Journal of Public Health* (2006) 16:420–428. doi: [10.1093/EURPUB/CKI155](https://doi.org/10.1093/EURPUB/CKI155)

46. Tedeschi RG, Calhoun LG. The Posttraumatic Growth Inventory: measuring the positive legacy of trauma. *Journal of traumatic stress* (1996) 9:455–471. doi: [10.1007/BF02103658](https://doi.org/10.1007/BF02103658)

47. Bovin MJ, Marx BP, Weathers FW, Gallagher MW, Rodriguez P, Schnurr PP, Keane TM. Psychometric properties of the PTSD Checklist for Diagnostic and Statistical Manual of Mental Disorders-Fifth Edition (PCL-5) in veterans. *Psychological assessment* (2016) 28:1379–1391. doi: [10.1037/PAS0000254](https://doi.org/10.1037/PAS0000254)

48. Humer E, Schaffler Y, Jesser A, Probst T, Pieh C. Mental health in the Austrian general population during COVID-19: Cross-sectional study on the association with sociodemographic factors. *Frontiers in Psychiatry* (2022) 13: doi: [10.3389/FPSYT.2022.943303/FULL](https://doi.org/10.3389/FPSYT.2022.943303/FULL)

49. Maercker A, Bromberger F. Checklisten und Fragebogen zur Erfassung traumatischer Ereignisse in deutscher Sprache. *Trierer Psychologische Berichte* (2005) 32:

50. O’Brien CP. The CAGE Questionnaire for Detection of Alcoholism. *JAMA* (2008) 300:2054–2056. doi: [10.1001/JAMA.2008.570](https://doi.org/10.1001/JAMA.2008.570)

51. Gennarelli TA, Wodzin E. AIS 2005: A contemporary injury scale. *Injury* (2006) 37:1083–1091. doi: [10.1016/j.injury.2006.07.009](https://doi.org/10.1016/j.injury.2006.07.009)

52. Österreichisches Kuratorium für Alpine Sicherheit. Alpinunfälle in Österreich 2023 – Jahresrückblick. (2024) <https://alpinesicherheit.at/alpinunfaelle-2023-rueckblick/> [Accessed February 5, 2024]

53. Statistik Austria. Bevölkerung im Jahresdurchschnitt - STATISTIK AUSTRIA. (2023) <https://www.statistik.at/fileadmin/pages/404/Bev_Alter_Geschlecht_Staatsangeh_Bundesl_Zeitreihe.ods> [Accessed February 6, 2024]

54. Statistik Austria. Bildung in Zahlen - STATISTIK AUSTRIA. (2020) <https://www.statistik.at/fileadmin/pages/1865/Bildung_in_Zahlen_2021_22_Tabellen.zip> [Accessed February 6, 2024]

55. Statistik Austria. Erwerbstätige - Merkmale - STATISTIK AUSTRIA. (2021) <https://www.statistik.at/fileadmin/pages/54/RZ2021Erwerbstaetigkeit.ods> [Accessed February 6, 2024]

56. Statistik Austria. Rauchen - STATISTIK AUSTRIA. (2019) <https://www.statistik.at/fileadmin/pages/382/Rauchen_ATHIS2019.ods> [Accessed February 6, 2024]

57. Statistik Austria. Gesundheitszustand selbstberichtet - STATISTIK AUSTRIA. (2019) <https://www.statistik.at/fileadmin/pages/393/Gesundheitszustand_selbstberichtet_ATHIS2019.ods> [Accessed February 6, 2024]

58. Hauffa R, Rief W, Brähler E, Martin A, Mewes R, Glaesmer H. Lifetime traumatic experiences and posttraumatic stress disorder in the German population: Results of a representative population survey. *Journal of Nervous and Mental Disease* (2011) 199:934–939. doi: [10.1097/NMD.0B013E3182392C0D](https://doi.org/10.1097/NMD.0B013E3182392C0D)

59. Darves-Bornoz JM, Alonso J, Girolamo G de, Graaf R de, Haro JM, Koves-Masfety V, Lepine JP, Nachbaur G, Negre-Pages L, Vilagut G, et al. Main traumatic events in Europe: PTSD in the European study of the epidemiology of mental disorders survey. *Journal of Traumatic Stress* (2008) 21:455–462. doi: [10.1002/JTS.20357](https://doi.org/10.1002/JTS.20357)

60. Kilpatrick DG, Resnick HS, Milanak ME, Miller MW, Keyes KM, Friedman MJ. National Estimates of Exposure to Traumatic Events and PTSD Prevalence Using DSM-IV and DSM-5 Criteria. *Journal of Traumatic Stress* (2013) 26:537–547. doi: [10.1002/JTS.21848](https://doi.org/10.1002/JTS.21848)

61. Koenen KC, Ratanatharathorn A, Ng L, McLaughlin KA, Bromet EJ, Stein DJ, Karam EG, Meron Ruscio A, Benjet C, Scott K, et al. Posttraumatic stress disorder in the World Mental Health Surveys. *Psychological medicine* (2017) 47:2260. doi: [10.1017/S0033291717000708](https://doi.org/10.1017/S0033291717000708)

62. Kessler RC, Aguilar-Gaxiola S, Alonso J, Benjet C, Bromet EJ, Cardoso G, Degenhardt L, Girolamo G de, Dinolova RV, Ferry F, et al. Trauma and PTSD in the WHO World Mental Health Surveys. *European Journal of Psychotraumatology* (2017) 8:5. doi: [10.1080/20008198.2017.1353383](https://doi.org/10.1080/20008198.2017.1353383)

63. Mikutta C, Schmid JJ, Ehlert U. Resilience and Post-traumatic Stress Disorder in the Swiss Alpine Rescue Association. *Frontiers in psychiatry* (2022) 13: doi: [10.3389/FPSYT.2022.780498](https://doi.org/10.3389/FPSYT.2022.780498)

64. Chernova A, Frajo-Apor B, Pardeller S, Tutzer F, Plattner B, Haring C, Holzner B, Kemmler G, Marksteiner J, Miller C, et al. The Mediating Role of Resilience and Extraversion on Psychological Distress and Loneliness Among the General Population of Tyrol, Austria Between the First and the Second Wave of the COVID-19 Pandemic. *Frontiers in Psychiatry* (2021) 12:766261. doi: [10.3389/FPSYT.2021.766261/FULL](https://doi.org/10.3389/FPSYT.2021.766261/FULL)

65. McDonald RP. *Test theory: A unified treatment*. 1st Editio. New Yor: Psychology Press (1999). doi: [10.4324/9781410601087](https://doi.org/10.4324/9781410601087)

66. BARTLETT MS. THE STATISTICAL CONCEPTION OF MENTAL FACTORS. *British Journal of Psychology General Section* (1937) 28:97–104. doi: [10.1111/j.2044-8295.1937.tb00863.x](https://doi.org/10.1111/j.2044-8295.1937.tb00863.x)

67. Cohen S, Kamarck T, Mermelstein R. A global measure of perceived stress. *Journal of health and social behavior* (1983) 24:385–396. doi: [10.2307/2136404](https://doi.org/10.2307/2136404)

68. Sinclair VG, Wallston KA. The development and psychometric evaluation of the Brief Resilient Coping Scale. *Assessment* (2004) 11:94–101. doi: [10.1177/1073191103258144](https://doi.org/10.1177/1073191103258144)

69. Hopkins B, Skellam JG. A New Method for determining the Type of Distribution of Plant Individuals. *Annals of Botany* (1954) 18:213–227. doi: [10.1093/OXFORDJOURNALS.AOB.A083391](https://doi.org/10.1093/OXFORDJOURNALS.AOB.A083391)

70. Meyer D, Buchta C. proxy: Distance and Similarity Measures. (2022) <https://cran.r-project.org/web/packages/proxy/index.html>

71. Benjamini Y, Hochberg Y. Controlling the False Discovery Rate: A Practical and Powerful Approach to Multiple Testing. *Journal of the Royal Statistical Society: Series B (Methodological)* (1995) 57:289–300. doi: [10.1111/j.2517-6161.1995.tb02031.x](https://doi.org/10.1111/j.2517-6161.1995.tb02031.x)

72. Cohen J. A Coefficient of Agreement for Nominal Scales. *Educational and Psychological Measurement* (1960) 20:37–46. doi: [10.1177/001316446002000104](https://doi.org/10.1177/001316446002000104)

73. McHugh ML. Interrater reliability: the kappa statistic. *Biochemia Medica* (2012) 22:276. doi: [10.11613/bm.2012.031](https://doi.org/10.11613/bm.2012.031)

74. Field AP. Discovering statistics using IBM SPSS Statistics: and sex and drugs and rock ‘n’ roll, 4th edition. *Choice Reviews Online* (2013) 50:xviii, 908, xxxvi. <http://www.uk.sagepub.com/field4e/default.htm>

75. Cohen J. Statistical Power Analysis for the Behavioral Sciences. *Statistical Power Analysis for the Behavioral Sciences* (2013) doi: [10.4324/9780203771587](https://doi.org/10.4324/9780203771587)

76. Rousseeuw PJ. Silhouettes: A graphical aid to the interpretation and validation of cluster analysis. *Journal of Computational and Applied Mathematics* (1987) 20:53–65. doi: [10.1016/0377-0427(87)90125-7](https://doi.org/10.1016/0377-0427(87)90125-7)

77. Lange T, Roth V, Braun ML, Buhmann JM. Stability-based validation of clustering solutions. *Neural Computation* (2004) 16:1299–1323. doi: [10.1162/089976604773717621](https://doi.org/10.1162/089976604773717621)

78. Venna J, Kaski S. Neighborhood preservation in nonlinear projection methods: An experimental study. *Lecture Notes in Computer Science (including subseries Lecture Notes in Artificial Intelligence and Lecture Notes in Bioinformatics)* (2001) 2130:485–491. doi: [10.1007/3-540-44668-0_68](https://doi.org/10.1007/3-540-44668-0_68)

79. Thabane L, Mbuagbaw L, Zhang S, Samaan Z, Marcucci M, Ye C, Thabane M, Giangregorio L, Dennis B, Kosa D, et al. A tutorial on sensitivity analyses in clinical trials: the what, why, when and how. *BMC Medical Research Methodology 2013 13:1* (2013) 13:1–12. doi: [10.1186/1471-2288-13-92](https://doi.org/10.1186/1471-2288-13-92)

80. Brier GW. VERIFICATION OF FORECASTS EXPRESSED IN TERMS OF PROBABILITY. *Monthly Weather Review* (1950) 78:1–3. doi: [10.1175/1520-0493(1950)078<0001:vofeit>2.0.co;2](https://doi.org/10.1175/1520-0493(1950)078%3c0001:vofeit%3e2.0.co;2)

81. Garson GD. Interpreting neural-network connection weights. *AI Expert* (1991) 6:47–51. doi: [10.5555/129449.129452](https://doi.org/10.5555/129449.129452)
